# Integrated QSAR, Molecular Docking, ADMET Profiling, and Antioxidant Evaluation of Substituted Chromone and Aryloxyalkanoic Acid Derivatives as Potential CysLT_1_ Receptor Antagonists

**DOI:** 10.3390/ph19040600

**Published:** 2026-04-08

**Authors:** Mahboob Alam

**Affiliations:** Department of Safety Engineering, Dongguk University WISE, 123 Dongdae-ro, Gyeongju-si 780714, Gyeongsangbuk-do, Republic of Korea; mahboobchem@gmail.com

**Keywords:** QSAR modeling (2D and 3D), ADMET, CysLT_1_ receptor antagonists, molecular docking, antioxidant activity

## Abstract

**Background:** Cysteinyl leukotrienes are components of slow-reacting substances of anaphylactic shock (SRS-A) and play a key role in asthma and inflammatory responses. Although chromone-2-carboxylic acids and substituted (aryloxy)alkanoic acids have the potential to be SRS-A antagonists, their comprehensive structure–activity relationships and pharmacokinetic characteristics remain understudied. **Objective:** This study integrated computational and experimental approaches, including QSAR modeling, molecular docking, ADMET analysis, molecular dynamics (MD) simulations, and antioxidant evaluation to identify and prioritize bifunctional compounds with anti-inflammatory and free radical-scavenging properties. **Methods:** A set of 68 compounds was analyzed using 2D and 3D quantitative structure–activity relationships (QSAR) (MLR, MNLR, SVR, ANN, and atom-based partial least squares). Molecular docking and 100 ns MD simulations were performed against the CysLT_1_ receptor (PDB ID: 6RZ5). ADMET and drug-like properties of the compounds were predicted using ADMETlab 2.0 and SwissADME, and the in vitro antioxidant activity of the top-ranked compounds was evaluated using the DPPH method. **Results:** The atom-based 3D-QSAR model showed strong predictive power (R^2^ = 0.9524, Q^2^ = 0.5382). Compounds **25**, **41**, and **47** stood out with the most significant binding energies: −9.5 kcal/mol for 25, −10.0 kcal/mol for **41**, and −9.4 kcal/mol for 47. MD simulations confirmed the structural stability and consistent interactions of the protein-compound **47** complex. ADMET analysis showed that compounds **25** and **41** had good pharmacokinetic properties, and in vitro antioxidant assays verified their free radical-scavenging efficacy. **Conclusion:** Our results highlight the utility of an integrated computational–experimental strategy for the discovery of dual-acting SRS-A antagonists. Compound 25 is highlighted as a promising lead compound for further preclinical development, which effectively combines leukotriene receptor antagonism and antioxidant activity. This framework provides an effective strategy for prioritizing lead compounds in anti-inflammatory drug development.

## 1. Introduction

Leukotrienes (LTs), particularly LTC_4_, LTD_4_, and LTE_4_, are potent lipid mediators derived from arachidonic acid that play a crucial role in inflammation and allergic responses [1,2]. These molecules contribute to bronchoconstriction, increased vascular permeability, and immune cell recruitment, making them key targets in the management of conditions such as asthma and anaphylaxis [3,4,5]. The slow-reacting substance of anaphylaxis (SRS-A) was historically identified as a mediator responsible for prolonged smooth muscle contractions, later confirmed to consist of cysteinyl leukotrienes (CysLTs) [6,7]. Given their profound impact on smooth muscle physiology, antagonists targeting CysLT receptors, particularly the CysLT_1_ receptor, have been extensively explored for therapeutic potential. This is particularly relevant to our study, which focuses on substituted chromone and (aryloxy)alkanoic acid derivatives. Previous pharmacological studies have demonstrated that several (aryloxy)alkanoic acid analogs and structurally related compounds act as potent antagonists of the CysLT_1_ receptor or SRS-A, providing strong support for their therapeutic potential in inflammatory disorders [8,9,10,11]. These compounds were collected from previously reported medicinal chemistry studies by LeMahieu et al. [8] and Tilley et al. [11]. These studies described their synthesis and pharmacological evaluation as leukotriene or SRS-A antagonists. We selected these known compounds based on their therapeutic relevance and aimed to investigate their molecular features using an integrated in silico and experimental approach.

The traditional trial-and-error approach in developing new drugs is often tedious, costly, and time-consuming. To address these limitations and accelerate the identification of promising drug candidates, in silico modeling approaches such as quantitative structure–activity relationships (QSAR), molecular docking, and ADMET studies offer a powerful alternative. These computational approaches efficiently filter hits-to-leads, significantly reducing the time and cost associated with synthesizing new compounds [12,13,14,15,16,17].

Quantitative Structure–Activity Relationship (QSAR) modeling has emerged as a powerful computational approach to predict the biological activity of chemical compounds based on their molecular properties [18]. By establishing mathematical relationships between structural descriptors and bioactivity (e.g., IC_50_ values), QSAR studies facilitate the rational design and optimization of lead compounds for specific pharmacological targets. In the context of SRS-A-mediated contractions, QSAR can provide valuable insights into the structural features influencing antagonist potency, thereby guiding the selection of the most effective compounds for further biological evaluation [19].

In this study, a QSAR approach is employed to analyze 68 compounds (Table 1) with experimentally determined IC_50_ values against SRS-A-induced contractions [8,11]. The goal is to identify key molecular descriptors, including electronic, steric, and hydrophobic properties, that correlate with antagonist potency. Based on the QSAR model, the compound with the best performance was selected for further investigation of its antioxidant potential. Given the interplay between oxidative stress and inflammatory pathways, understanding the antioxidant capacity of these antagonists may provide additional therapeutic insights beyond the inhibition of leukotriene receptors.

The significance of this research lies in its integrative approach, which combines computational modeling and experimental validation to streamline the drug discovery process. By leveraging QSAR analysis, this study not only enhances the understanding of structural determinants of leukotriene antagonism but also paves the way for identifying compounds with dual anti-inflammatory and antioxidant properties (Table 1). Chemical structures of substituted chromone and aryloxyalkanoic acid derivatives are analyzed in this study. Compounds were collected from published sources [8,11] and are not hypothetical. Structure numbers correspond to those used throughout the QSAR and docking analysis.

To overcome the limitations of existing leukotriene receptor antagonists such as zafirlukast, this study integrates a comprehensive in silico approach combined with experimental antioxidant evaluation of selected compounds. A total of 68 previously reported substituted chromone and (aryloxy)alkanoic acid derivatives were analyzed. Unlike many earlier studies that focused solely on receptor binding or quantitative structure–activity relationship (QSAR) modeling, this work combines classical and machine learning-based QSAR techniques including 2D multivariate linear regression (MLR), multivariate nonlinear regression (MNLR), support vector regression (SVR), artificial neural networks (ANN), and atom-based 3D QSAR alongside molecular docking, molecular dynamics simulations, and ADMET analysis. Zafirlukast, a clinically established CysLT_1_ antagonist with reported antioxidant activity, was redocked as a reference compound to validate the docking protocol. Among the screened compounds, compounds **25** and **41** exhibited strong binding affinities to CysLT_1_ and were further evaluated for antioxidant potential using a DPPH assay. Both compounds demonstrated free radical scavenging activity comparable to or greater than that of zafirlukast, highlighting their promise as dual-function agents targeting both leukotriene signaling and oxidative stress pathways in inflammatory diseases.

## 2. Results and Discussion

A total of 633 molecular descriptors (1D, 2D, and 3D) were calculated using ChemDes. The variable reduction process and stepwise regression analyses were then performed to remove the inappropriate molecular descriptors [20,21]. To avoid model over-fitting, an 8–10:1 ratio between the levels of compounds and molecular descriptors was employed. After performing stepwise regression, descriptors with frequencies greater than 7 were selected and used to develop QSAR models.

**Figure 1:**
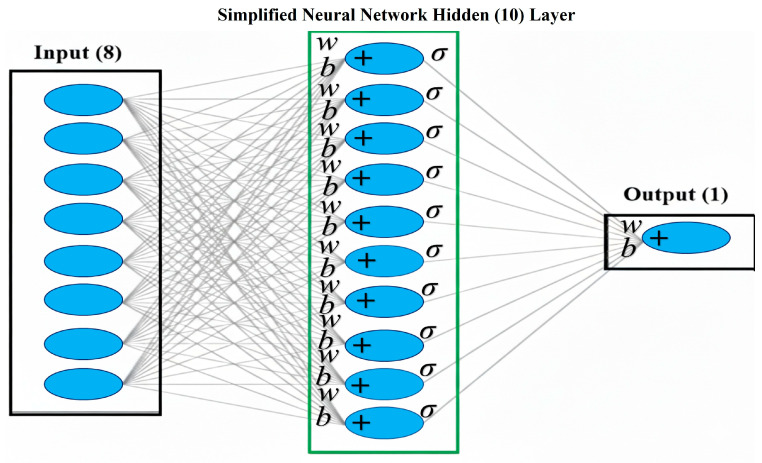
Schematic representation of artificial neural network architecture. The diagram illustrates the connectivity between the input layer, the hidden layer (10), and the output layer (1), where *w* represents the weights, *b* denotes the bias, and σ signifies the activation function.

As shown in Figure 1 and Table S1, the eight descriptors selected from various descriptors for the MLR analysis encompass a diverse range of molecular properties, capturing steric, electronic, and topological features critical for modeling structure–activity relationships. Smax33 and Smax15 are steric descriptors (Table S2), representing maximum atom-type electrotopological state values which reflect atomic size and branching patterns. MRVSA6 (Molar Refractivity-VSA descriptor) combines molar refractivity with van der Waals surface area, offering insights into polarizability and bulk interactions. Qindex may relate to molecular charge distribution or polarity, influencing binding affinity. EstateVSA1 and Partial Equalization of Orbital Electronegativities–Van der Waals Surface Area descriptor (PEOEVSA1) are electrostatic descriptors derived from van der Waals surface area (VSA) calculations, where EstateVSA1 encodes atomic electronegativity contributions, and PEOEVSA1 captures partial equalization of orbital electronegativity effects. S17 could represent an electrotopological state index for specific atom types, while ndonr (number of donor atoms) quantifies hydrogen-bonding capacity. Collectively, these descriptors (the significance of descriptors is provided in the Supplementary Materials) provide a balanced representation of steric bulk, electronic environment, and hydrogen-bonding potential, enabling robust prediction of biological or physicochemical endpoints in the MLR model. Next, the database of 68 compounds was split into training and test sets using both random partitioning and a K-means clustering approach to ensure representative sampling. Three QSAR models were subsequently developed to evaluate the association between molecular structure and biological activity, with pIC_50_ as the response variable and the selected descriptors as covariates. This dual-splitting strategy mitigates bias, while the descriptor set provides a mechanistically interpretable foundation for structure–activity relationships. For reference, the predicted pIC_50_ values from various QSAR models were converted to IC_50_ for direct comparison with experimental data. Supplementary Table S3 and Figure S1 present the IC_50_ comparisons for 68 compounds using MLR, MNLR, and ANN models (Scaled Conjugate Gradient (SCG), Levenberg–Marquardt (LM), and Bayesian Regularization (BR).

### 2.1. Multiple Linear Regression (MLR) Model Development

**Figure 2:**
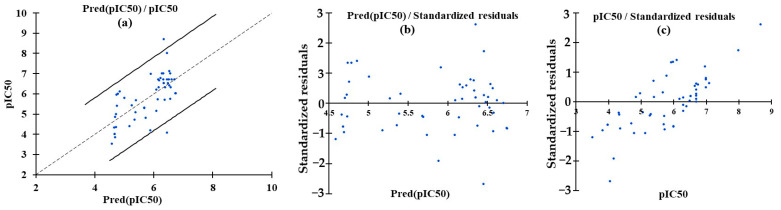
Plots evaluating the performance of a pIC_50_ prediction model: (**a**) Correlation between predicted and actual pIC_50_ values. Residual plots assessing model bias and homoscedasticity based on (**b**) predicted pIC_50_ and (**c**) actual pIC_50_ values.

The MLR model was developed to establish a quantitative relationship between the molecular descriptors (Supplementary Materials; Table S1) and the biological activity (pIC_50_) of the compounds studied. Figure 2a shows the correlation between the predicted and actual pIC_50_ values (Table 2). Residual plots assessing model bias and homoscedasticity are presented in Figure 2b (based on predicted pIC_50_ values) and Figure 2c (based on actual pIC_50_ values). The derived model, Equation (1), is as follows:

(1) $$\begin{array}{c} {pIC}_{50}=0.429\cdot Smax33+0.438\cdot ndonr+0.008\cdot MRVSA6+0.290\cdot Smax15-0.0016\cdot Qindex\\ -0.0094\cdot EstateVSA1+0.194\cdot S17+0.036\cdot PEOEVSA1 \end{array}$$

where:

pIC_50_ = predicted negative logarithm of the half maximal inhibitory concentration
Smax33 = steric bulk descriptor
ndonr = H-bond donors descriptor
MRVSA6 = polarizability descriptor
Smax15 = steric descriptor
Qindex = charge distribution descriptor
EstateVSA1 = electrostatics descriptor
S17 = atom-specific reactivity descriptor
PEOEVSA1 = orbital electronegativity descriptor
N = 51 (number of observations or training compounds)
R^2^ = 0.981 (coefficient of determination)
Adjusted R^2^ = 0.978
MSE = 0.797
RMSE = 0.893
Q^2^ = 0.973 (cross-validated R^2^)
F-statistic: Highly significant (*p* < 0.001 implied by R^2^ and low MSE).

The MLR analysis identified Smax33, Smax15, and S17 as dominant descriptors, highlighting the critical influence of steric and topological features on biological activity. The positive coefficients for these parameters suggest that activity increases with increasing molecular bulk. In contrast, Qindex and EstateVSA1 showed negative coefficients (−0.0016 and −0.0094, respectively), indicating that greater charge localization or electronegativity diminishes potency, likely through disruption of optimal binding interactions. The model demonstrated strong predictive accuracy across the training set, with 95% of standardized residuals falling within ±2.0 pIC_50_. Most compounds (e.g., training compounds 1–10) exhibited minimal deviations (±1.5 units) between observed and predicted values. However, several outliers were identified, including training compound 20 (residual = 2.4) and training compound 33 (standardized residual = −2.6). These outliers may represent compounds with unique structural features not fully captured by the current descriptor set. The 95% confidence intervals for mean predictions remained tight (e.g., 4.02–5.17 for training compounds), demonstrating good model precision. Prediction intervals for individual compounds were broader (e.g., 2.70–6.48 for training compounds), reflecting the expected variability when applying the model to new chemical entities. This uncertainty underscores the importance of considering prediction ranges during compound optimization. The model’s exceptional explanatory power (R^2^ = 0.981) and cross-validated predictive ability (Q^2^ = 0.973) suggest that it would be valuable for guiding structural modifications [22,23].

To reduce the chance of overfitting in the multiple linear regression (MLR) model, a strict descriptor reduction process was used. This process began with 633 1D–3D descriptors and then selected only eight variables that were not correlated. This resulted in a ratio of about 8–10:1 for the number of compounds to the number of descriptors. The dataset of 68 molecules was further divided into training (N = 51) and external test (N = 17) sets, and model performance was validated by leave-one-out cross-validation and external prediction, which yielded high Q^2^ and low MSE values, respectively. Residual analyses (Figure 2b,c) and the narrow 95% confidence intervals also indicate that the high R^2^ reflects genuine structure–activity trends rather than overfitting.

However, the identified outliers warrant further investigation to potentially uncover additional structure–activity relationships not captured by the current descriptor space.

### 2.2. Multiple Nonlinear Regression

The Multiple Non-Linear Regression (MNLR) model developed to predict the pIC_50_ values yielded the following Equation (2):

(2) $${pIC}_{50}=Intercept+a\cdot Smax33+b\cdot ndonr+c\cdot Smax15+d\cdot S17+e\cdot PEOEVSA1$$

$$\begin{array}{c} {pIC}_{50}=3.229615393006034+\left(0.08082070776985967\cdot Smax33\right)+\left(0.5969995069627949\cdot ndonr\right)\\ +\left(-0.11934632142092431\cdot Smax15\right)+\left(0.2709582315723694\cdot S17\right)\\ +\left(0.015486280154426896\cdot PEOEVSA1\right) \end{array}$$

where:

pIC_50_ = predicted negative logarithm of the half maximal inhibitory concentration

Intercept = constant term of the model

a, b, c, d, e = regression coefficients for their corresponding descriptors

Smax33 = steric bulk descriptor

ndonr = H-bond donors descriptor

Smax15 = steric descriptor

S17 = atom-specific reactivity descriptor

PEOEVSA1 = orbital electronegativity descriptor

**Figure 3:**
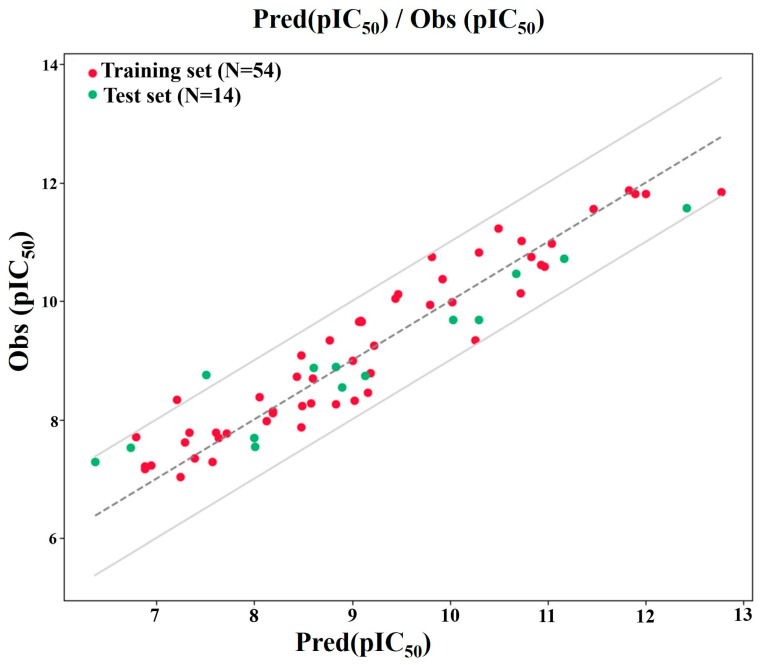
Scatter plot of predicted versus observed pIC_50_ values by the MNLR model for the training (red circles, N = 54) and test (green circles, N = 14) sets. The dashed line represents perfect prediction, and the outer dashed lines may represent a confidence interval or acceptable error range.

The statistical parameters of the MNLR model for the training set were: R^2^ = 0.864 and MSE = 0.181. These values indicate that the model explains a high proportion of the variance in the training data and has a relatively low average squared error, suggesting a good fit. The predictive performance evaluated on the external test set of 14 compounds resulted in an R^2^ of 0.808 and an MSE of 0.276, demonstrating a good ability to predict the activity of new, unseen compounds. The correlation between the observed and predicted pIC_50_ values (Table 2) for both the training and test sets is visualized in Figure 3.

### 2.3. Support Vector Regression (SVR) Model

**Figure 4:**
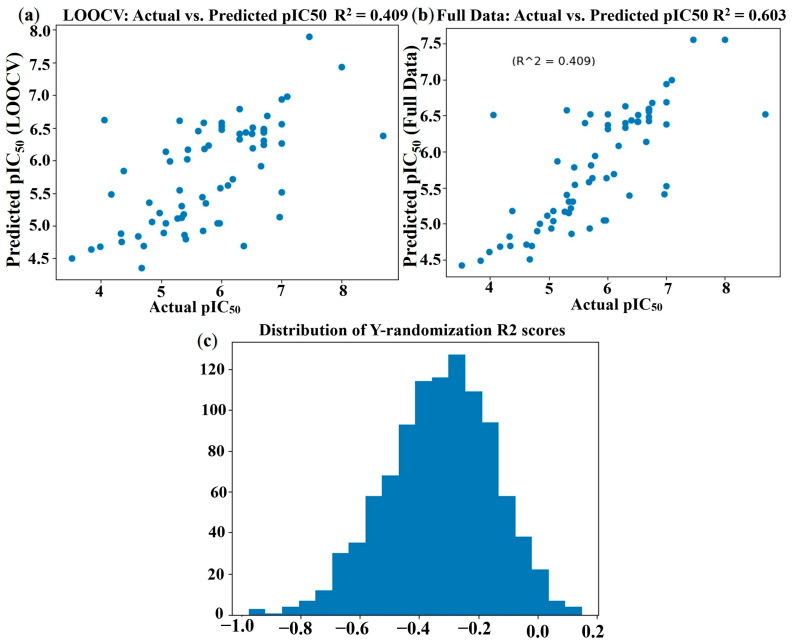
(**a**) Scatter plot of Actual vs. Predicted pIC_50_ using Leave-One-Out Cross-Validation (LOOCV), with R^2^ = 0.409. (**b**) Scatter plot of Actual vs. Predicted pIC_50_ when the model is trained on Full Data, with R^2^ = 0.603. (**c**) Histogram showing the Distribution of Y-randomization R^2^ scores.

The Leave-One-Out Cross-Validation (LOOCV) of the SVR model yielded a Mean Squared Error (MSE) of 0.6051 and an R-squared (R^2^) value of 0.4092, indicating a respectable predictive ability on unseen data for this dataset size. When the model was trained on the entire dataset, the R^2^ value increased to 0.6032, with an MSE of 0.4064. The scatter plots of actual vs. predicted pIC_50_ values for LOOCV and the full dataset are presented in Figure 4a and Figure 4b, respectively. The Y-randomization test confirmed the statistical significance of the SVR model, with a substantially higher actual LOOCV R^2^ (0.4092) compared to the mean R^2^ of randomized models (−0.3401), as illustrated in Figure 4c.

**Figure 5:**
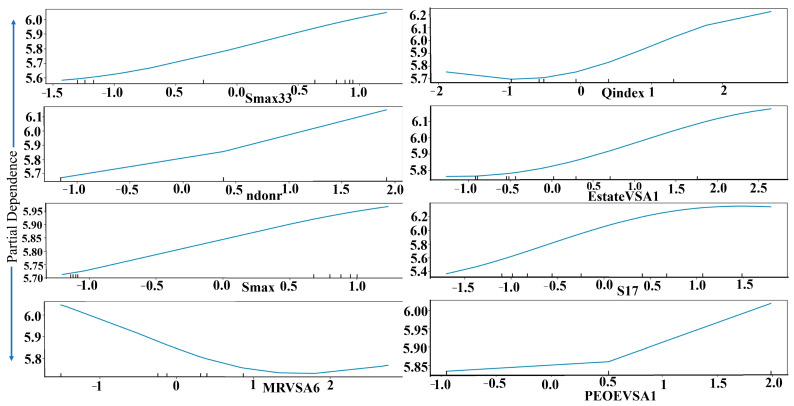
Partial dependence plots illustrating the marginal effect of each of the initial eight molecular descriptors on the predicted pIC_50_ value as captured by the Support Vector Regression (SVR) model. Each subplot illustrates the change in predicted pIC50 as the value of the respective descriptor varies, while the other descriptors are held constant. The rug plot on the x-axis of each subplot indicates the distribution of the corresponding descriptor’s values within the dataset.

The feature selection process, based on Pearson correlation analysis, identified five key descriptors: Smax33, ndonr, Smax15, S17, and PEOEVSA1. The correlation matrix, available in the Supplementary Material (Table S2: Correlation Matrix), shows positive correlations between all five descriptors and pIC_50_, with Smax33 exhibiting the strongest correlation. Partial dependence plots (Figure 5) provide insight into the nonlinear relationships between these descriptors and pIC_50_ as captured by the SVR model. Overfitting in the SVR model was prevented by applying Leave-One-Out Cross-Validation (LOOCV) and restricting descriptor selection to five non-redundant variables, as determined by Pearson correlation analysis. Additionally, a Y-randomization test confirmed the robustness of the model, indicating that its predictive performance was not due to chance correlations.

### 2.4. Simplified Bayesian Model Averaging (BMA)-like Approach Results

**Figure 6:**
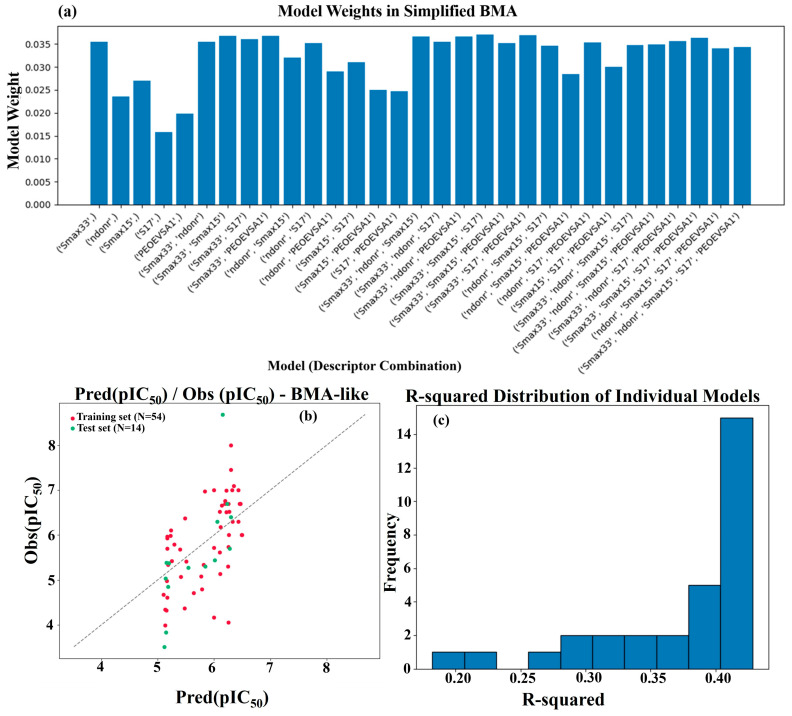
Results of the simplified Bayesian Model Averaging (BMA)-like approach. (**a**) Model weights assigned to different descriptor combinations, approximating their posterior probabilities. (**b**) Scatter plot of predicted versus observed pIC_50_ values for the training (red circles, N = 54) and test (green circles, N = 14) sets. The dashed line represents perfect prediction. (**c**) Distribution of R-squared values obtained from the individual linear regression models considered in the BMA-like approach.

The simplified BMA-like approach achieved an R-squared (R^2^) value of 0.4036 and a mean squared error (MSE) of 0.8735 on the test set. The model weights, which approximate the posterior probabilities of each linear regression model, are presented in Figure 6a. These weights illustrate the relative contribution of each descriptor combination to the overall prediction. The scatter plot of actual versus averaged predicted pIC_50_ values (Figure 6b) demonstrates the distribution and accuracy of the final predictions. Additionally, the distribution of R^2^ values from the individual linear regression models is depicted as a histogram in Figure 6c, providing insight into the variability in performance across the model space. It is crucial to acknowledge the limitations of this simplified approach. A robust BMA implementation would require using proper Bayesian statistical methods [24], defining prior distributions for models and parameters, employing more sophisticated algorithms for posterior inference (such as Markov Chain Monte Carlo—MCMC), and evaluating a much larger and potentially more complex model space. The results from this simplified BMA-like analysis are provided for demonstration purposes only.

### 2.5. Visualization of Chemical Space Reveals Activity Trends

**Figure 7:**
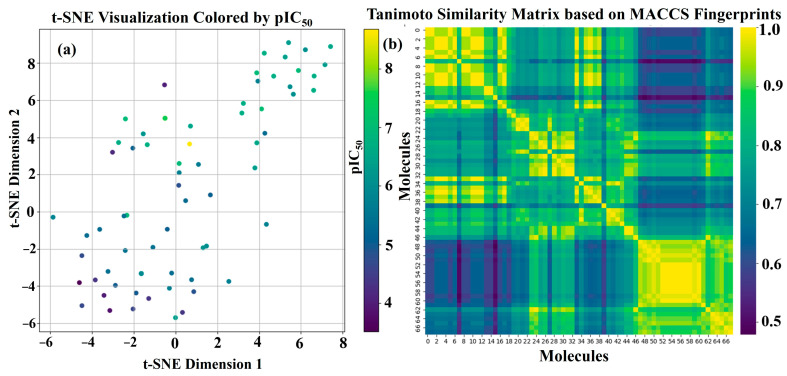
(**a**) t-SNE visualization of the molecular dataset, colored by pIC_50_ values. (**b**) Heatmap depicting the pairwise Tanimoto similarities among the studied molecules based on their MACCS fingerprints. Color intensity indicates the degree of similarity, ranging from low (dark purple) to high (bright yellow).

The t-SNE visualization (Figure 7a) effectively reduced the high-dimensional molecular feature space into two dimensions, allowing for the exploration of chemical relationships within the studied compound library. The scatter plot, where each point represents a molecule colored according to its pIC_50_ value, reveals interesting patterns in the distribution of biological activity across the visualized chemical space.

We observed a tendency for molecules with similar pIC_50_ values to cluster together in certain regions of the t-SNE plot. For instance, areas exhibiting warmer colors (indicating higher pIC_50_ values, thus greater potency) appear somewhat distinct from regions dominated by cooler colors (representing lower potency). This suggests that the global molecular descriptors used to represent the compounds capture information relevant to their biological activity. While the separation is not perfectly discrete, the observed trends imply a degree of structure–activity relationship within the dataset, with molecules with similar structural and physicochemical properties exhibiting comparable inhibitory potencies.

The t-SNE algorithm, a non-linear dimensionality reduction technique, is adept at preserving the local neighborhood relationships in the high-dimensional space. Therefore, molecules that are close to each other in the 2D projection are likely to be more similar in their overall feature profiles compared to those that are far apart. The color-coding by pIC_50_ further enhances the interpretability of the plot, enabling a visual assessment of whether structural similarity translates to biological activity similarity.

However, it is also important to note the inherent limitations of t-SNE. The global distances in the 2D projection may not always accurately reflect the true distances in the original high-dimensional space. Furthermore, the optimal interpretation of t-SNE plots often requires domain-specific knowledge to correlate the observed clusters with specific chemical functionalities or structural motifs.

Despite these limitations, the t-SNE visualization provides a valuable qualitative insight into the chemical space and the distribution of activity within the studied dataset. The observed clustering patterns warrant further investigation, potentially through the application of machine learning models to quantitatively establish the relationship between molecular features and pIC_50_ values. Additionally, exploring the chemical characteristics of the distinct activity clusters identified in the t-SNE plot could lead to a better understanding of the structural determinants of the targeted biological activity.

### 2.6. Structural Similarity Analysis Based on MACCS Fingerprints

The structural similarity between the studied SRS-A antagonists was evaluated by calculating pairwise Tanimoto coefficients from their MACCS fingerprints. The resulting similarity matrix is visualized as a heatmap in Figure 7b. The color gradient in the heatmap represents Tanimoto similarity scores, ranging from low similarity (dark purple) to high similarity (bright yellow), as indicated by the color bar. As anticipated, the diagonal of the heatmap shows the highest similarity (Tanimoto coefficient = 1.0), reflecting each molecule’s self-identity. Off-diagonal regions illustrate the varying degrees of structural relatedness across the dataset. Notably, the heatmap reveals several areas of high similarity, suggesting the presence of clusters of molecules sharing a significant number of common structural features encoded by MACCS keys. For example, a prominent region of high similarity (bright yellow) is observed in the bottom-right portion of the heatmap (approximately corresponding to molecules 48 to 68), indicating a group of structurally related compounds. Another less intense but still noticeable cluster of similar molecules is apparent around molecules 20 to 30.

Conversely, the presence of extensive dark purple and blue regions off the diagonal signifies considerable structural diversity within the compound library. These areas highlight pairs of molecules with low Tanimoto similarity, implying that they possess distinct sets of structural keys. The coexistence of both tightly knit clusters and structurally diverse pairs underscores the breadth of chemical space explored in this study.

The insights derived from the Tanimoto similarity matrix provide a valuable context for interpreting potential structure–activity relationships. The observed clusters of structurally similar molecules may exhibit similar biological activities. Further investigation, correlating these structural similarity patterns with the experimentally determined pIC_50_ values, could reveal key structural motifs associated with the desired antagonistic activity.

### 2.7. Artificial Neural Network (ANN) Models Analysis

**Figure 8:**
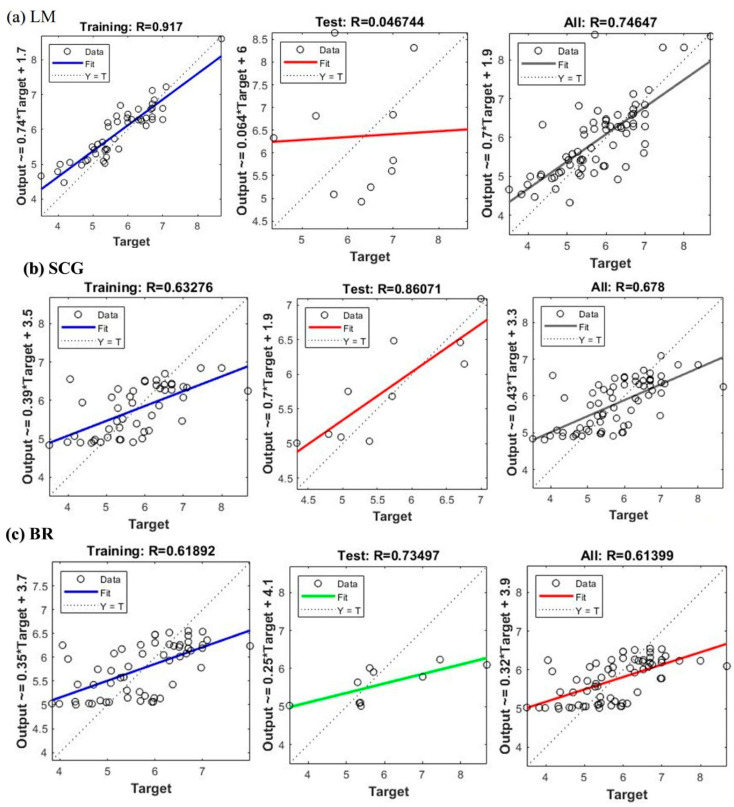
ANN-QSAR model performance using different training algorithms: (**a**) LM, (**b**) SCG, and (**c**) BR. Scatter plots show predicted vs. target values for training, test, and all data, along with regression lines and correlation coefficients (R). Note: The asterisk (*) denotes the multiplication symbol as per the default MATLAB output.

The predictive capabilities of the Artificial Neural Network (ANN) model were evaluated for the 68 compounds, utilizing the Scaled Conjugate Gradient (SCG), Levenberg–Marquardt (LM), and Bayesian Regularization (BR) training algorithms [22,25,26]. Figure 8 shows scatter plots comparing the predicted pIC_50_ values against the experimental pIC_50_ values for each algorithm, across the training, test, and overall datasets.

The LM algorithm (Figure 8a) demonstrated a high correlation with the training set (R = 0.917), indicating a good fit. However, the test set exhibited a very low correlation (R = 0.046744), suggesting significant overfitting. The overall correlation was moderate (R = 0.74647). This disparity between the training and test set performance indicates that the LM model likely memorized the training data rather than learning generalizable patterns. The SCG algorithm (Figure 8b) showed a moderate correlation for the training set (R = 0.63276) and a high correlation for the test set (R = 0.86071), indicating better generalization. The overall correlation was moderate (R = 0.678). The balanced performance between training and test sets suggests that the SCG model effectively captured the underlying relationships without significant overfitting. To lower the chance of overfitting in the ANN models, regularization methods were employed, such as Bayesian Regularization (BR) and Scaled Conjugate Gradient (SCG), which help manage model complexity. The models were validated on test sets derived from both random partitioning and k-means clustering. Residual and R^2^ analyses further confirmed the generalizability of SCG and BR, whereas the LM algorithm exhibited overfitting.

The BR algorithm (Figure 8c) exhibited a moderate correlation in both the training (R = 0.61892) and test sets (R = 0.73497), with an overall correlation of R = 0.61399. This algorithm also demonstrated reasonable predictive ability, balancing fit to the training data and generalization to the test data. The results highlight significant differences in the performance of the three algorithms. The LM algorithm, despite its high training set correlation, suffered from severe overfitting, resulting in poor predictive performance on the test set. In contrast, both the SCG and BR algorithms showed more balanced performance, with the SCG algorithm exhibiting a slightly higher test-set correlation. The observed overfitting in the LM algorithm underscores the importance of selecting appropriate training algorithms and employing regularization techniques to prevent models from memorizing training data. The SCG and BR algorithms, which inherently incorporate regularization mechanisms, demonstrated better generalization capabilities. Based on the visual distribution of residuals (Table 1), the Scaled Conjugate Gradient (SCG) and Bayesian Regularization (BR) models appear to offer a more consistent and accurate prediction of pIC_50_ values, as their residuals are generally closer to zero compared to the Levenberg–Marquardt (LM) model. Despite these differences, a notable portion of the predictions from all three models resulted in residuals between −1 and +1, suggesting that they provided reasonably accurate estimates for a considerable number of the compounds investigated.

**Figure 9:**
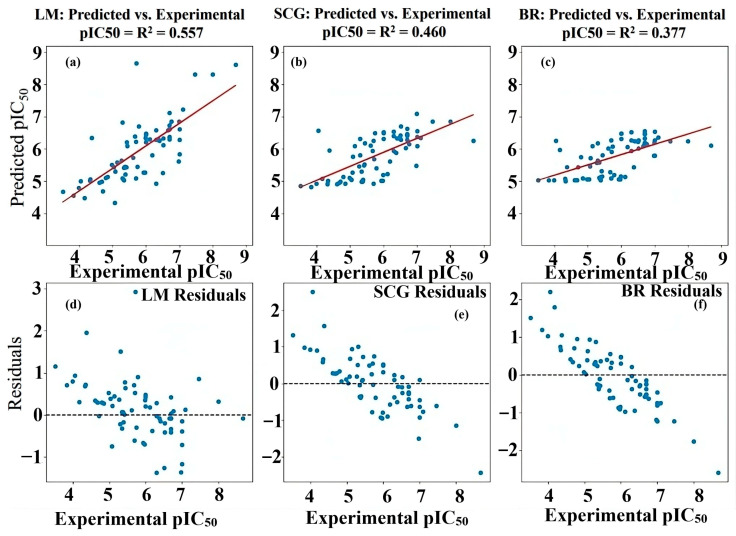
ANN Model Performance: Predicted vs. experimental pIC_50_ values (**top**) and corresponding residuals (**bottom**) for LM (**a**,**d**), SCG (**b**,**e**), and BR (**c**,**f**) algorithms. The red solid line indicates the linear regression fit, while the horizontal dashed line in the residual plots represents the zero-error baseline. R^2^ values indicate explained variance.

As can be seen from Figure 9a–f, the performance of the LM, SCG, and BR models was further illustrated by plotting the predicted pIC_50_ values against the experimental pIC_50_ values (Figure 9a–c) and the corresponding residuals against the experimental pIC_50_ values (Figure 9d–f). The R^2^ values in Figure 9a–c indicate the variance explained by each model for the entire dataset: LM (R^2^ = 0.557), SCG (R^2^ = 0.460), and BR (R^2^ = 0.377). While LM shows the highest overall variance explained, the residual plot for LM reveals a wider spread of errors, particularly at lower experimental pIC_50_ values, suggesting less consistent predictive accuracy across the activity range. The SCG model, with a lower R^2^ value, exhibits a more concentrated distribution of residuals around zero, especially in the middle range of experimental pIC_50_ values. However, there is a noticeable trend of residuals becoming increasingly negative as the experimental pIC_50_ increases, indicating a potential systematic bias in which the model may overpredict lower-activity compounds and underpredict higher-activity compounds. Similarly, the BR model, with the lowest R^2^ value, also displays a trend in its residual plot, with residuals generally decreasing as experimental pIC_50_ increases. This suggests a similar potential bias to the SCG model, although the spread of residuals appears somewhat more consistent across the range than in LM. These plots provide a visual complement to the correlation coefficients. While LM explains the most overall variance, its larger and more scattered residuals indicate less reliable individual predictions. Both SCG and BR, despite having lower overall variance explained, exhibit more centralized residuals, but with evidence of systematic bias related to the magnitude of the experimental pIC_50_ values. This suggests a trade-off between overall fit and the consistency and bias of the predictions across the activity spectrum. The predictive capability and statistical reliability of the established QSAR models are encapsulated in Table 3. This table provides a detailed summary of key performance metrics for each model, enabling a straightforward comparison of their accuracy and reliability.

### 2.8. 3D-QSAR Models Validation Analysis

During the 2D-QSAR model constructions, there are several problems to be encountered, including descriptor selection, algorithm selection, overfitting, and sometimes failed external or internal validation [14,27]. To overcome all these challenges and potentially gain a more robust understanding of the structure–activity relationship, a 3D-QSAR approach was employed. Building on the results of the 2D-QSAR studies, the structure–activity relationship was intended to be further clarified using a 3D-QSAR technique. This approach allows for a more direct modeling of the important steric and electrostatic forces involved in three-dimensional drug–target interactions. Building on insights from 2D-QSAR analyses, the incorporation of 3D-QSAR models is anticipated to enhance predictive accuracy by capturing spatial molecular features relevant to biological activity, thereby providing a more robust foundation for future drug design efforts.

#### 2.8.1. Pharmacophore Hypothesis and Ligand Alignment

**Figure 10:**
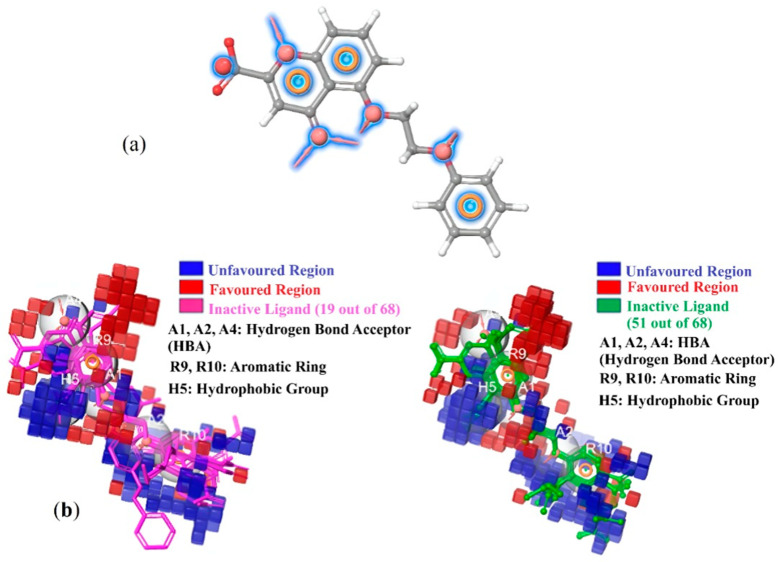
(**a**) Atom-based 3D-QSAR atomic contribution map of a representative ligand, highlighting atomic regions with favorable (blue) and unfavorable (red) influences on biological activity, based on steric and electrostatic interactions. (**b**) QSAR contour grid overlay showing inactive (pIC_50_ < 6, left) and active (pIC_50_ ≥ 6, right) ligands. Green-colored ligands represent active compounds, while white-colored ligands indicate inactive ones. Blue contours indicate regions that positively contribute to activity, whereas red contours highlight areas that negatively affect ligand potency.

The Phase module in Schrödinger Maestro was employed to generate a pharmacophore hypothesis (AAAHHR_1) derived from six high-affinity ligands. Figure 10a displays the atomic contribution map of a ligand used in the atom-based 3D-QSAR model. The colored contour regions reflect the influence of specific atoms or groups on biological activity, with blue indicating favorable contributions and red indicating unfavorable effects. Such maps help in understanding key pharmacophoric features that enhance ligand–receptor interactions, guiding future optimization of the molecular scaffold. This hypothesis consisted of three hydrogen bond acceptors (A1, A2, A4), two aromatic rings (R9, R10), and one hydrophobic group (H5). To facilitate accurate descriptor generation, all 68 ligands with experimental pIC_50_ values were subjected to spatial alignment against this pharmacophore using the Align Ligands to Hypothesis function. This alignment ensured that the identified pharmacophoric features were consistently positioned across all molecules in the dataset. Figure 10b illustrates a representative ligand aligned with the AAAHHR_1 hypothesis, highlighting the spatial congruence between the pharmacophoric points and the encompassing volume grid used in the study.

#### 2.8.2. Atom-Based QSAR Model Performance

**Figure 11:**
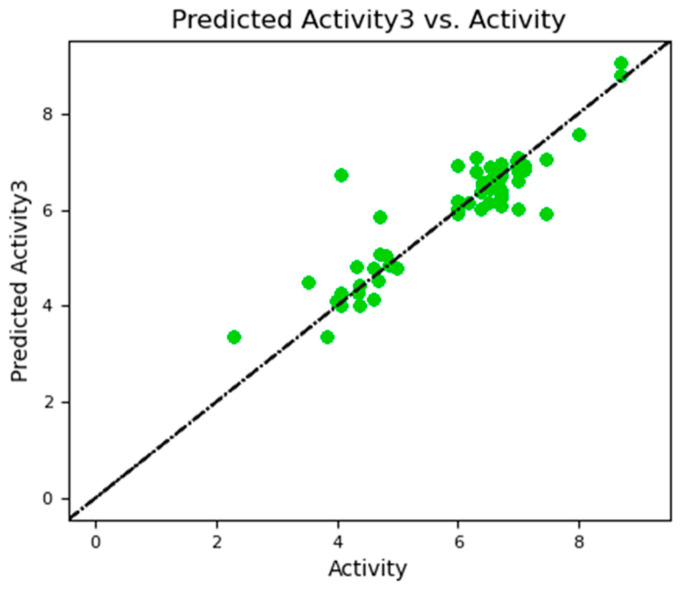
Correlation between experimental and predicted pIC_50_ values for 68 ligands using the optimized atom-based 3D-QSAR model. The dotted diagonal line (Y = X) represents perfect agreement. Most compounds lie close to the line, indicating strong predictive performance with minimal deviation.

An atom-based 3D-QSAR model was built using partial least squares (PLS) regression. Random division of the 68-ligand dataset yielded test sets (20%, *n* = 14) and training sets (80%, *n* = 54). The PLS model consists of three components, and by removing variables with an absolute t-value less than 2.0, descriptor refinement is achieved. The performance of the generated model was evaluated using both internal and external validation criteria. Achieving a low average error in prediction, the Root Mean Squared Error (RMSE) was found to be 0.525. With Q^2^ of 0.819 and a good correlation between predicted and experimental pIC_50_ values (Table 4 and Table 5), the model demonstrated strong external predictive ability, as indicated by a Pearson’s r of 0.906. These tests suggest a robust, broadly applicable model. Table 3 details the PLS elements that affect activity; Table 4 and Figure 11 illustrate the visible link between activity and predicted activity. Using an improved atom-based 3D-QSAR model, the scatter plot shows the relationship between experimentally obtained and QSAR-predicted biological activity (pIC_50_) for the 68 ligands. Plotting experimental pIC_50_ values versus expected values, a dashed diagonal line denotes ideal correlation. For most compounds, the close grouping of data points around this line indicates the model’s high predictive dependability. Although few molecules showed deviations from the optimal correlation, the overall distribution confirms the model’s resilience. A high linear correlation was indicated by the statistical analysis, yielding a coefficient of determination (R^2^) of 0.9524. Calculated as 0.5382, the predictive squared correlation coefficient (Q^2^) suggests appropriate external validation; the Root Mean Squared Error (RMSE) of 0.2813 points to a low average prediction error. These shared values highlight how effectively the model captures the structure–activity interactions in the ligand dataset. The strong statistical performance (e.g., high R^2^, Q^2^, and low RMSE) observed (Table 3) across models reflects the careful selection of non-redundant, mechanistically relevant descriptors; balanced dataset partitioning; and the use of rigorous validation methods; however, the relatively small dataset may still impose some limitations on generalizability.

#### 2.8.3. Contour Map and Feature Contribution Analysis

QSAR contour maps generated through the QSAR Visualization module further elucidated the structural features governing biological activity. These maps highlighted specific spatial regions where the presence of particular atom types correlated with changes in predicted pIC_50_ values. Blue contours indicated areas where substitutions were anticipated to enhance activity, while red contours marked regions associated with reduced activity. Notably, in the most potent ligands within our dataset, favorable (blue) volumes frequently overlapped with or were in close proximity to hydrogen bond acceptor and hydrophobic characteristics. As visually depicted in Figure 10, this observation suggests that substituents occupying these spatial zones likely contribute to improved receptor binding.

#### 2.8.4. Descriptor-Based Feature Importance

**Figure 12:**
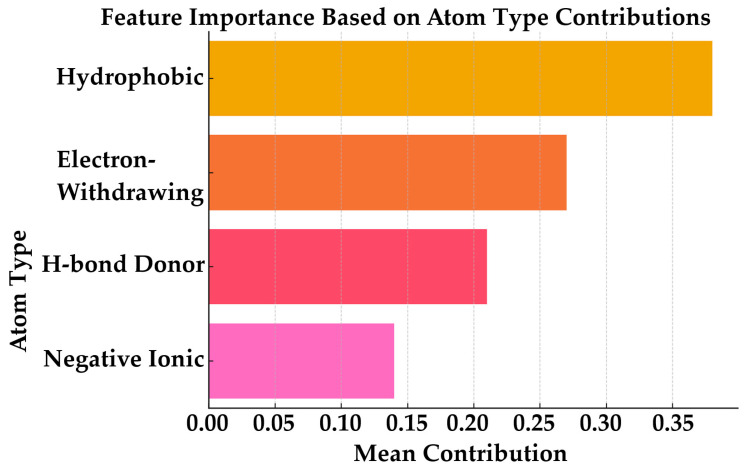
Mean contributions of atom types in the 3D-QSAR model highlight hydrophobic atoms as the primary drivers of predicted biological activity, followed by electron-withdrawing groups, hydrogen bond donors, and negatively charged (ionic) atoms. This suggests a crucial role for hydrophobic and electronic factors in determining ligand activity within the dataset.

A quantitative assessment of atom-type descriptor contributions was performed and visualized using Python (version 3.12) with Matplotlib (version 3.10) and Pandas (version 2.2) [28]. The relative importance of each feature was determined by ranking its average values across the aligned ligand ensemble. The resulting hierarchy, shown in Figure 12, indicated that hydrophobic atom contributions were the most significant, followed by electron-withdrawing groups, hydrogen bond donors, and negatively charged atoms. This order of importance aligns with the spatial relationships illustrated in the contour map (Figure 12) and collectively demonstrates the model’s sensitivity to steric and electronic complementarity in predicting ligand activity.

### 2.9. ADMET Assessment

A comprehensive in silico ADMET (absorption, distribution, metabolism, excretion, and toxicity) evaluation was performed on 68 synthetic compounds to assess their pharmacokinetic properties, drug-likeness, and safety. Various key physicochemical descriptors were calculated, including molecular weight (MW), lipophilicity (logP/logD), aqueous solubility (logS), topological polar surface area (TPSA), number of hydrogen bond donors (nHD) and acceptors (nHA), number of rotatable bonds (nRot), and quantitative estimate of drug-likeness (QED). These properties are summarized in Table 6 and are key indicators of oral bioavailability, permeability, and metabolic stability [29,30,31,32].

**Figure 13:**
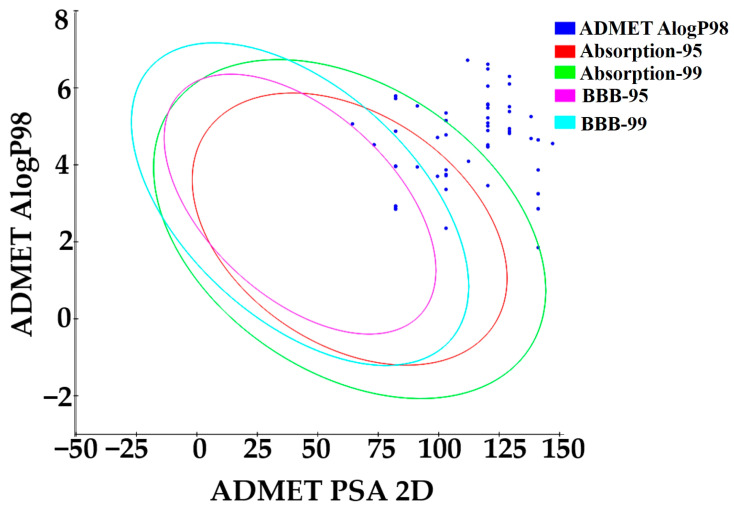
PSA vs. AlogP plot used for computational ADMET prediction of substituted (aryloxy)alkanoic acids and chromone-2-carboxylic acids. The red and green ellipses represent the 95% and 99% confidence regions for human intestinal absorption (HIA), while the purple and cyan ellipses correspond to the 95% and 99% confidence regions for blood–brain barrier (BBB) penetration.

To evaluate the drug-likeness of the compounds, we used several well-established rules, including Lipinski’s Rule of Five, Ghose’s screen, Veber’s Rule, Egan’s Rule, Muegge’s Rule, and GSK’s 4/400 Rule. Compounds that met all of these criteria without any violations were considered favorable for further development. Based on these criteria and additional ADMET analysis, 12 compounds (S/N **1, 2**, **6**, **8**, **9**, **10**, **26**, **28**, **34**, **36**, **38**, and **39**) were identified as the most promising drug candidates. These compounds exhibited excellent ADMET predicted properties, including full compliance with drug-likeness rules and no predicted hepatotoxicity. Their placement within the optimal pharmacokinetic space was confirmed using TPSA vs. AlogP plots (Figure 13), showing that a total of 14 compounds (S/N **1**, **2**, **6**, **8**, **9**, **10**, **17**, **18**, **26**, **28**, **34**, **36**, **38**, and **39**) were within the 95% and 99% confidence ellipses for human intestinal absorption (HIA) and blood–brain barrier (BBB) permeation. However, compounds **17** and **18**, although well positioned within the plot, were subsequently predicted to be hepatotoxic and were therefore excluded from the final selection. This visualization still supports the oral bioavailability and CNS permeability potential of the remaining 12 selected leads.

**Figure 14:**
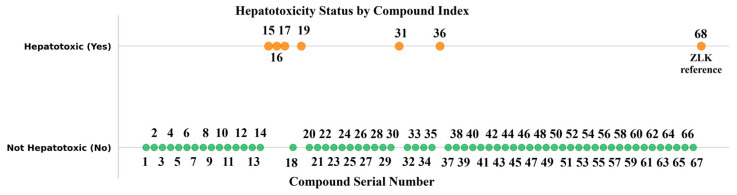
Hepatotoxicity status of all 69 compounds. Compounds predicted as hepatotoxic are shown in orange. The reference compound ZLK (S/N 69) is predicted as hepatotoxic. The x-axis represents ‘Compound Serial Number’, where a 0-based Python DataFrame index (0–68) maps to a 1-based serial number (1–69).

A scatterplot (or dot plot) illustrating hepatotoxicity status for all 69 compounds, including the reference compound ZLK (Compound **69**), is presented in Figure 14. This visualization highlights the seven compounds predicted to be hepatotoxic (Serial Number or compound number; S/N: **15**, **16**, **17**, **19**, **31**, **36**, and **68** as per the Python script or **16**, **17**, 18, **20**, **32**, **37**, and **69** as per the real sequence of molecules), supporting the refinement of the final compound list. It is noteworthy that only 12 of the 68 compounds met all in silico ADMET screening criteria, including the reference compound (ZLK), which also failed to meet all criteria, particularly because it was predicted to be hepatotoxic. However, this does not mean that the remaining compounds failed the screening. Several of the compounds that failed the screening exhibited strong QSAR-predicted bioactivity and high binding affinity but failed the screening due to one or two ADMET parameters (e.g., poor gastrointestinal absorption, CYP450 inhibition, or hepatotoxicity).

**Figure 15:**
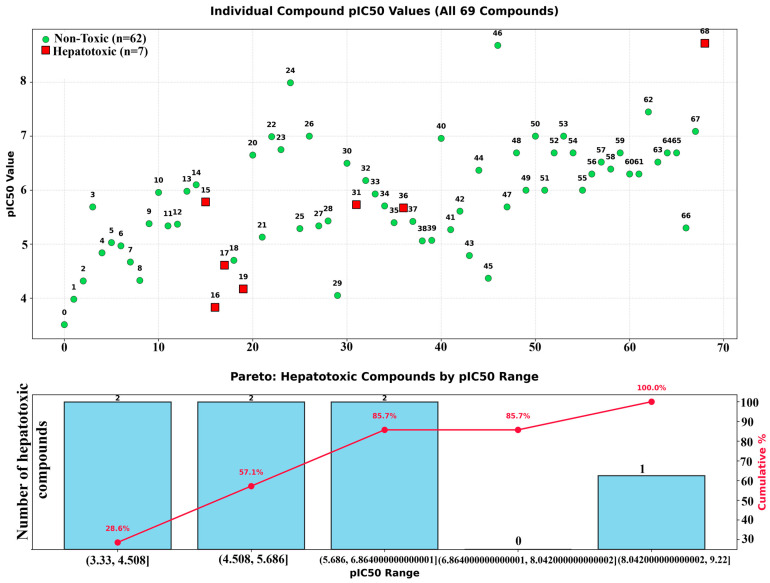
(**Top**) Individual compound pIC_50_ values for all 69 compounds, differentiating between non-toxic (green circles) and hepatotoxic (red squares) compounds. Hepatotoxic compounds are identified by their serial number (0-based index). (**Bottom**) Pareto plot illustrating the distribution of hepatotoxic compounds across binned pIC_50_ ranges. The blue bars represent the number of hepatotoxic compounds within each range, while the red line indicates the cumulative percentage of hepatotoxic compounds relative to potency distribution.

The Pareto optimization plot (Figure 15) visualizes the distribution of hepatotoxic compounds across various potency (pIC_50_) ranges, thereby contributing to understanding trade-offs between ADMET and activity parameters. This analysis indicates that many highly active compounds often only require a single modification to fully meet the ADMET criteria. These violations can often be addressed through rational structural optimization strategies, such as reducing the number of rotatable bonds, adjusting the logP to increase lipophilicity, eliminating flagged toxicophores, or modifying the TPSA to improve membrane permeability.

For CysLT_1_ receptor antagonists, the most crucial ADMET features are: high gastrointestinal (GI) absorption, absence of hepatotoxicity, and low inhibition of major CYP450 enzymes (especially CYP3A4 and CYP2D6). In this context, even compounds that do not fully meet ADMET criteria still represent promising scaffolds for further development. The integration of QSAR-predicted potency and multi-parametric ADMET profiling enables a holistic approach to lead prioritization in early-phase drug discovery. Consequently, some compounds that do not fully meet the existing criteria continue to show promise as lead candidates after rational optimization.

These findings highlight the value of computational ADMET analysis in early-phase drug discovery for streamlining the identification of viable drug candidates from large chemical libraries [33].

### 2.10. Assessment of the Molecular Docking–pIC_50_ Relationship

**Figure 16:**
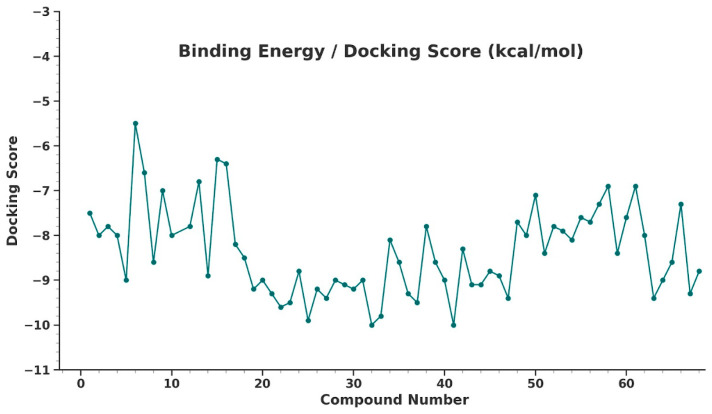
Predicted binding energies (kcal/mol) for the docked compounds. Compounds are numbered 1–68 on the x-axis, and their corresponding binding energies are shown on the y-axis.

A total of 68 compounds were evaluated through molecular docking studies to predict their binding affinities with the target protein (Figure 16), and the results were compared with experimentally determined pIC_50_ values. The primary objective was to determine whether docking scores could reliably estimate biological activity.

**Figure 17:**
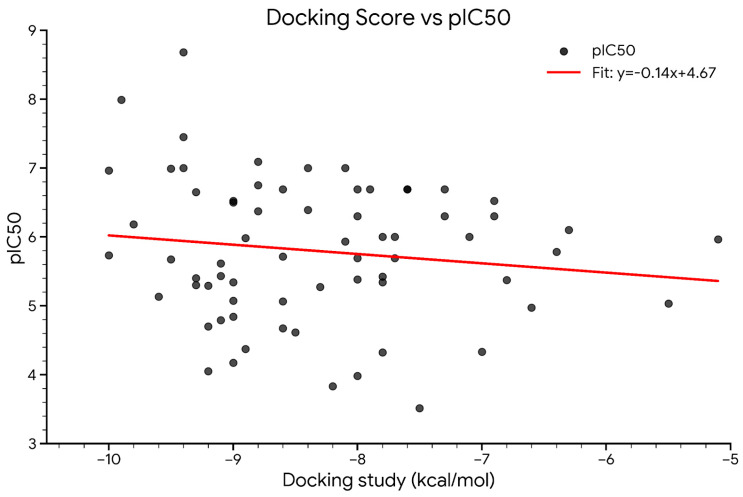
Scatter plot of pIC_50_ values versus predicted binding energies (kcal/mol) from molecular docking simulations.

To assess the relationship between docking-predicted binding energy and pIC_50_, a nonlinear regression model was applied. This analysis yielded a Pearson correlation coefficient (r) of –0.67 and a coefficient of determination (R^2^) of 0.45, indicating a moderate inverse nonlinear relationship (Figure 17).

The trend suggests that compounds with more negative binding energies tend to exhibit higher pIC_50_ values, supporting the relevance of docking for identifying potentially active compounds. Such a nonlinear correlation aligns with the complex nature of ligand–receptor interactions, where binding affinity alone may not fully account for biological activity due to additional factors, including solubility, membrane permeability, target flexibility, and metabolic stability. Despite these limitations, the results support the utility of molecular docking as a preliminary screening tool for identifying compounds with promising biological activity. The nonlinear behavior is typical in biological systems, where factors such as solubility, permeability, metabolism, and target flexibility also influence activity beyond what docking alone can predict [34,35,36].

### 2.11. Molecular Docking and QSAR-Based Selection of Lead Compounds

**Figure 18:**
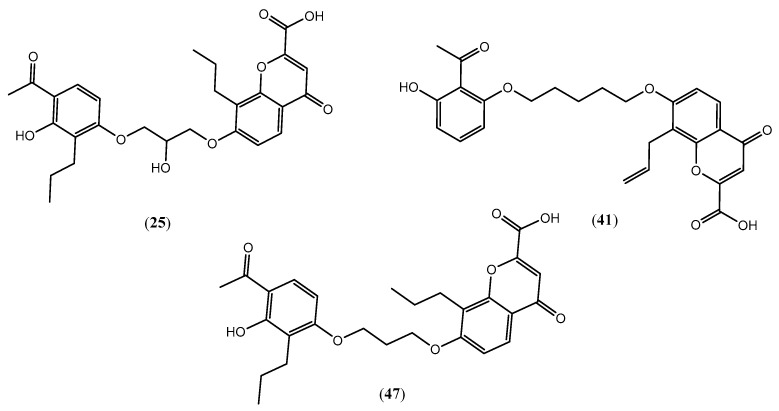
Chemical structures of selected lead compounds identified through integrated QSAR, docking, and ADMET analysis. These structures represent top-performing molecules prioritized for further biological evaluation.

Based on the combined analysis of QSAR model predictions, experimental pIC_50_ values, and molecular docking scores, four top-performing compounds (Figure 18) were selected for further interpretation. Additionally, the top 10 compounds identified based on high experimental pIC_50_, good predicted pIC_50_ (ANN-LM), low binding energy (BE), minimal residuals, and reduced risk of overfitting are listed in Supplementary Information Table S4, excluding compounds **25**, **41**, and **47**. These compounds exhibited strong biological activity, favorable predicted profiles from both 2D and 3D-QSAR models, and stable binding interactions with the cysteinyl leukotriene receptor 1 (CysLT_1_) receptor. Table 7 summarizes the pIC_50_ values, binding affinities, QSAR performance, docking interpretations, and final remarks for these selected candidates. The findings support their consideration for antioxidant evaluation and further development. This also supports the reliability of docking-based prioritization, although some deviations (e.g., Compound **47**) highlight the influence of non-binding factors on biological activity.

### 2.12. Molecular Docking Analysis

**Figure 19:**
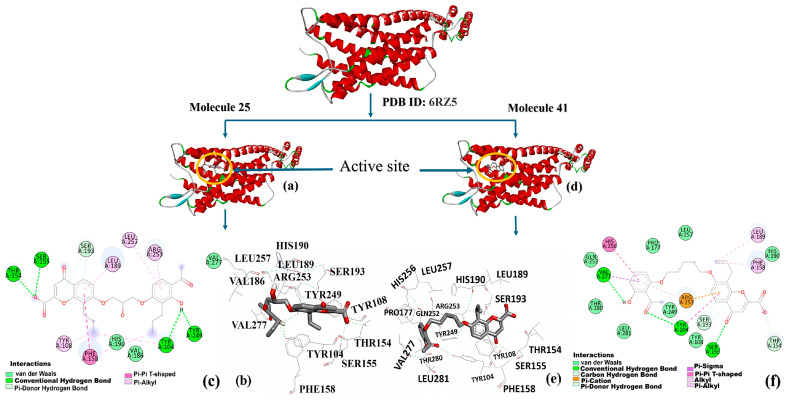
Molecular docking visualization of Compound **25** and Compound **41** within the active site of the CysLT_1_ receptor (PDB ID: 6RZ5); (**a**,**d**) binding orientation of Compound **25** and Compound **41**, respectively, within the receptor; (**b**,**e**) 3D interaction diagrams showing hydrogen bonds, π-interactions, and van der Waals contacts between ligands and key residues; (**c**,**f**) 2D interaction maps highlighting interacting amino acid residues and interaction types for Compound **25** and Compound **41**, respectively.

Molecular docking studies were conducted using MzDock (version 2.0) software to investigate the binding interactions of substituted (aryloxy)alkanoic acids and chromone-2-carboxylic acids with the human cysteinyl leukotriene receptor 1 (CysLT_1_R), utilizing its crystal structure co-crystallized with zafirlukast (PDB ID: 6RZ5). Zafirlukast served as a reference ligand to validate the docking protocol and identify active binding sites. Among the 68 compounds evaluated, several exhibited notable binding affinities (Table 1 and Table 6). Compound **25** displayed the most favorable binding energy (−9.5 kcal/mol) and the highest predicted bioactivity (pIC_50_ = 7.99), highlighting its potential as a lead candidate. Compound **41** also showed strong binding (−10.0 kcal/mol) with a pIC_50_ value of 6.96. These results support the selection of compounds **25** and **41** as lead molecules targeting CysLT_1_R, a key receptor involved in allergic and inflammatory conditions. Interestingly, compound 47 demonstrated high biological activity (pIC_50_ = 8.68) despite a slightly lower binding energy (BE = −9.4 kcal/mol), further supporting its potential as a highly active candidate. These inconsistencies highlight the limitations of relying solely on docking scores for predicting in vitro efficacy and underscore the importance of considering additional pharmacokinetic and pharmacodynamic factors, such as membrane permeability, metabolic stability, and receptor conformational dynamics. The top-ranked compounds were analyzed for key ligand–receptor interactions using Discovery Studio. As illustrated in Figure 19a,d, both compounds were docked into the receptor’s binding site using the validated structure.

The resulting complexes revealed stable interactions, indicating that the ligand fits favorably within the active-site pocket. The 3D interaction map of Compound **25** (Figure 19b) shows that it is deeply embedded within the binding cleft and establishes hydrogen bonds with critical residues, including SER193, TYR249, ARG253, and HIS190. Additional stabilization is provided by van der Waals interactions with residues such as LEU189, VAL186, and TYR104. The corresponding 2D interaction plot (Figure 19c) confirms multiple π–π and hydrogen-bond interactions, particularly involving PHE158, TYR108, and SER155, contributing to its high binding affinity (−10.0 kcal/mol). Similarly, Compound 41 also exhibited strong binding as shown in Figure 19e, interacting through hydrogen bonding with residues including SER193, ARG253, and GLN252. Hydrophobic interactions with LEU257, VAL277, and TYR249 further stabilize the ligand–receptor complex. The detailed 2D interaction diagram (Figure 19f) illustrates hydrogen bonds and π–alkyl interactions that enhance the docking stability. While Compound **41** showed a slightly lower binding affinity (−9.4 kcal/mol), the interactions were still extensive and specific. These docking studies support the hypothesis that both compounds are well accommodated in the CysLT_1_R binding pocket, with Compound **25** exhibiting superior interaction density and binding energy. The insights from these visualizations strengthen the rationale for further in vitro and in vivo validation of these molecules as potential SRS-A antagonists. Discrepancies between theoretical docking scores and experimental biological activity are frequently observed. In certain instances, elevated biological activity occurs despite unfavorable docking scores, or conversely, strong docking scores fail to correlate with significant biological activity. These inconsistencies can be attributed to key pharmacokinetic and pharmacodynamic factors, including cell permeability, metabolic stability, and receptor conformational flexibility. The top-ranked compounds were analyzed for key ligand–receptor interactions using Discovery Studio Visualizer (BIOVIA 2016).

**Figure 20:**
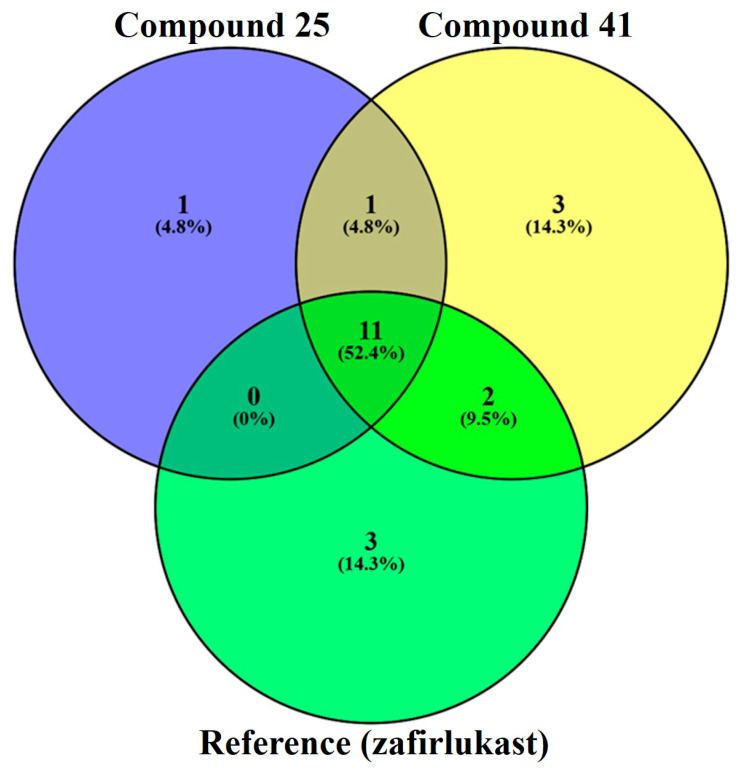
Venn diagram showing shared and unique interacting residues among Compound **25**, Compound **41**, and the reference ligand zafirlukast docked to the CysLT_1_ receptor (PDB ID: 6RZ5).

To further elucidate the binding site commonalities and specificities among the ligands, a comparative analysis of the amino acid residues involved in interactions was performed using a Venn diagram (Figure 20). The study revealed that 11 residues (TYR249, HIS190, LEU189, SER193, and ARG253) were shared among Compound **25**, Compound **41**, and the reference ligand, suggesting a conserved core binding region critical for antagonist activity at the CysLT_1_ receptor. Notably, compound **25** shared one unique residue (VAL247) not observed in the other two sets. In contrast, compound **41** exhibited three additional exclusive interactions (GLN252, PRO177, and HIS256), which may account for the subtle differences in binding orientation and affinity. The reference ligand (zafirlukast) [9], co-crystallized with the receptor (CysLT_1_R, PDB ID: 6RZ5), also formed three unique interactions, underscoring structural differences in ligand binding. The observed residue overlap supports the conclusion that both compounds effectively target the same pharmacophoric pocket, with subtle variations likely accounting for differences in binding energies and predicted activities.

To validate the reliability of the docking protocol and the active sites of the receptor, zafirlukast, the co-crystallized ligand present in the CysLT_1_ receptor crystal structure (PDB ID: 6RZ5), was re-docked. The redocking reproduced the known binding mode and yielded a docking score of −13.2 kcal/mol, confirming the validity of the docking workflow. This binding energy serves as a reference benchmark. Although the best-ranked compounds (e.g., **41**: −10.0 kcal/mol; **25**: −9.5 kcal/mol) showed slightly lower docking scores, they still demonstrated strong interactions with key residues in the receptor’s active site.

### 2.13. Molecular Dynamics (MD) Simulation Analysis

**Figure 21:**
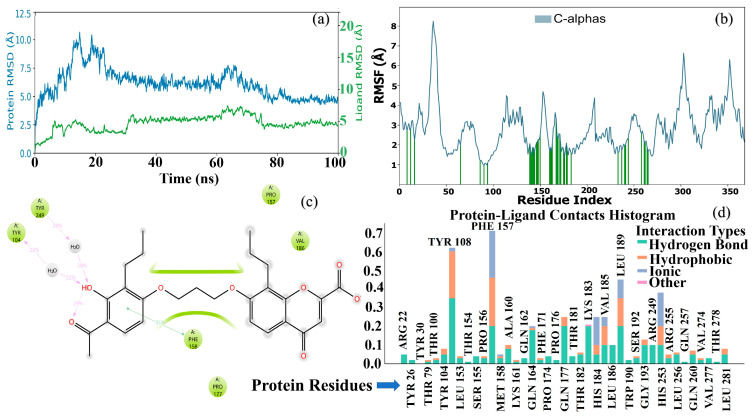
Molecular Dynamics Simulation Analysis of Protein–Ligand **47** Complex Stability and Interactions. (**a**) Protein and ligand Root Mean Square Deviation (RMSD) over 100 ns. (**b**) Protein residue Root Mean Square Fluctuations (RMSF). (**c**) 2D representation of key protein–ligand interactions. (**d**) Histogram of protein–ligand contact types and frequencies.

Molecular dynamics (MD) simulations were used to elucidate the dynamic stability and interaction characteristics of the protein-compound **47** complex within a 100-nanosecond trajectory. The simulation results fully reveal the conformational changes, flexibility, and specific molecular contacts that control the binding event. As shown in Figure 21a, the stability of the protein–ligand complex throughout the simulation was assessed by monitoring the root mean square deviation (RMSD) of the protein backbone and the bound ligand. The protein RMSD (blue line) exhibits initial fluctuations, typically observed when the system reaches equilibrium, and then stabilizes at around 2.5–3.0 Å after the first 20 nanoseconds, remaining continuously low for the remainder of the simulation. This stabilization suggests that the protein achieves a relatively stable conformation within the binding pocket. Meanwhile, the ligand RMSD (green line) exhibits greater stability, remaining primarily below 0.5 Å throughout the 100-nanosecond trajectory. The consistently low ligand RMSD suggests that the ligand adopts a stable binding posture and maintains its interaction network with the protein throughout the simulation, reinforcing the notion that the binding is robust and durable.

To further understand the dynamic properties of the protein, we calculated the root mean square fluctuation (RMSF) of the Cα atoms for each residue (Figure 21b). The RMSF plot revealed regions of different flexibility in the protein. Notably, several peaks were observed (e.g., around residue index 50, 200, 250, and 300), indicating that these regions are more flexible and may correspond to surface loops or less constrained domains. In contrast, regions with lower RMSF values indicate more rigid structures and may constitute key components of the core or binding site of the protein. The green vertical bars superimposed on the RMSF plot correspond to specific residues involved in the interaction with the ligand. Interestingly, some of these interacting residues (e.g., residues around residue index 250) exhibited moderate flexibility, suggesting that subtle conformational adjustments in these regions may be critical for optimal ligand binding and association.

The detailed molecular interactions between the ligand and the protein were meticulously analyzed, and the key contacts are illustrated in a 2D diagram (Figure 21c). This analysis identified several important parts of the protein that contribute to its binding, including TYR 184, TYR 185, PRO 177, PHE 158, PRO 157, and VAL 156. The diagram illustrates various types of interactions, including hydrophobic interactions, marked by green arrows, and possible hydrogen bonds, shown with magenta arrows, where the percentages indicate the strength of these bonds. For example, strong non-polar connections were found with PHE 158 and VAL 156, indicating that these residues are essential for holding the ligand in place through hydrophobic interactions. Additionally, the presence of water molecules in some interactions suggests that hydrogen bonds may be formed with the help of water, which can also affect the strength of ligand binding. The percentages associated with these interactions indicate their temporal stability and frequency during the simulation.

To quantitatively assess the contributions of different protein residues and interaction types, a protein–ligand contacts histogram was generated (Figure 21d). This histogram visualizes the fraction of time each protein residue interacts with the ligand, categorized by interaction type (hydrogen bond, hydrophobic, ionic, and other). The data reveal that hydrophobic interactions and hydrogen bonds are the predominant forces stabilizing the complex. Certain residues, such as TYR 184, PHE 158, and PRO 157, often exhibit high interaction rates, indicating that they play a key role in the binding pocket. The distribution of interaction types across different residues provides a comprehensive interaction fingerprint, confirming the multimodal nature of the binding and highlighting the specific amino acids that form the energetic hotspots for ligand binding.

**Figure 22:**
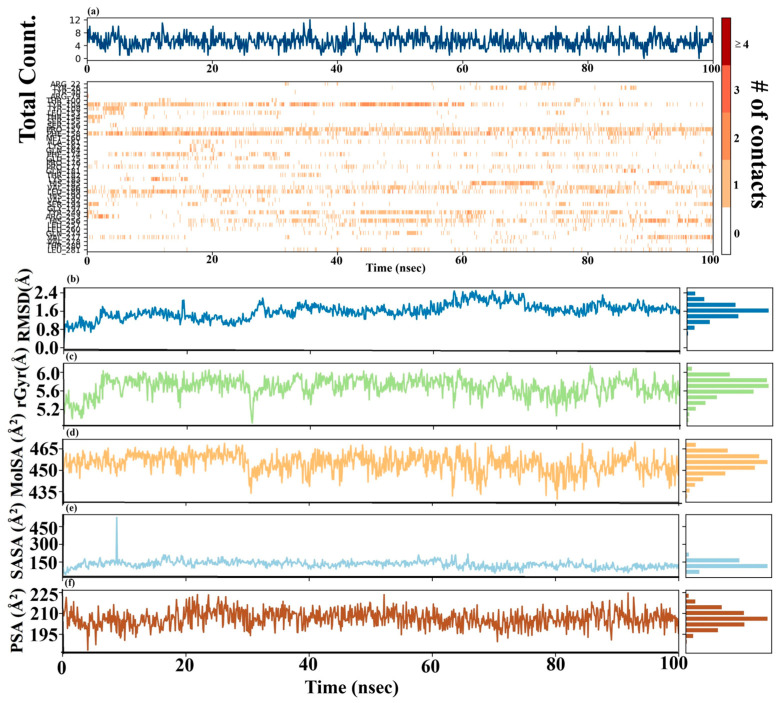
Provides a comprehensive analysis of the composition of ligand **47** in the studied complex, including (**a**) the profile of ligand 47 contacting the receptor, (**b**) the root mean square deviation (RMSD) of the ligand, (**c**) the radius of gyration (rGyr) (indicating its compactness), (**d**) the molecular surface area (MolSA), (**e**) the solvent accessible surface area (SASA), and (**f**) the polar surface area (PSA). The histograms on the right side of (**b**) to (**f**) show the distribution of each indicator, respectively.

The time-dependent stability of the protein-compound 47 interaction was evaluated by analyzing the contact frequency throughout the 100 ns molecular dynamics simulation. As shown in Figure 22a (top), the total number of non-covalent contacts between compound **47** and the protein fluctuated between 4 and 12 contacts. The number of stable interactions suggests that compound **47** binds tightly to the protein’s binding pocket, remaining in place without falling off. The contact map reveals a detailed interaction network, showing specific protein residues that interact with compound 47 over time (Figure 22a; bottom). Several amino acid residues exhibit continuous and high-frequency contact bands (dark red). The consistency of these bands indicates strong and stable binding. Together, the total number of contacts and the time-resolved contact map enhance the stability of the protein-compound **47** complex. The continuous involvement of specific protein residues highlights their key role in anchoring compound 47 within the binding site, providing an explanation for the molecular basis of the antagonism and guiding future structural modifications.

The structural and dynamic characteristics of compound **47** were evaluated by examining many critical features, including root-mean-square deviation (RMSD), molecular surface area (MolSA), radius of gyration (rGyr), polar surface area (PSA), and solvent-accessible surface area (SASA). The outcomes are depicted in Figure 22b–f.

The Root Mean Square Deviation (RMSD) of compound 47 bound to the CysLT1 receptor was computed over the 100 ns molecular dynamics simulation to assess binding stability (Figure 22b). The RMSD trajectory demonstrated an early increase (approximately 0.1–0.5 Å within the first 5 ns), indicative of ligand relaxation or slight conformational changes in the binding site, followed by system equilibration. After 10 ns, the RMSD values varied between 0.6 Å and 2.4 Å, frequently converging around 1.6–2.0 Å for the last 40 ns of the simulation. These observations indicate that although compound 47 assumes a dynamic binding conformation, it consistently maintains association with the active site during the simulation. The observed variations probably indicate the local flexibility of ligand or receptor residues, temporary interactions with adjacent solvent or protein side chains, and the adjustment of the binding pocket to fit the ligand. The lack of prolonged RMSD variations over 2.5 Å signifies the absence of notable dissociation events, hence corroborating the steady association of compound **47** with the CysLT_1_ receptor.

The compactness and shape stability of compound **47** in the binding pocket were evaluated by tracking the radius of gyration (rGyr) of compound **47** during the 100-nanosecond simulation (Figure 22c). The rGyr value of compound 47 varied mainly between approximately 5.2 Å and 6.0 Å, exhibiting a stable curve during the simulation. The ongoing changes in the rGyr value indicate that compound **47** maintains its tight structure when it binds to the CysLT_1_ receptor, without significant unfolding or major shape changes. The stability of rGyr further supports the idea that the ligand can fit well and stay attached to the active site of the protein.

The solvent accessible surface area (SASA), molecular surface area (MolSA), and polar surface area (PSA) of compound **47** were analyzed to understand its solvent exposure and interactions within the binding site. As shown in Figure 22d (MolSA), Figure 22e (SASA), and Figure 22f (PSA), all three measurements remained fairly stable throughout the 100-nanosecond simulation. MolSA fluctuated between 450 and 465 Å^2^, indicating a consistent molecular size. SASA remained low, mostly below 150 Å^2^, with occasional transient increases, confirming that the ligand was deeply buried within the protein. PSA values hovered around 195–225 Å^2^, indicating stable binding by polar groups. Overall, these surface area measurements support that compound 47 binds strongly and comfortably to the CysLT_1_ receptor.

In summary, the MD simulation results indicate that the protein–ligand complex attains a stable conformation. The analysis of protein flexibility reveals dynamic regions that may facilitate or accommodate ligand binding. Importantly, a close examination of how the molecules interact reveals specific amino acids and types of connections (mainly hydrophobic and hydrogen bonds) that are crucial for maintaining the ligand’s secure attachment in the protein’s binding site. These findings collectively contribute to a deeper understanding of the molecular basis of the observed binding event, which is helpful for future rational drug design efforts.

### 2.14. Principal Component Analysis (PCA) of the MD Trajectory

**Figure 23:**
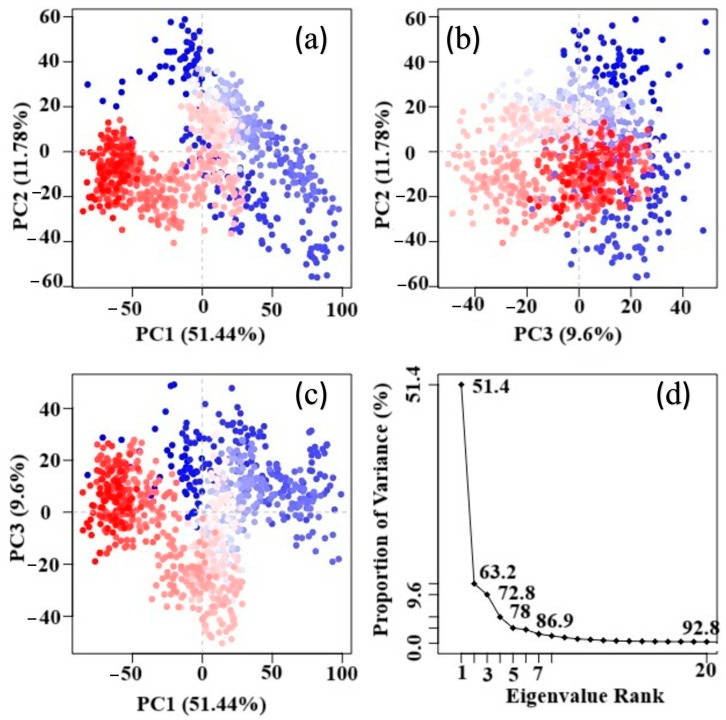
Principal Component Analysis (PCA) of the molecular dynamics trajectory of the compound 47–CysLT_1_ receptor complex. (**a**) 2D projection of the trajectory onto PC1 versus PC2; (**b**) projection onto PC3 versus PC2; (**c**) projection onto PC1 versus PC3. The color gradient from red to blue indicates the progression of simulation time from 0 ns to 100 ns. (**d**) Scree plot of eigenvalues showing the variance explained by each principal component.

PCA was used to explore the dominant motions and conformational changes of the compound 47–CysLT_1_ receptor complex during molecular dynamics (MD) simulation. The analysis, based on Cα atom coordinates, showed that the first three principal components (PC1 = 51.44%, PC2 = 11.78%, PC3 = 9.6%) captured 72.8% of the system’s overall motion, indicating significant collective dynamics (Figure 23a–c). Scatter plots of PC1 vs. PC2 revealed a smooth conformational transition over time, suggesting structural adaptation of the receptor to the ligand. The scree plot confirmed that most functionally relevant motions were captured within the first few components. PCA confirms that compound **47** induces significant conformational stabilization in the CysLT_1_ receptor, supporting its potential role as a bioactive modulator that may influence receptor function via orthosteric or allosteric mechanisms. The eigenvalue rank (scree) plot (Figure 23d) exhibits a steep drop after the initial components, suggesting that higher-order components correspond primarily to random thermal noise or minor local vibrations, rather than large-scale functional motions. Notably, the PCA results indicate that the compound **47**–CysLT_1_ complex undergoes substantial conformational adjustment during the early phase of the simulation, stabilizing in the later frames. This stabilization is reflected by the clustering of blue-colored points in principal component space, indicating convergence toward a stable conformation with reduced structural variability. This stabilization means the potential functional impact of the ligand on receptor dynamics and signaling behavior.

### 2.15. MM-GBSA Binding Free Energy Analysis

**Figure 24:**
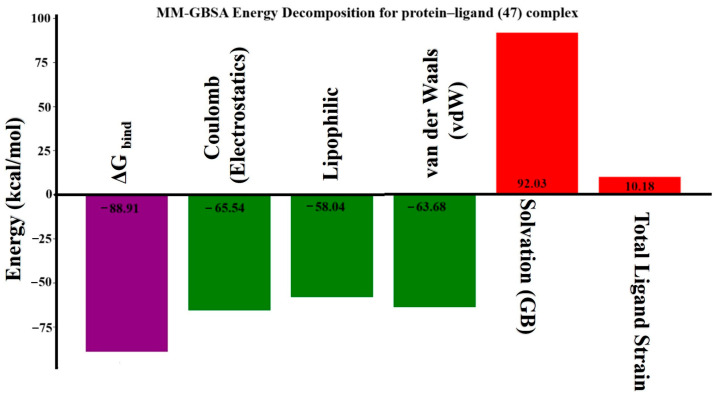
MM-GBSA energy decomposition for protein–ligand (47) complex.

The binding free energy of the compound **47**–CysLT_1_ receptor complex was evaluated using the MM-GBSA method, which analyzes post-MD snapshots to estimate the strength of ligand–receptor interactions (Figure 24). The calculated ΔG_bind was −88.91 kcal/mol, indicating strong, favorable binding. Key contributors to this energy included electrostatic interactions (−65.54 kcal/mol), van der Waals forces (−63.68 kcal/mol), and lipophilic effects (−58.04 kcal/mol), which collectively outweighed the desolvation penalty of +92.03 kcal/mol. The ligand exhibited low conformational strain (+10.18 kcal/mol), suggesting minimal energetic cost upon binding. Additionally, ligand efficiency metrics—ΔG_bind per heavy atom (−2.54), surface-area-based efficiency (−3.81), and log-molecular-weight-based efficiency (−19.52)—further support compound 47’s favorable binding profile. Overall, the MM-GBSA analysis confirms that compound 47 forms a stable and efficient complex with the CysLT_1_ receptor, reinforcing its potential as a promising bioactive modulator for further development.

### 2.16. Antioxidant Study of Selected Lead Compounds

After a comprehensive evaluation involving 2D and 3D QSAR modeling, ADMET profiling, and molecular docking studies of 68 heterocyclic compounds as potential SRS-A antagonists, three top-performing candidates were shortlisted for further investigation. These include: 7-(3-(4-acetyl-3-hydroxy-2-propylphenoxy)-2-hydroxypropoxy)-4-oxo-8-propyl-4H-chromene-2-carboxylic acid (Compound **25**), 7-((5-(2-acetyl-3-hydroxyphenoxy)pentyl)oxy)-8-allyl-4-oxo-4H-chromene-2-carboxylic acid (Compound 41), and 7-(3-(4-acetyl-3-hydroxy-2-propylphenoxy)propoxy)-4-oxo-8-propyl-4H-chromene-2-carboxylic acid (Compound **47**). These compounds were prioritized based on their high predicted biological activity, strong binding affinity toward the CysLT_1_ receptor, and favorable ADMET properties. Among them, Compounds **25** and **41** were successfully synthesized following established synthetic protocols [8,11] and are also commercially available. Compounds **25** and **41** were synthesized through multi-step reactions involving the coupling of acetophenone derivatives with chromone esters, followed by ester hydrolysis. Specifically, Compound **25** was obtained via epoxide formation and subsequent reaction with ethyl chromone-2-carboxylate, while Compound **41** was synthesized by alkylation of ethyl 7-hydroxychromone ester using a bromopentyloxyacetophenone intermediate. Both final products were purified through hydrolysis and crystallization [8,11]. To experimentally evaluate their antioxidant potential, Compounds **25** and **41** were subjected to the DPPH (2,2-diphenyl-1-picrylhydrazyl) free radical scavenging assay. Serial dilutions of each compound (ranging from 5 to 100 μg/mL) in methanol were mixed with 0.3 mM DPPH solution, and the absorbance was measured at 517 nm after 30 min of incubation.

**Figure 25:**
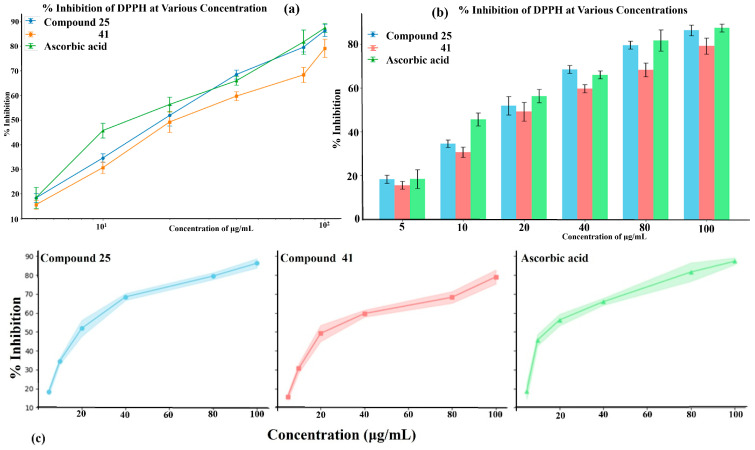
DPPH free radical scavenging activity of compounds 25 and 41 in comparison with ascorbic acid. Each value represents the mean ± standard deviation (*n* = 3). (**a**) Semi-logarithmic plot of scavenging percentage versus concentration. (**b**) Bar graph representation of DPPH radical scavenging (%) by compounds 25 and 41 and ascorbic acid at concentrations ranging from 5 to 100 μg/mL. Error bars indicate standard deviations (*n* = 3), and (**c**) Dose-dependent DPPH radical scavenging activity of Compound 25, Compound 41, and ascorbic acid at various concentrations (5–100 μg/mL). Each point represents the mean ± SD of three replicates. Compound 25 exhibited the highest antioxidant activity among the test compounds, closely approaching the standard ascorbic acid at 100 μg/mL.

Ascorbic acid was used as a standard reference antioxidant. At the lowest tested concentration (5 μg/mL), all samples showed modest DPPH scavenging activity, with Compound 25 exhibiting 18.34 ± 1.81% inhibition, Compound **41** showing 15.59 ± 1.83%, and ascorbic acid displaying 18.45 ± 4.23%. A dose-dependent increase in radical-scavenging activity was observed in the samples. At the highest concentration tested (100 μg/mL), Compound **25** achieved 86.32 ± 2.44% inhibition, closely approaching the activity of ascorbic acid (87.38 ± 1.80%), while Compound **41** reached 79.11 ± 3.71%. The IC_50_ values, defined as the concentration required to inhibit 50% of DPPH radicals, were calculated as 32.05 μg/mL for Compound 25, 41.72 μg/mL for Compound **41**, and 26.63 μg/mL for ascorbic acid. The lower IC_50_ value of Compound **25** compared to Compound **41** confirms its superior antioxidant activity. The dose–response relationships are illustrated in Figure 25a–c, which visually reinforces the quantitative findings and highlights that Compound 25 exhibits antioxidant activity comparable to that of ascorbic acid at higher concentrations, while Compound 41 is moderately less active. The enhanced antioxidant performance of Compound **25** may be attributed to its structural features, including the presence and position of electron-donating groups, which facilitate hydrogen or electron donation and promote radical stabilization. For reference, zafirlukast, a known CysLT_1_ receptor antagonist, has been reported to exhibit DPPH scavenging activity with an IC_50_ of 27.67 μg/mL [38], which is slightly weaker than that of Compound **25**. This supports the antioxidant potential of Compound **25**, which shows comparable or better radical scavenging efficacy.

The mechanistic overlap between oxidative stress and leukotriene-mediated inflammatory signaling supports the dual assessment of leukotriene receptor antagonism and antioxidant activity. Cysteinyl leukotrienes (CysLTs) are known to induce the generation of reactive oxygen species (ROS) in immune cells and respiratory epithelial cells, contributing to chronic inflammation and tissue damage [39,40]. In human airway smooth muscle cells (HASMC), LTD_4_-induced ROS production promotes EGFR transactivation and downstream ERK1/2 signaling, thereby driving cellular proliferation and inflammation [41]. Therefore, compounds exhibiting both CysLT_1_ antagonism and ROS-scavenging capabilities may provide synergistic therapeutic benefits in the treatment of inflammatory conditions such as asthma or allergic rhinitis. This dual-target strategy presents a promising framework for developing multifunctional drugs that modulate both receptor-mediated signaling and oxidative stress pathways.

## 3. Materials and Methods

### 3.1. Data Set

In this study, we employed experimental half-maximal inhibitory concentration (IC_50_) values to conduct a Quantitative Structure–Activity Relationship (QSAR) analysis. Our dataset comprised 68 heterocycle compounds (substituted (aryloxy)alkanoic acids and chromone-2-carboxylic acids), sourced from literature [8,11]. These IC_50_ values, initially reported in nanomolar units, span a range from approximately 2.07 nM to 306,443.49 nM, representing the concentration of each compound required to inhibit ileum contraction induced by Slow-Reacting Substance of Anaphylaxis (SRS-A) by 50%. This reflects a broad spectrum of inhibitory potencies within the studied compounds. The structures, pIC_50_, and IC_50_ values used in this analysis are provided in Table 1. For modeling purposes, the inhibitory activity, initially measured as IC_50_, was converted to pIC_50_ (-logIC_50_) values using the standard negative logarithmic scale. The structural representations of the compounds, expressed in SMILES notation, along with their associated pIC_50_ values, are comprehensively listed in Table S1. Predicted pIC_50_ values from the different QSAR models were back-transformed to IC_50_ for direct comparison with experimental data, as summarized in the Supplementary Materials (Tables S2 and S3 and Figure S1).

### 3.2. Calculation and Selection of Molecular Descriptors

The molecular structures of the studied compounds were first drawn in ChemDraw (version 17.0) and exported as Simplified Molecular Input Line Entry System (SMILES) strings. These SMILES were converted to Structure Data File (SDF) format using an online conversion server provided by Novartis Crop Protection AG. The SDFs were then uploaded to the Chemdes web tool (Central South University, China), which was used to calculate a total of 633 one-, two-, and three-dimensional molecular descriptors.

To mitigate overfitting and improve model robustness, a multi-step descriptor reduction protocol was applied. Descriptors with constant or near-constant variance (standard deviation < 0.001) or with any missing values were removed. Next, pairwise Pearson correlation analysis was performed, and for each pair of descriptors with |r| > 0.90, one descriptor was discarded, following the procedure reported by the Costa group [42] and implemented via the ChemSAR platform [43]. This filtering step reduced the descriptor pool from 633 to 28 variables, thereby minimizing redundancy while retaining the most informative features for QSAR modeling.

For subsequent multiple regression analyses, descriptor selection focused on variables that appeared most frequently in preliminary models and showed mechanistic relevance. To further limit overfitting, a compound-to-descriptor ratio of at least 10:1 was enforced. Given the 68-compound dataset, no more than six descriptors were retained in the final QSAR models [44,45], in line with commonly recommended limits for regression-based QSAR studies.

### 3.3. Dataset Splitting

After descriptor reduction, the 68-compound data set was partitioned into training and test sets. The k-means clustering algorithm, implemented within ChemMaster software (version 1.2) (CrescentSilico), was used to divide the compounds into clusters, ensuring chemical diversity across the resulting sets [46,47]. Specifically, one representative compound was randomly selected from each cluster to form the test set, resulting in 16 compounds. The remaining 52 compounds were assigned to the training set. This partitioning allowed for the construction of QSAR models using the training set and the subsequent assessment of their predictive performance on the independent test set. This clustering-based partitioning enabled QSAR models to be built on a structurally diverse training set and evaluated on an independent test set whose members still lay within the chemical and activity space of the training data, thereby defining a realistic applicability domain for model validation.

### 3.4. Model Development

In this study, the relationships between molecular structure and biological activity were investigated using four QSAR modeling approaches: multiple linear regression (MLR), multiple nonlinear regression (MNLR) with EXCEL-XLSTAT (Free Trial), QSARINS (granted a free license by the software author) [48], an artificial neural network (ANN) with MatlabV.2015 software (Free Trial), and atom-based 3D-QSAR models with Schrödinger Maestro on a Windows operating system. To evaluate the predictive power and statistical significance of these models, several key statistical criteria were employed. Specifically, a model was considered acceptable if it exhibited a statistically significant F-statistic of more than 0.33, indicating a robust relationship between the descriptor and activity. The coefficient of determination (R^2^) was used to assess the proportion of variance explained by the model, with higher values indicating a better fit. The accuracy of the predictions was quantified using the mean squared error (MSE), with lower values indicating better predictive performance. In addition, the adjusted coefficient of determination (R^2^ adj) was considered to account for the complexity of the model. Finally, a statistical significance level of *p* ≤ 0.05 was enforced to ensure that the observed relationships were not due to chance. Together, these criteria provide a rigorous framework for selecting reliable and predictive QSAR models to analyze the structure–activity relationships of the compounds under study. Multiple QSAR algorithms were therefore developed and compared to obtain a consensus view of the structure–activity relationships and to avoid over-reliance on any single modeling technique.

#### 3.4.1. Multiple Linear Regression (MLR)

Multiple linear regression (MLR) was employed to establish a linear relationship between molecular descriptors and the biological activity of the studied compounds. Due to its simplicity and robustness, MLR is a widely utilized technique in QSAR studies, particularly for the selection and identification of relevant molecular descriptors [49,50]. Furthermore, the descriptors identified through MLR were considered as potential input parameters for subsequent MNLR and ANN modeling. The MLR model assumes that the bioactivity (Y) is linearly dependent on a set of molecular descriptors (Xi) as defined by general Equation (3):

(3) $$Y=a_{0}+\sum_{i=1}^{n}a_{i}X_{i}$$

where Y represents the predicted biological activity (pIC_50_), Xi are the selected molecular descriptors, n is the total number of molecular descriptors included in the model, a0 is the intercept (constant term), ai are the coefficients associated with each descriptor Xi.

#### 3.4.2. Multiple Nonlinear Regression (MNLR)

A Multiple Linear Regression (MNLR) model was developed to establish a quantitative relationship between selected molecular descriptors and the inhibitory activity (pIC_50_) of the compounds studied [44,51]. Prior to model development, a correlation analysis was performed by calculating the Pearson correlation coefficient between each of the initial eight descriptors (Smax33, ndonr, Smax15, MRVSA6, Qindex, Es-tateVSA1, S17, and PEOEVSA1) and the pIC_50_ values. Based on this analysis, five descriptors exhibiting significant correlation (Smax33, ndonr, Smax15, S17, and PE-OEVSA1) were retained as independent variables for the MNLR model. The MNLR model was constructed using the training set of 54 compounds. The general form of the MNLR Equation (4) is as follows:

(4) $${pIC}_{50}=Intercept+a\cdot Smax33+b\cdot ndonr+c\cdot Smax15+d\cdot S17+e\cdot PEOEVSA1$$

where pIC_50_ is the predicted negative logarithm of the half-maximal inhibitory concentration; Intercept refers to the constant term of the model; a, b, c, d, and e are the regression coefficients corresponding to their respective descriptors; Smax33 represents a steric bulk descriptor; ndonr denotes the number of hydrogen bond donors; Smax15 is another steric descriptor; S17 is an atom-specific reactivity descriptor; and PEOEVSA1 refers to an orbital electronegativity descriptor. The MNLR model was implemented in Python using the scikit-learn library within a Jupyter Notebook (version 7.0) (Anaconda distribution). Model performance on the training set was evaluated by the coefficient of determination (R^2^) and mean squared error (MSE), and its predictive ability was assessed on an external test set of 14 compounds using the same metrics. A model including all eight initial descriptors was tested but rejected because it yielded unsatisfactory statistics under leave-one-out cross-validation (LOOCV).

#### 3.4.3. Support Vector Regression (SVR)

A support vector regression (SVR) model was developed to predict pIC_50_ values and to capture potential nonlinear relationships between molecular descriptors and biological activity. Unlike linear regression, SVR maps the input descriptors into a high-dimensional feature space via a kernel function and optimizes a margin-based loss, so it does not yield a simple explicit regression equation with interpretable coefficients. Conceptually, the predicted activity is expressed as a nonlinear function of the selected descriptors.

The SVR model was implemented in Python using the scikit-learn library with a radial basis function (RBF) kernel. Model performance was first assessed by leave-one-out cross-validation (LOOCV) on the complete set of 68 compounds and then by training on the full dataset to obtain the final predictive model. To evaluate the statistical significance and robustness of the SVR model, a Y-randomization test was carried out in which the pIC_50_ values were randomly permuted multiple times and new SVR models were generated; the distribution of R^2^ values for these randomized models was compared with that of the original model [52].

#### 3.4.4. Simplified Bayesian Model Averaging (BMA)-like Approach

For demonstration purposes, a simplified Bayesian model averaging (BMA)–like strategy was implemented to explore the potential benefit of model averaging for predicting pIC_50_ values. Multiple linear regression models were trained, each using a different combination of the five selected descriptors (Smax33, ndonr, Smax15, S17, and PEOEVSA1), thereby spanning the full model space defined by all possible descriptor subsets. The 68-compound dataset was randomly partitioned (random_state = 42) into a training set (N = 54) and a test set (N = 14) using an 80/20 split. For each linear model, the coefficient of determination (R^2^) on the test set was calculated and used as a proxy for the model’s posterior probability. Final pIC_50_ predictions for the test compounds were obtained by averaging the individual model predictions, weighted by their corresponding R^2^-based probabilities. This procedure represents a simplified BMA-like approach implemented solely for illustrative purposes; a rigorous BMA analysis would require formal Bayesian inference, explicit prior distributions, and more specialized software.

#### 3.4.5. Visualization of Chemical Space Colored by Activity

Two-dimensional embeddings of the 68 compounds were generated using t-distributed stochastic neighbor embedding (t-SNE), and the resulting coordinates were visualized in Python with the matplotlib.pyplot library. Each point in the scatter plot corresponds to a molecule, positioned according to the two t-SNE dimensions. Point color was mapped to the experimental pIC_50_ value using the continuous “viridis” colormap, and a color bar was added to indicate the pIC_50_ scale. The axes were labeled t-SNE Dimension 1 and t-SNE Dimension 2, and the plot was titled “t-SNE visualization colored by pIC_50_”, with a grid included to aid readability. This visualization provides an overview of the explored chemical space and allows visual inspection of how molecular distribution relates to biological activity [53,54,55].

#### 3.4.6. Tanimoto Similarity Analysis and Heatmap Visualization

Structural similarity among the molecules was quantified by calculating pairwise Tanimoto coefficients based on their MACCS fingerprints. MACCS fingerprints are 166-bit binary vectors in which each bit encodes the presence or absence of a predefined structural key. The Tanimoto coefficient *T* between two fingerprints *A* and *B* was computed according to Equation (5):

T = N(A∩B)/N(A) + N(B) − N(A∩B)(5)

where *N*(*A*∩*B*) is the number of bits set to 1 in both fingerprints, and *N*(*A*) and *N*(*B*) are the numbers of bits set to 1 in fingerprints *A* and *B*, respectively.

The resulting 68 × 68 Tanimoto similarity matrix was visualized as a heatmap using the Seaborn library (version 0.13) in Python. Color intensity in the heatmap reflects the magnitude of the similarity score, ranging from low similarity (dark purple) to high similarity (bright yellow). This visualization facilitates rapid assessment of structural relationships within the compound library and highlights clusters of molecules with high structural similarity [56,57].

#### 3.4.7. Artificial Neural Network (ANN)

An artificial neural network (ANN) was constructed to forecast pIC_50_ values for a dataset of 68 organic compounds, employing eight molecular descriptors as input variables: Smax33, ndonr, Smax15, MRVSA6, Qindex, EstateVSA1, S17, and PEOEVSA1. The network’s architecture featured an input layer containing eight neurons, a single hidden layer with ten neurons, and an output layer with a solitary neuron that represented the predicted pIC_50_ (as depicted in Figure 1). Network training and subsequent evaluation were conducted using MATLAB R2015. Three optimization algorithms were used: scaled conjugate gradient (SCG), Levenberg–Marquardt (LM), and Bayesian regularization (BR) [58]. During training, the connection weights and biases were adjusted repeatedly to minimize the difference between the predicted and experimentally determined pIC_50_ values.

After training, residuals (predicted–experimental pIC_50_) were calculated for each compound and each algorithm (Residual-SCG, Residual-LM, Residual-BR). The corresponding correlation coefficients (R values) for the training, test, and overall data sets, together with residual statistics (Table 1 and Figure 1), were used to assess the predictive accuracy and generalization ability of each ANN model.

### 3.5. Atom-Based 3D-QSAR Model Construction

An atom-based three-dimensional Quantitative Structure–Activity Relationship (3D-QSAR) model was constructed and validated using a combined computational approach. This included pharmacophore hypothesis generation and ligand alignment within the Schrödinger Maestro (Phase) environment, followed by Python-based post-processing for QSAR model development, thorough validation, and in-depth analysis of atom-level contributions to activity [59,60,61].

#### 3.5.1. Generation of Pharmacophore Hypotheses

Pharmacophore modeling was carried out using the Develop Hypothesis module in Schrödinger Maestro (version 2023.1) [61]. A training set of six biologically active ligands was used to derive common pharmacophoric features. Hypothesis generation was restricted to models containing five to seven features, including hydrogen-bond acceptors, aromatic rings, and hydrophobic groups. Among the generated models, the highest-scoring hypothesis, AAAHHR_1, consisting of three hydrogen-bond acceptors (A1, A2, A4), two aromatic rings (R9, R10), and one hydrophobic group (H5), was selected for subsequent atom-based 3D-QSAR analysis

#### 3.5.2. Ligand Alignment to the Developed Pharmacophore

After selection of the AAAHHR_1 pharmacophore hypothesis, all 68 ligands were spatially aligned to this model using the “Align Ligands to Hypothesis” option in Phase (Schrödinger Suite). Each molecule was fitted to the pharmacophore features of AAAHHR_1 to obtain a consistent 3D orientation across the dataset. This common alignment was essential for reliable atom-based descriptor calculation and for subsequent construction of the 3D-QSAR model [60].

#### 3.5.3. Methodology for Atom-Based QSAR Model Construction

Atom-based 3D-QSAR modeling was carried out in Phase using the QSAR → Atom-Based workflow. A cubic grid with 1.0 Å spacing was applied around the aligned ligands to generate atom-based descriptors, and partial least squares (PLS) regression was used to relate these descriptors to pIC_50_ values. Models with one, two, and three latent PLS factors were evaluated, and descriptors with |t| < 2.0 were automatically excluded to improve statistical robustness and interpretability. The 68-ligand dataset was randomly divided into an 80% training set and a 20% test set using the “Split into Training and Test Sets” option in Phase. The final model, employing three PLS factors, was selected on the basis of its superior internal cross-validation statistics [59].

#### 3.5.4. Model Validation and Performance Metrics

The predictive capability of the generated model was validated using an external test set and the Test function in Maestro. Model performance was quantified using the Root Mean Squared Error (RMSE), Predictive R^2^ (Q^2^), and Pearson’s r [59]. To ensure the robustness of these metrics, they were independently computed in Python using the mean_squared_error and r2_score functions from scikit-learn, and numpy.corrcoef for correlation assessment [62]. Visualization of predicted versus experimental pIC_50_ values was initially performed with the QSAR Scatter Plot tool in Maestro and subsequently enhanced for publication-quality figures using the matplotlib and seaborn libraries in Python [53].

#### 3.5.5. Generation of Feature Importance Maps and Contour Visualization

The QSAR Visualization module in Maestro was used to generate 3D contour maps superimposed on the aligned ligands, highlighting regions where specific chemical features are predicted to enhance (blue contours) or reduce (red contours) biological activity. These maps provided a spatial interpretation of the atom-based 3D-QSAR model. In a complementary analysis, contributions of different atom-type descriptors, such as hydrophobic regions, hydrogen-bond donors, electron-withdrawing groups, and negatively charged centers, were quantified in Python using the pandas library and displayed as bar charts generated with seaborn.barplot(), allowing identification of the most influential structural characteristics [53].

#### 3.5.6. SMILES Standardization and Dataset Filtering Procedures

To ensure structural consistency in the datasets, the SMILES strings were standardized and matched by the Python functions set.intersection() and pandas.merge(). In cases of structural naming inconsistency, the Unique SMILES column of the project table was used. The filtered dataset is then carried out by the activity of the compound, with active compounds defined as pIC_50_ ≥ 6.0 and inactive as pIC_50_ < 6.0. The filtered dataset is then exported for the analysis and reporting of QSAR [53,63].

### 3.6. Drug-Likeness and ADMET Prediction Studies

Evaluation of physicochemical properties, drug likeness, and pharmacokinetic behavior is an essential early step in drug development because it helps to eliminate compounds with unfavorable biological profiles [64]. Pharmacokinetic assessment typically considers absorption, distribution, metabolism, excretion, and toxicity (ADMET), which together define the in vivo behavior of a candidate molecule [65]. In the present work, the ADMETlab 2.0 web server (https://admetmesh.scbdd.com/; accessed on 15 June 2025) was employed to obtain a comprehensive panel of ADMET descriptors and to predict potential toxicity risks [66]. Drug likeness of the 68 substituted (aryloxy)alkanoic acids and chromone 2 carboxylic acids was further examined using the SwissADME and pkCSM platforms, available at http://www.swissadme.ch (accessed on 15 June 2025) and https://biosig.lab.uq.edu.au/pkcsm/ (accessed on 20 June 2025), respectively [67]. These analyses applied standard drug likeness filters, including the Lipinski, Ghose, Veber, Egan, and Muegge rules [65], while pkCSM additionally used graph-based molecular signatures to train and apply its predictive models.

### 3.7. Molecular Docking and Molecular Dynamics Simulation Investigations

A molecular docking study was carried out to predict the binding interactions between the X-ray free electron laser determined crystal structure of human cysteinyl leukotriene receptor 1 (CysLT1R) in complex with zafirlukast and a library of substituted (aryloxy)alkanoic acids and chromone 2 carboxylic acids, which act as antagonists of the slow reacting substance of anaphylaxis. Docking simulations were performed using the MzDock (version 2.0) program, a graphical pipeline for the Windows operating system that applies several force fields (MMFF94, MMFF94s, UFF, GAFF, and Ghemical) for energy minimization [68]. The CysLT1R crystal structure co-crystallized with zafirlukast (PDB ID: 6RZ5) served as the receptor template, and the co-crystallized ligand was used as a reference compound to validate the protocol. To confirm the reliability of the docking workflow and to identify key binding site residues, zafirlukast was redocked under the same conditions, and the reproduced pose closely matched the experimentally observed orientation.

Selected high scoring docking poses were subsequently examined to characterize interactions with the slow reacting substance of anaphylaxis (SRS A), a mixture of leukotrienes with potent bronchoconstrictive activity and a major role in allergic responses such as asthma. Detailed analyses of the contacts between docked ligands and specific amino acid residues were performed in Discovery Studio [69], providing insight into the molecular determinants of receptor antagonism.

In the case of molecular dynamics simulation, an orthorhombic simulation box was prepared in the System Builder so that all protein atoms were at least 10 Å from the box boundaries. The complex was solvated with explicit TIP3P water, and counterions were added to neutralize the system. Energy minimization was then performed using the OPLS3e force field until the maximum force converged to 1 kcal/mol/Å. Constant temperature and pressure were maintained with a Nose–Hoover chain thermostat [70] and a Martyna–Tobias–Klein barostat during the production run. Molecular dynamics trajectories were propagated with a 2 fs time step, coordinates were saved every 10 ps, and the resulting simulations were analyzed in Maestro (Schrödinger, LLC, New York, NY, USA).

### 3.8. Antioxidant Activity Assay (DPPH Method)

Compounds 25 and 41, which were chosen based on the predictions of the QSAR model, were synthesized and tested for their antioxidant activity by the 1,1-Diphenyl-2-Picrylhydrazyl (DPPH) assay method [71,72]. To test the antioxidant activity of the compounds, a stock solution of each compound was made at a concentration of 1 mg/mL in methanol. It was then diluted to a range of 5–100 µg/mL. To each 3.0 mL of the diluted samples, 1.0 mL of a DPPH solution (0.3 mmol/L in methanol) was added. The resulting mixtures were then incubated at room temperature for a period of 30 min. Absorbance was then recorded at 517 nm using a UV-Vis 1800 spectrophotometer ((Shimadzu, Kyoto, Japan). The scavenging activity (%) was calculated according to Equation (6):

Scavenging activity (%) = (A0 − A1)/A0 × 100(6)

where A_0_ is the absorbance of the DPPH solution alone and A_1_ is the absorbance in the presence of the test compound or L-ascorbic acid (standard). IC_50_ values, defined as the concentration required to quench 50% of DPPH radicals, were obtained from the dose–response curves by linear regression, and all measurements were performed in triplicate.

## 4. Conclusions

An integrative computational framework was employed to evaluate 68 substituted (aryloxy)alkanoic acids and chromone-2-carboxylic acids as potential slow-reacting substance of anaphylaxis (SRS-A) antagonists. The study combined 2D and 3D-QSAR modeling, molecular docking against the CysLT_1_ receptor (PDB ID: 6RZ5), and in silico ADMET profiling. The developed atom-based 3D-QSAR model demonstrated strong statistical validity (R^2^ = 0.9524, Q^2^ = 0.5382, RMSE = 0.2813), identifying steric and hydrogen-bonding features (e.g., Smax33, ndonr, Smax15, MRVSA6) as key contributors to biological activity. Molecular docking identified several potent ligands, notably compound 25 (BE = −9.5 kcal/mol, pIC_50_ = 7.99), compound 41 (BE = −10 kcal/mol, pIC_50_ = 6.96), and compound 47 (BE = −9.4 kcal/mol, pIC_50_ = 8.68), all of which demonstrated strong binding affinities and high predicted bioactivities, showing consistent trends across docking and QSAR analyses. The dynamic stability of the 47–CysLT_1_ complex was further confirmed by molecular dynamics simulation, which supports its strong and sustained interaction with the receptor. Despite strong docking and QSAR predictions, only a subset of compounds satisfied all six ADMET drug-likeness rules. Compound 63 passed all filters with no violations and a high QED (0.410), while Compound 25 met most parameters with a single Lipinski violation. Compound 41 exhibited partial rule compliance but maintained strong pharmacological potential. Based on these integrative findings, Compound 25 was prioritized for experimental antioxidant evaluation and demonstrated significant free radical scavenging activity, comparable to ascorbic acid.

Collectively, this study identifies Compounds 25 and 41 as promising dual-action lead molecules with both anti-inflammatory and antioxidant potential, while compound 47 demonstrated strong dynamic stability and favorable binding energy in MD simulation, supporting its candidacy for further development. The combined QSAR–docking–ADMET–antioxidant approach provides a robust platform to guide future synthesis, lead optimization, and experimental validation of novel SRS-A antagonists.

## Figures and Tables

**Table 1:** Data Set for Analysis: Structures, pIC_50_, and IC_50_ Values.

Molecule Data (Structure, Molecule Serial Number or Compound Number (S/N)), pIC_50_, IC_50_)			
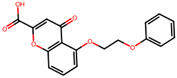 Molecule 1 pIC_50_: 3.5136 IC_50_: 3.0647 × 10^−4^	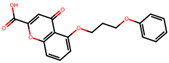 Molecule 2 pIC_50_: 3.9878 IC_50_: 1.0284 × 10^−4^	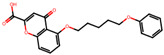 Molecule 3 pIC_50_: 4.3233 IC_50_: 4.7505 × 10^−5^	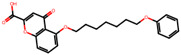 Molecule 4 pIC_50_: 5.6951 IC_50_: 2.018 × 10^−6^
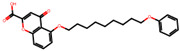 Molecule 5 pIC_50_: 4.8497 IC_50_: 1.4135 × 10^−5^	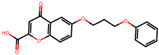 Molecule 6 pIC_50_: 5.0336 IC_50_: 9.2557 × 10^−6^	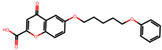 Molecule 7 pIC_50_: 4.9752 IC_50_: 1.0587 × 10^−5^	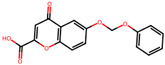 Molecule 8 pIC_50_: 4.675 IC_50_: 2.1136 × 10^−5^
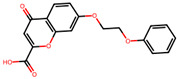 Molecule 9 pIC_50_: 4.3375 IC_50_: 4.597 × 10^−5^	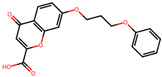 Molecule 10 pIC_50_: 5.3858 IC_50_: 4.1137 × 10^−6^	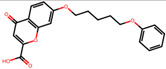 Molecule 11 pIC_50_: 5.9642 IC_50_: 1.0858 × 10^−6^	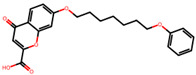 Molecule 12 pIC_50_: 5.3429 IC_50_: 4.5405 × 10^−6^
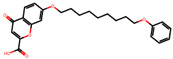 Molecule 13 pIC_50_: 5.3726 IC_50_: 4.2404 × 10^−6^	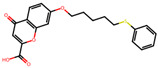 Molecule 14 pIC_50_: 5.9828 IC_50_: 1.0404 × 10^−6^	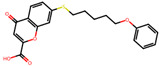 Molecule 15 pIC_50_: 6.1077 IC_50_: 7.8034 × 10^−7^	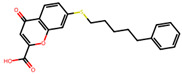 Molecule 16 pIC_50_: 5.7882 IC_50_: 1.6284 × 10^−6^
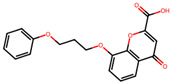 Molecule 17 pIC_50_: 3.8329 IC_50_: 1.4692 × 10^−4^	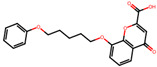 Molecule 18 pIC_50_: 4.6121 IC_50_: 2.4431 × 10^−5^	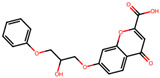 Molecule 19 pIC_50_: 4.7068 IC_50_: 1.9645 × 10^−5^	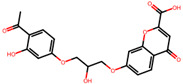 Molecule 20 pIC_50_: 4.1702 IC_50_: 6.7574 × 10^−5^
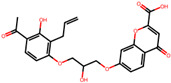 Molecule 21 pIC_50_: 6.6575 IC_50_: 2.2006 × 10^−7^	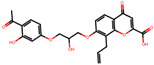 Molecule 22 pIC_50_: 5.139 IC_50_: 7.2618 × 10^−6^	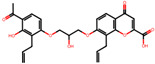 Molecule 23 pIC_50_: 6.9952 IC_50_: 1.0111 × 10^−7^	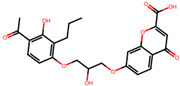 Molecule 24 pIC_50_: 6.7563 IC_50_: 1.7527 × 10^−7^
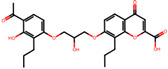 Molecule 25 pIC_50_: 7.9987 IC_50_: 1.003 × 10^−8^	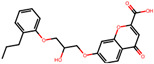 Molecule 26 pIC_50_: 5.2993 IC_50_: 5.02 × 10^−6^	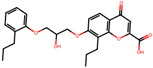 Molecule 27 pIC_50_: 7.0005 IC_50_: 9.9889 × 10^−8^	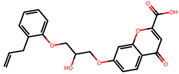 Molecule 28 pIC_50_: 5.3429 IC_50_: 4.541 × 10^−6^
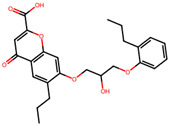 Molecule 29 pIC_50_: 5.4398 IC_50_: 3.6323 × 10^−6^	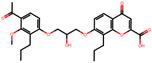 Molecule 30 pIC_50_: 4.0565 IC_50_: 8.7796 × 10^−5^	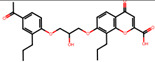 Molecule 31 pIC_50_: 6.5074 IC_50_: 3.1087 × 10^−7^	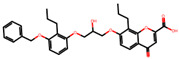 Molecule 32 pIC_50_: 5.7377 IC_50_: 1.8295 × 10^−6^
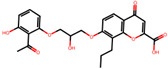 Molecule 33 pIC_50_: 6.1823 IC_50_: 6.5726 × 10^−7^	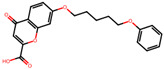 Molecule 34 pIC_50_: 5.9328 IC_50_: 1.1673 × 10^−6^	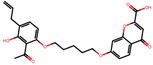 Molecule 35 pIC_50_: 5.7146 IC_50_: 1.9293 × 10^−6^	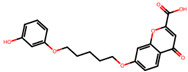 Molecule 36 pIC_50_: 5.4087 IC_50_: 3.9024 × 10^−6^
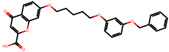 Molecule 37 pIC_50_: 5.6762 IC_50_: 2.1075 × 10^−6^	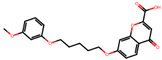 Molecule 38 pIC_50_: 5.4242 IC_50_: 3.765 × 10^−6^	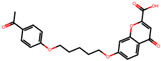 Molecule 39 pIC_50_: 5.0692 IC_50_: 8.5278 × 10^−6^	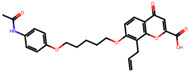 Molecule 40 pIC_50_: 5.0769 IC_50_: 8.3781 × 10^−6^
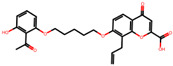 Molecule 41 pIC_50_: 6.9699 IC_50_: 1.0719 × 10^−7^	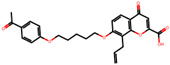 Molecule 42 pIC_50_: 5.2735 IC_50_: 5.3277 × 10^−6^	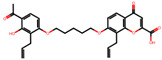 Molecule 43 pIC_50_: 5.6147 IC_50_: 2.4282 × 10^−6^	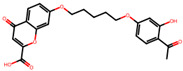 SN: Molecule 44 pIC_50_: 4.7973 IC_50_: 1.5947 × 10^−5^
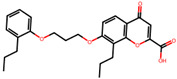 Molecule 45 pIC_50_: 6.3726 IC_50_: 4.2404 × 10^−7^	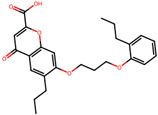 Molecule 46 pIC_50_: 4.3726 IC_50_: 4.2404 × 10^−5^	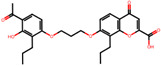 Molecule 47 pIC_50_: 8.6835 IC_50_: 2.0725 × 10^−9^	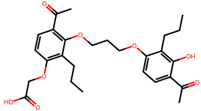 Molecule 48 pIC_50_: 5.699 IC_50_: 2.0 × 10^−6^
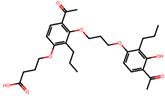 Molecule 49 pIC_50_: 6.699 IC_50_: 2.0 × 10^−7^	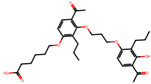 Molecule 50 pIC_50_: 6.0 IC_50_: 1.0 × 10^−6^	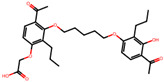 Molecule 51 pIC_50_: 7.0 IC_50_: 1.0 × 10^−7^	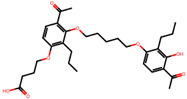 Molecule 52 pIC_50_: 6.0 IC_50_: 1.0 × 10^−6^
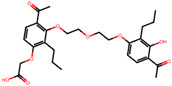 Molecule 53 pIC_50_: 6.699 IC_50_: 2.0 × 10^−7^	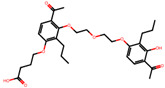 Molecule 54 pIC_50_: 7.0 IC_50_: 1.0 × 10^−7^	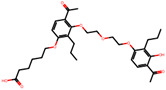 Molecule 55 pIC_50_: 6.699 IC_50_: 2.0 × 10^−7^	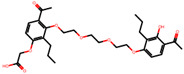 Molecule 56 pIC_50_: 6.0 IC_50_: 1.0 × 10^−6^
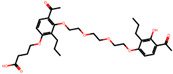 Molecule 57 pIC_50_: 6.301 IC_50_: 5.0 × 10^−7^	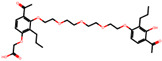 Molecule 58 pIC_50_: 6.5229 IC_50_: 3.0 × 10^−7^	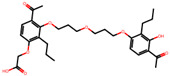 Molecule 59 pIC_50_: 6.3979 IC_50_: 4.0 × 10^−7^	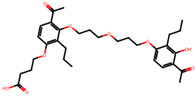 Molecule 60 pIC_50_: 6.699 IC_50_: 2.0 × 10^−7^
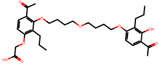 Molecule 61 pIC_50_: 6.301 IC_50_: 5.0 × 10^−7^	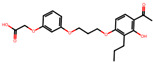 Molecule 62 pIC_50_: 6.301 IC_50_: 5.0 × 10^−7^	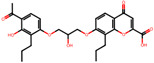 Molecule 63 pIC_50_: 7.4559 IC_50_: 3.5 × 10^−8^	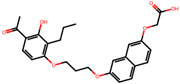 Molecule 64 pIC_50_: 6.5229 IC_50_: 3.0 × 10^−7^
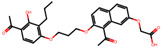 Molecule 65 pIC_50_: 6.699 IC_50_: 2.0 × 10^−7^	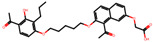 Molecule 66 pIC_50_: 6.699 IC_50_: 2.0 × 10^−7^	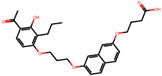 Molecule 67 pIC_50_: 5.301 IC_50_: 5.0 × 10^−6^	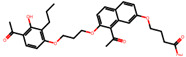 Molecule 68 pIC_50_: 7.0969 IC_50_: 8.0 × 10^−8^

S/N: Serial Number/Compound Number.

**Table 2:** The observed (pIC_50_) and predicted (pred pIC_50_) values, along with their residuals (Res.), of biological activities from various QSAR models (such as MLR and MNLR, including Artificial Neural Networks (ANNs)) developed based on the training and testing sets are shown in bold.

Compd.	pIC_50_	MLR		MNLR		ANN						BE kcal/mol
		Pred pIC_50_	Res	Pred pIC_50_	Res	Pred pIC_50_ (LM)	Res	Pred pIC_50_ (SCG)	Res	Pred pIC_50_ (BR)	Res	
1k	3.51	4.59	1.08	4.50	0.99	4.67	1.15	4.84	1.33	5.02	1.52	−7.5
2	3.98	4.68	0.70	4.68	0.69	4.79	0.80	4.92	0.93	5.02	1.03	−8
3	4.32	4.66	0.34	4.89	0.56	5.00	0.68	4.91	0.58	5.06	0.74	−7.8
4r	5.69	4.68	−1.01	4.92	−0.77	5.09	−0.61	4.91	−0.78	5.07	−0.62	−8
5k	4.84	4.70	−0.14	5.06	0.21	5.12	0.27	4.91	0.06	5.08	0.24	−9
6rk	5.03	4.65	−0.38	4.89	−0.14	5.42	0.39	5.04	0.01	5.05	0.02	−5.5
7	4.97	4.73	−0.24	5.20	0.23	5.50	0.53	5.09	0.12	5.05	0.08	−6.6
8r	4.67	4.57	−0.10	4.36	−0.32	4.98	0.30	4.94	0.27	5.02	0.35	−8.6
9	4.33	4.73	0.40	4.76	0.42	5.05	0.72	5.01	0.67	5.00	0.66	−7
10k	5.38	4.76	−0.62	4.86	−0.52	5.21	−0.18	5.03	−0.35	5.01	−0.37	−8
11r	5.96	4.74	−1.22	5.04	−0.93	5.27	−0.70	5.01	−0.96	5.06	−0.90	−5.1
12r	5.34	4.72	−0.62	5.13	−0.22	5.41	0.06	4.98	−0.36	5.09	−0.25	−7.8
13rk	5.37	4.73	−0.64	5.18	−0.19	5.44	0.07	4.98	−0.39	5.10	−0.27	−6.8
14	5.98	4.78	−1.20	5.58	−0.41	6.33	0.35	5.19	−0.80	5.14	−0.84	−8.9
15	6.10	4.86	−1.24	5.62	−0.48	6.29	0.18	5.22	−0.89	5.13	−0.97	−6.3
16	5.78	5.01	−0.77	6.23	0.44	6.69	0.90	5.40	−0.39	5.18	−0.60	−6.4
17k	3.83	4.69	−0.86	4.64	0.81	4.54	0.71	4.81	0.98	5.02	1.19	−8.2
18r	4.61	6.50	1.89	4.84	0.23	4.95	0.34	4.90	0.29	5.02	0.42	−8.5
19	4.70	5.36	0.66	4.69	−0.01	4.68	−0.03	4.99	0.28	5.42	0.71	−9.2
20	4.17	5.88	1.71	5.48	1.31	4.48	0.31	5.07	0.90	5.96	1.79	−9
21	6.65	6.19	−0.46	5.92	−0.74	6.58	−0.08	6.02	−0.64	6.07	−0.58	−9.3
22	5.13	6.09	0.96	5.99	0.85	5.58	0.44	6.08	0.94	6.07	0.94	−9.6
23	6.99	6.29	−0.70	6.94	−0.06	6.84	−0.16	7.09	0.10	6.19	−0.80	−9.5
24	6.75	6.23	−0.52	6.68	−0.07	6.85	0.09	6.15	−0.61	6.13	−0.62	−8.8
25	7.99	6.45	−1.54	7.43	−0.57	8.31	0.32	6.85	−1.15	6.23	−1.76	−9.5
26	5.29	5.68	0.39	5.54	0.24	5.09	−0.21	5.80	0.50	5.64	0.34	−9.2
27r	7.00	5.97	−1.03	5.52	−1.48	5.83	−1.17	6.08	−0.92	5.78	−1.22	−9.4
28r	5.34	5.74	0.40	5.31	−0.03	5.03	−0.32	5.29	−0.05	5.58	0.24	−9
29rk	5.43	6.00	0.57	6.17	0.73	6.08	0.64	6.17	0.73	5.80	0.36	−9.1
30	4.05	6.45	2.40	6.62	2.56	5.00	0.94	6.56	2.50	6.25	2.20	−9.2
31	6.50	6.48	−0.02	6.42	−0.09	5.25	−1.26	6.41	−0.09	6.21	−0.29	−9
32	5.73	6.57	0.84	5.35	−0.39	5.44	−0.30	6.48	0.75	6.07	0.33	−10
33	6.18	6.09	−0.09	5.72	−0.46	5.80	−0.38	5.61	−0.57	6.04	−0.14	−9.8
34	5.93	4.74	−1.19	5.04	−0.90	5.27	−0.66	5.01	−0.92	5.06	−0.87	−8.1
35	5.71	6.14	0.43	6.18	0.46	8.64	2.93	5.68	−0.03	5.90	0.19	−8.6
36	5.40	5.27	−0.13	4.80	−0.61	5.42	0.01	5.48	0.07	5.30	−0.11	−9.3
37	5.67	5.40	−0.27	5.44	−0.23	6.39	0.71	5.94	0.26	5.27	−0.40	−9.5
38rk	5.42	4.88	−0.54	6.02	0.59	6.20	0.77	5.52	0.10	5.14	−0.28	−7.8
39	5.06	5.39	−0.33	5.04	−0.03	4.33	−0.74	5.26	0.19	5.45	0.39	−8.6
40	5.07	5.391	0.32	6.14	1.06	5.30	0.22	5.75	0.67	5.71	0.64	−9
41	6.96	5.91	−1.05	5.14	−1.83	5.60	−1.37	5.47	−1.50	5.78	−1.19	−10
42k	5.27	5.69	0.42	5.12	−0.15	5.64	0.36	5.46	0.19	5.56	0.29	−8.3
43r	5.61	6.42	0.81	6.45	0.84	5.73	0.12	6.10	0.49	6.01	0.40	−9.1
44	4.79	5.74	0.95	5.36	0.56	5.09	0.30	5.13	0.34	5.74	0.95	−9.1
45r	6.37	5.18	−1.19	4.69	−1.68	6.29	−0.09	5.87	−0.50	5.42	−0.95	−8.8
46	4.37	5.18	0.81	5.84	1.47	6.33	1.96	5.95	1.58	5.42	1.05	−8.9
47k	8.68	6.35	−2.33	6.38	−2.30	8.60	−0.08	6.25	−2.43	6.09	−2.58	−9.4
48k	5.69	6.38	0.69	6.58	0.88	6.22	0.52	6.25	0.55	6.25	0.56	−7.7
49k	6.69	6.62	−0.07	6.44	−0.26	6.73	0.03	6.41	−0.28	6.45	−0.25	−8
50	6	6.75	0.75	6.53	0.53	6.48	0.48	6.49	0.49	6.46	0.47	−7.1
51	7	6.57	−0.43	6.26	−0.74	6.29	−0.71	6.36	−0.64	6.25	−0.74	−8.4
52	6	6.74	0.74	6.47	0.47	6.44	0.44	6.52	0.52	6.47	0.48	−7.8
53	6.69	6.15	−0.54	6.47	−0.23	6.29	−0.41	6.28	−0.42	6.32	−0.38	−7.9
54	7	6.33	−0.67	6.55	−0.45	6.61	−0.39	6.54	−0.46	6.53	−0.46	−8.1
55	6.69	6.46	−0.23	6.31	−0.39	6.36	−0.34	6.62	−0.08	6.55	−0.15	−7.6
56r	6	6.23	0.23	6.58	0.58	6.21	0.21	6.30	0.30	6.30	0.30	−7.7
57k	6.30	6.40	0.10	6.79	0.49	6.58	0.28	6.53	0.23	6.51	0.22	−7.3
58	6.52	6.35	−0.17	6.51	−0.02	6.11	−0.41	6.27	−0.25	6.25	−0.26	−6.9
59k	6.39	6.53	0.14	6.43	0.03	6.24	−0.16	6.32	−0.08	6.23	−0.16	−8.4
60	6.69	6.46	−0.23	6.24	−0.46	6.61	−0.09	6.46	−0.24	6.45	−0.25	−7.6
61	6.30	6.40	0.10	6.33	0.03	6.28	−0.03	6.40	0.10	6.26	−0.03	−6.9
62	6.30	6.18	−0.12	6.41	0.11	4.93	−1.38	5.93	−0.37	5.93	−0.37	−8
63r	7.45	6.45	−1.00	7.90	0.44	8.31	0.86	6.85	−0.61	6.23	−1.22	−9.6
64	6.52	6.35	−0.17	6.19	−0.33	6.33	−0.19	6.70	0.18	6.01	−0.51	−9
65	6.69	6.33	−0.36	6.45	−0.24	7.12	0.42	6.41	−0.29	6.18	−0.51	−8.6
66	6.69	6.51	−0.18	6.49	−0.21	6.61	−0.09	6.44	−0.26	6.18	−0.52	−7.3
67rk	5.3	6.53	1.23	6.61	1.31	6.82	1.51	6.30	1.00	6.17	0.87	−9.3
68	7.09	6.54	−0.55	6.98	−0.12	7.22	0.13	6.33	−0.76	6.35	−0.74	−8.8

Random partitioning (r) and K-means clustering (k) are tests applied to compounds; some compounds belong to both r and k.

**Table 3:** Summary of Statistical Performance Metrics for All QSAR Models.

Model	R^2^ (Train)	R^2^ (External/Test)	Q^2^ (CV or External)	RMSE	MSE	Notes
MLR	0.981	Not applicable (LOOCV)	0.973 (LOOCV)	0.893	0.797	Leave-One-Out Cross-Validation applied
MNLR	0.864	0.808	–	–	0.181(Train), 0.276 (Test)	External test set used
SVR (LOOCV)	0.4092	–	–	–	0.6051	LOOCV and Y-randomization validated
SVR (Full)	0.6032	–	–	–	0.4064	Full dataset used for reference only
ANN (LM)	0.917	0.047	–	–	–	Overfitting observed
ANN (SCG)	0.633	0.861	–	–	–	Best generalization among ANN models
ANN (BR)	0.619	0.735	–	–	–	Moderate generalization
3D-QSAR	0.9524	–	0.5382 (PLS CV)	0.2813	–	3 PLS components; 68 ligands used

Note: “(–)” indicates that no separate external test set was used; model performance was assessed using internal validation techniques (e.g., Leave-One-Out Cross-Validation, LOOCV). RMSE and MSE values were not explicitly calculated for some ANN and SVR models, as their performance was primarily evaluated based on correlation coefficients (R) and residual analysis.

**Table 4:** Predicted activities 1, 2, and 3 refer to the predicted pIC_50_ values from three atom-based 3D-QSAR models developed using different training/test set divisions and alignment strategies. Their corresponding IC_50_ values are also shown.

Activity Provided (pIC_50_)	Activity Provided (IC_50_)	Predicted Activity 1 (pIC_50_)	Predicted Activity 1 (IC_50_)	Predicted Activity 2 (pIC_50_)	Predicted Activity 2 (IC_50_)	Predicted Activity 3 (pIC_50_)	Predicted Activity 3 (IC_50_)
3.514	3.06 × 10^−4^	4.93233	1.17 × 10^−5^	4.45651	3.50 × 10^−5^	4.49051	3.23 × 10^−5^
3.988	1.03 × 10^−4^	4.83579	1.46 × 10^−5^	4.17202	6.73 × 10^−5^	4.10478	7.86 × 10^−5^
4.323	4.75 × 10^−5^	4.74783	1.79 × 10^−5^	4.81442	1.53 × 10^−5^	4.81539	1.53 × 10^−5^
4.85	1.41 × 10^−5^	4.26787	5.40 × 10^−5^	4.80836	1.55 × 10^−5^	4.86764	1.36 × 10^−5^
4.975	1.06 × 10^−5^	5.3325	4.65 × 10^−6^	4.61348	2.44 × 10^−5^	4.78719	1.63 × 10^−5^
4.675	2.11 × 10^−5^	4.89215	1.28× 10^−5^	4.4022	3.96 × 10^−5^	4.54507	2.85 × 10^−5^
4.338	4.59 × 10^−5^	3.99936	1.00× 10^−4^	4.08137	8.29 × 10^−5^	4.27424	5.32 × 10^−5^
3.833	1.47 × 10^−4^	2.76825	1.71× 10^−3^	3.39261	4.05 × 10^−4^	3.36508	4.31 × 10^−4^
4.612	2.44 × 10^−5^	3.42786	3.73 × 10^−4^	4.20852	6.19 × 10^−5^	4.15113	7.06 × 10^−5^
4.612	2.44 × 10^−5^	4.92238	1.20 × 10^−5^	5.06378	8.63 × 10^−6^	4.78211	1.65 × 10^−5^
4.072	8.47 × 10^−5^	6.53801	2.90 × 10^−7^	6.56731	2.71 × 10^−7^	6.74556	1.80 × 10^−7^
4.072	8.47 × 10^−5^	5.48678	3.26 × 10^−6^	4.62797	2.36 × 10^−5^	4.28001	5.25 × 10^−5^
4.707	1.96 × 10^−5^	5.85252	1.40 × 10^−6^	5.85455	1.40 × 10^−6^	5.87296	1.34 × 10^−6^
4.707	1.96 × 10^−5^	5.58178	2.62 × 10^−6^	5.2581	5.52 × 10^−6^	5.06978	8.52 × 10^−6^
6.657	2.20 × 10^−7^	6.2235	5.98 × 10^−7^	6.59032	2.57 × 10^−7^	6.44012	3.63 × 10^−7^
6.657	2.20 × 10^−7^	6.32444	4.74 × 10^−7^	6.80489	1.57 × 10^−7^	6.56923	2.70 × 10^−7^
6.995	1.01 × 10^−7^	6.72526	1.88 × 10^−7^	7.26976	5.37 × 10^−8^	7.07778	8.36 × 10^−8^
6.995	1.01 × 10^−7^	6.3286	4.69 × 10^−7^	6.77081	1.70 × 10^−7^	6.78189	1.65 × 10^−7^
7.999	1.00 × 10^−8^	6.67522	2.11 × 10^−7^	7.29729	5.04 × 10^−8^	7.57146	2.68 × 10^−8^
2.299	5.02 × 10^−3^	2.71621	1.92 × 10^−3^	3.47031	3.39 × 10^−4^	3.36832	4.28 × 10^−4^
7	1.00 × 10^−7^	6.12247	7.54 × 10^−7^	6.39178	4.06 × 10^−7^	6.61598	2.42 × 10^−7^
4.056	8.79 × 10^−5^	4.19624	6.36 × 10^−5^	3.652	2.23 × 10^−4^	4.0149	9.66 × 10^−5^
6.507	3.11 × 10^−7^	6.16116	6.90 × 10^−7^	6.51421	3.06 × 10^−7^	6.59264	2.55 × 10^−7^
6.507	3.11 × 10^−7^	6.02982	9.34 × 10^−7^	6.07775	8.36 × 10^−7^	6.15757	6.96 × 10^−7^
6.183	6.56 × 10^−7^	5.75642	1.75 × 10^−6^	5.79935	1.59 × 10^−6^	6.14927	7.09 × 10^−7^
6.969	1.07 × 10^−7^	6.9807	1.05 × 10^−7^	7.09296	8.07 × 10^−8^	6.88214	1.31 × 10^−7^
6.969	1.07 × 10^−7^	7.07943	8.33 × 10^−8^	7.00226	9.95 × 10^−8^	7.0122	9.72 × 10^−8^
4.797	1.60 × 10^−5^	5.33815	4.59 × 10^−6^	5.1137	7.70 × 10^−6^	5.03788	9.16 × 10^−6^
6.373	4.24 × 10^−7^	6.43448	3.68 × 10^−7^	6.72149	1.90 × 10^−7^	6.38544	4.12 × 10^−7^
6.373	4.24 × 10^−7^	6.18816	6.48 × 10^−7^	6.27232	5.34 × 10^−7^	6.01018	9.77 × 10^−7^
4.373	4.24 × 10^−5^	5.18561	6.52 × 10^−6^	4.11995	7.59 × 10^−5^	4.0249	9.44 × 10^−5^
4.373	4.24 × 10^−5^	5.71049	1.95 × 10^−6^	4.55697	2.77 × 10^−5^	4.44409	3.60 × 10^−5^
8.684	2.07 × 10^−9^	7.98265	1.04 × 10^−8^	8.86923	1.35 × 10^−9^	9.06253	8.66 × 10^−10^
8.684	2.07 × 10^−9^	7.62067	2.40 × 10^−8^	8.34427	4.53 × 10^−9^	8.81938	1.52 × 10^−9^
6.699	2.00 × 10^−7^	7.01552	9.65 × 10^−8^	6.80757	1.56 × 10^−7^	6.96906	1.07 × 10^−7^
6.699	2.00 × 10^−7^	6.7277	1.87 × 10^−7^	6.68683	2.06 × 10^−7^	6.83044	1.48 × 10^−7^
6	1.00 × 10^−6^	6.23644	5.80 × 10^−7^	5.90262	1.25 × 10^−6^	6.01238	9.72 × 10^−7^
6	1.00 × 10^−6^	7.01502	9.66 × 10^−8^	6.95673	1.10 × 10^−7^	6.91073	1.23 × 10^−7^
7	1.00 × 10^−7^	7.05387	8.83 × 10^−8^	6.71729	1.92 × 10^−7^	6.81745	1.52 × 10^−7^
7	1.00 × 10^−7^	6.93387	1.16 × 10^−7^	6.72269	1.89 × 10^−7^	7.00805	9.82 × 10^−8^
6	1.00 × 10^−6^	6.43509	3.67 × 10^−7^	6.10145	7.92 × 10^−7^	5.93209	1.17 × 10^−6^
6	1.00 × 10^−6^	6.667	2.15 × 10^−7^	6.2289	5.90 × 10^−7^	0.16779	6.80 × 10^−1^
6.699	2.00 × 10^−7^	6.68174	2.08 × 10^−7^	6.54365	2.86 × 10^−7^	6.73002	1.86 × 10^−7^
6.699	2.00 × 10^−7^	6.5807	2.63 × 10^−7^	6.31008	4.90 × 10^−7^	6.40679	3.92 × 10^−7^
7	1.00 × 10^−7^	5.97456	1.06 × 10^−6^	6.02123	9.52 × 10^−7^	6.00469	9.89 × 10^−7^
7	1.00 × 10^−7^	6.72661	1.88 × 10^−7^	7.15328	7.03 × 10^−8^	6.99133	1.02 × 10^−7^
6.699	2.00 × 10^−7^	6.9814	1.04 × 10^−7^	6.9427	1.14 × 10^−7^	6.9744	1.06 × 10^−7^
6.699	2.00 × 10^−7^	6.69806	2.00 × 10^−7^	6.65445	2.22 × 10^−7^	6.8252	1.50 × 10^−7^
6	1.00 × 10^−6^	6.24434	5.70 × 10^−7^	6.14196	7.21 × 10^−7^	6.169	6.78 × 10^−7^
6	1.00 × 10^−6^	5.76522	1.72 × 10^−6^	5.61604	2.42 × 10^−6^	6.00794	9.82 × 10^−7^
6.63	2.34 × 10^−7^	6.67603	2.11 × 10^−7^	6.83685	1.46 × 10^−7^	6.69395	2.02 × 10^−7^
6.63	2.34 × 10^−7^	6.36607	4.30 × 10^−7^	6.59184	2.56 × 10^−7^	6.54213	2.87 × 10^−7^
6.522	3.01 × 10^−7^	6.84414	1.43 × 10^−7^	6.7813	1.65 × 10^−7^	6.45553	3.50 × 10^−7^
6.522	3.01 × 10^−7^	6.72945	1.86 × 10^−7^	6.77693	1.67 × 10^−7^	6.58823	2.58 × 10^−7^
6.398	4.00 × 10^−7^	6.75476	1.76 × 10^−7^	6.62133	2.39 × 10^−7^	6.53722	2.90 × 10^−7^
6.398	4.00 × 10^−7^	6.42081	3.79 × 10^−7^	6.28066	5.24 × 10^−7^	6.57633	2.65 × 10^−7^
6.699	2.00 × 10^−7^	6.06396	8.63 × 10^−7^	6.07507	8.41 × 10^−7^	6.08148	8.29 × 10^−7^
6.699	2.00 × 10^−7^	6.45688	3.49 × 10^−7^	6.43992	3.63 × 10^−7^	6.30798	4.92 × 10^−7^
6.301	5.00 × 10^−7^	6.72526	1.88 × 10^−7^	7.26976	5.37 × 10^−8^	7.07778	8.36 × 10^−8^
6.301	5.00 × 10^−7^	6.3286	4.69 × 10^−7^	6.77081	1.70 × 10^−7^	6.78189	1.65 × 10^−7^
7.456	3.50 × 10^−8^	6.69501	2.02 × 10^−7^	6.90677	1.24 × 10^−7^	7.07044	8.50 × 10^−8^
7.456	3.50 × 10^−8^	5.93925	1.15 × 10^−6^	5.86596	1.36 × 10^−6^	5.92629	1.18 × 10^−6^
6.523	3.00 × 10^−7^	6.98627	1.03 × 10^−7^	7.18012	6.61 × 10^−8^	6.9041	1.25 × 10^−7^
6.523	3.00 × 10^−7^	6.7924	1.61 × 10^−7^	6.80761	1.56 × 10^−7^	6.45073	3.54 × 10^−7^
6.699	2.00 × 10^−7^	7.05743	8.76 × 10^−8^	6.86573	1.36 × 10^−7^	6.93166	1.17 × 10^−7^
6.699	2.00 × 10^−7^	6.80905	1.55 × 10^−7^	6.3767	4.20 × 10^−7^	6.70095	1.99 × 10^−7^
6.699	2.00 × 10^−7^	6.55522	2.78 × 10^−7^	6.68693	2.06 × 10^−7^	6.39142	4.06 × 10^−7^
6.699	2.00 × 10^−7^	6.25738	5.53 × 10^−7^	6.34931	4.47 × 10^−7^	6.25896	5.51 × 10^−7^

**Table 5:** Model Activity PLS Factors.

#Factors	SD	R^2^	R^2^ CV	R^2^ Scramble	Stability	F	*p*	RMSE	Q^2^	Pearson-r
1	0.5711	0.7961	0.5178	0.5696	0.842	210.8	2.73 × 10^−20^	1.03	0.2665	0.5408
2	0.356	0.9223	0.5415	0.8179	0.701	314.4	4.00 × 10^−30^	1.03	0.2682	0.5625
3	0.2813	0.9524	0.532	0.9157	0.644	346.5	2.42 × 10^−34^	1.04	0.2519	0.5382

**Table 6:** Molecular properties of selected compounds, including molecular weight (MW), various volume descriptors (L-V, G-V, V-V, E-V, GSK-V, M-V), density, lipophilicity (logD, logP), solubility (logS), quantitative estimate of drug-likeness (QED), hydrogen bond acceptors (nHA) and donors (nHD), rotatable bonds (nRot), topological polar surface area (TPSA), and Golden Triangle score.

S/N	MW	L-V	G-V	V-V	E-V	GSK-V	M-V	Dense	logD	logS	logP	QED	nHA	nHD	nRot	TPSA	Golden Triangle
1	326.3	0	0	0	0	0	0	1.008	2.433	−3.510	2.974	0.701	6	1	6	85.97	0
2	340.33	0	0	0	0	0	0	0.998	2.499	−3.495	3.373	0.664	6	1	7	85.97	0
3	368.38	0	0	0	0	1	0	0.981	2.870	−3.711	4.018	0.572	6	1	9	85.97	0
4	396.43	0	0	1	0	1	1	0.966	3.109	−3.799	4.512	0.464	6	1	11	85.97	0
5	424.49	0	1	1	0	1	1	0.954	3.341	−3.869	5.112	0.355	6	1	13	85.97	0
6	340.33	0	0	0	0	0	0	0.999	2.532	−3.501	3.562	0.664	6	1	7	85.97	0
7	368.38	0	0	0	0	1	0	0.981	2.949	−3.721	4.341	0.572	6	1	9	85.97	0
8	312.27	0	0	0	0	0	0	1.020	2.157	−3.065	2.811	0.729	6	1	5	85.97	0
9	326.3	0	0	0	0	0	0	1.009	2.534	−3.805	3.122	0.701	6	1	6	85.97	0
10	340.33	0	0	0	0	0	0	0.999	2.553	−3.522	3.540	0.664	6	1	7	85.97	0
11	368.38	0	0	0	0	1	0	0.981	2.953	−3.767	4.223	0.572	6	1	9	85.97	0
12	396.43	0	0	1	0	1	1	0.967	3.154	−3.758	4.659	0.464	6	1	11	85.97	0
13	424.49	0	1	1	0	1	1	0.955	3.362	−3.817	5.162	0.355	6	1	13	85.97	0
14	384.45	0	0	0	0	1	0	0.998	3.396	−3.904	4.830	0.42	5	1	9	76.74	0
15	384.45	0	0	0	0	1	0	0.998	3.354	−3.878	4.827	0.42	5	1	9	76.74	0
16	368.45	0	0	0	0	1	1	0.979	3.578	−4.028	5.103	0.448	4	1	8	67.51	0
17	340.33	0	0	0	0	0	0	0.999	2.428	−3.377	3.282	0.664	6	1	7	85.97	0
18	368.38	0	0	0	0	0	0	0.981	2.830	−3.681	3.910	0.572	6	1	9	85.97	0
19	356.33	0	0	0	0	0	0	1.019	1.912	−2.492	1.976	0.669	7	2	7	106.2	0
20	414.36	0	0	1	1	1	0	1.038	1.862	−2.731	1.806	0.473	9	3	8	143.5	0
21	454.43	0	0	1	1	1	0	1.013	2.559	−3.904	3.388	0.311	9	3	10	143.5	0
22	454.43	0	0	1	1	1	0	1.013	2.612	−4.014	3.675	0.311	9	3	10	143.5	0
23	494.49	0	2	2	1	1	0	0.994	2.780	−4.289	4.040	0.254	9	3	12	143.5	0
24	456.44	0	0	1	1	1	0	1.012	2.741	−4.030	3.972	0.392	9	3	10	143.5	0
25	498.52	0	2	2	1	1	0	0.991	3.175	−4.705	5.173	0.314	9	3	12	143.5	0
26	398.41	0	0	0	0	0	0	0.992	2.753	−3.671	3.896	0.57	7	2	9	106.2	0
27	440.49	0	0	1	0	1	0	0.972	3.578	−4.268	5.089	0.46	7	2	11	106.2	0
28	396.39	0	0	0	0	0	0	0.994	2.592	−3.555	3.534	0.536	7	2	9	106.2	0
29	440.49	0	0	1	0	1	0	0.972	3.570	−4.220	5.089	0.46	7	2	11	106.2	0
30	512.55	1	2	1	1	1	0	0.985	3.476	−4.514	5.034	0.319	9	2	13	132.5	1
31	482.52	0	2	1	0	1	0	0.976	3.572	−4.400	5.123	0.364	8	2	12	123.27	0
32	546.61	1	4	1	0	1	1	0.964	4.002	−4.379	6.257	0.204	8	2	14	115.43	1
33	456.44	0	0	1	1	1	0	1.012	2.828	−4.368	4.197	0.392	9	3	10	143.5	0
34	368.38	0	0	0	0	1	0	0.981	2.953	−3.767	4.223	0.572	6	1	9	85.97	0
35	466.48	0	0	1	0	1	0	0.984	3.176	−4.178	4.799	0.223	8	2	12	123.27	0
36	384.38	0	0	0	0	0	0	1.001	2.496	−3.483	3.559	0.54	7	2	9	106.2	0
37	474.5	0	1	1	0	1	1	0.971	3.254	−3.772	5.111	0.263	7	1	12	95.2	0
38	398.41	0	0	0	0	1	0	0.992	3.116	−3.826	4.308	0.513	7	1	10	95.2	0
39	410.42	0	0	0	0	1	0	0.986	2.628	−3.570	3.409	0.392	7	1	10	103.04	0
40	465.5	0	0	1	0	1	0	0.977	2.849	−3.899	4.176	0.291	8	2	13	115.07	0
41	466.48	0	0	1	0	1	0	0.984	3.301	−4.414	4.986	0.223	8	2	12	123.27	0
42	450.48	0	0	1	0	1	0	0.968	3.413	−4.076	4.900	0.234	7	1	12	103.04	0
43	506.54	1	2	1	0	1	1	0.968	3.645	−4.656	5.720	0.167	8	2	14	123.27	1
44	426.42	0	0	0	0	1	0	1.003	2.658	−3.716	3.804	0.368	8	2	10	123.27	0
45	424.49	0	0	1	0	1	1	0.955	4.273	−4.815	6.370	0.424	6	1	11	85.97	0
46	424.49	0	0	1	0	1	1	0.955	4.258	−4.742	6.295	0.424	6	1	11	85.97	0
47	482.52	0	2	1	0	1	1	0.976	3.855	−4.734	6.452	0.268	8	2	12	123.27	0
48	486.55	0	2	1	0	1	1	0.963	3.228	−4.372	5.709	0.266	8	2	15	119.36	0
49	514.61	1	4	1	0	1	2	0.953	3.503	−4.505	6.294	0.201	8	2	17	119.36	1
50	542.66	1	4	1	1	1	2	0.944	3.680	−4.423	6.918	0.151	8	2	19	119.36	1
51	514.61	1	4	1	0	1	2	0.953	3.484	−4.586	6.095	0.201	8	2	17	119.36	1
52	542.66	1	4	1	1	1	2	0.944	3.718	−4.674	6.744	0.151	8	2	19	119.36	1
53	516.58	1	3	1	0	1	2	0.972	3.282	−4.201	5.011	0.228	9	2	17	128.59	1
54	544.63	1	3	1	0	1	2	0.962	3.504	−4.817	5.649	0.176	9	2	19	128.59	1
55	572.69	1	4	1	1	1	2	0.953	3.583	−4.974	6.166	0.133	9	2	21	128.59	1
56	560.63	1	3	1	1	1	2	0.975	3.281	−4.098	4.920	0.174	10	2	20	137.82	1
57	588.69	1	3	1	1	1	2	0.966	3.511	−4.626	5.513	0.135	10	2	22	137.82	1
58	604.69	2	3	2	1	1	3	0.978	3.322	−4.069	4.886	0.135	11	2	23	147.05	1
59	544.63	1	3	1	0	1	2	0.962	3.388	−4.577	5.808	0.176	9	2	19	128.59	1
60	572.69	1	4	1	1	1	2	0.953	3.493	−4.862	6.251	0.133	9	2	21	128.59	1
61	572.69	1	4	1	1	1	2	0.953	3.472	−4.865	6.179	0.133	9	2	21	128.59	1
62	402.44	0	0	1	0	1	1	0.975	2.910	−4.032	4.784	0.41	7	2	12	102.29	0
63	498.52	0	2	2	1	1	0	0.991	3.175	−4.705	5.173	0.314	9	3	12	143.5	0
64	452.5	0	0	1	0	1	1	0.967	3.019	−4.600	5.491	0.294	7	2	12	102.29	0
65	494.53	0	2	1	0	1	1	0.972	3.247	−4.738	5.255	0.245	8	2	13	119.36	0
66	522.59	1	4	1	1	1	1	0.962	3.459	−4.961	5.778	0.184	8	2	15	119.36	1
67	480.55	0	3	1	0	1	1	0.956	3.214	−4.714	5.545	0.222	7	2	14	102.29	0
68	522.59	1	4	1	1	1	1	0.962	3.439	−4.921	5.583	0.184	8	2	15	119.36	1

Lipinski violations (L-V); Ghose violations (G-V); Veber violations (V-V); Egan violations (E-V); Muegge violations (M-V); GSK 4/400 rule violations (GSK-V); S/N: Serial Number/Compound Number.

**Table 7:** Summary of selected lead compounds based on QSAR model predictions, pIC_50_ values, and molecular docking analysis. The table highlights the predictive performance from QSAR models, binding affinities with the CysLT_1_ receptor, and final remarks regarding each compound’s suitability for further antioxidant evaluation.

Compd. No.	pIC_50_	Binding Energy (kcal/mol)	QSAR Performance	Docking Interpretation	Final Remark
**25**	7.99	−9.5	Predicted accurately; within high-activity range	Strong binding affinity; stable interactions	Top lead candidate; synthesized and tested
**41**	6.96	−10	Good QSAR prediction; favorable model alignment	Strong binding score; strong receptor interactions	Promising dual-acting molecule; synthesized and tested
**47**	8.68	−9.4	Very high predicted activity; QSAR consensus supported	Strong binding score; strong receptor interactions	Most potent QSAR hit; prioritized for future synthesis
Zafirlukast	8.72	−13.2	Reference [37]		

## Data Availability

The authors affirm that the data supporting the findings of this study are available within the article.

## Supplementary Materials

The following supporting information can be downloaded at: https://www.mdpi.com/article/10.3390/ph19040600/s1. Table S1, providing molecular descriptors and Canonical SMILES of the compounds; Table S2, which contains the molecular descriptor correlation matrix used for QSAR model development; and Table S3, detailing the actual and predicted IC_50_ values for 68 compounds. Additionally, Table S4 lists the top 10 prioritized compounds based on an integrated evaluation of experimental pIC_50_, QSAR-predicted pIC_50_ (ANN-LM model), and molecular docking binding energy (BE). Finally, Figure S1 presents a comparison of actual IC_50_ values (black dashed line) with predicted IC_50_ values derived from multiple models.

## References

[1] Liu M., Yokomizo T. (2015). The Role of Leukotrienes in Allergic Diseases. Allergol. Int..

[2] Ogawa Y., Calhoun W.J. (2006). The Role of Leukotrienes in Airway Inflammation. J. Allergy Clin. Immunol..

[3] Sasaki F., Yokomizo T. (2019). The Leukotriene Receptors as Therapeutic Targets of Inflammatory Diseases. Int. Immunol..

[4] Brocklehurst W.E. (1963). “SRS-A” The Slow Reacting Substance of Anaphylaxis. Biochem. Pharmacol..

[5] Lee M., Boyce J.A., Barrett N.A. (2025). Cysteinyl Leukotrienes in Allergic Inflammation. Annu. Rev. Pathol. Mech. Dis..

[6] Lewis R.A., Austen K.F., Drazen J.M., Clark D.A., Marfat A., Corey E.J. (1980). Slow Reacting Substances of Anaphylaxis: Identification of Leukotrienes C-1 and D from Human and Rat Sources. Proc. Natl. Acad. Sci. USA.

[7] Bernstein P.R. (1998). Chemistry and Structure–Activity Relationships of Leukotriene Receptor Antagonists. Am. J. Respir. Crit. Care Med..

[8] LeMahieu R.A., Carson M., Han R.-J., Nason W.C., O’Donnell M., Brown D.L., Crowley H.J., Welton A.F. (1987). Substituted (Aryloxy)Alkanoic Acids as Antagonists of Slow-Reacting Substance of Anaphylaxis. J. Med. Chem..

[9] Griera R., Armengol M., Reyes A., Alvarez M., Palomer A., Cabré F., Pascual J., Garcia M.L., Mauleón D. (1997). Synthesis and Pharmacological Evaluation of New CysLT1 Receptor Antagonists. Eur. J. Med. Chem..

[10] Itadani S., Takahashi S., Ima M., Sekiguchi T., Fujita M., Nakayama Y., Takeuchi J. (2014). Discovery of Highly Potent Dual CysLT_1_ and CysLT_2_ Antagonist. ACS Med. Chem. Lett..

[11] Tilley J.W., Levitan P., Welton A.F., Crowley H.J. (1983). Antagonists of Slow-Reacting Substance of Anaphylaxis. 1. Pyrido[2,1-b]Quinazolinecarboxylic Acid Derivatives. J. Med. Chem..

[12] Lewis R.A., Wood D. (2014). Modern 2D QSAR for Drug Discovery. WIREs Comput. Mol. Sci..

[13] Khelfa N., Belaidi S., Abchir O., Yamari I., Chtita S., Samadi A., Al-Mogren M.M., Hochlaf M. (2024). Combined 3D-QSAR, Molecular Docking, ADMET, and Drug-Likeness Scoring of Novel Diaminodihydrotriazines as Potential Antimalarial Agents. Sci. Afr..

[14] Zhuo W., Lian Z., Bai W., Chen Y., Xia H. (2023). 3D- and 2D-QSAR Models’ Study and Molecular Docking of Novel Nitrogen-Mustard Compounds for Osteosarcoma. Front. Mol. Biosci..

[15] Melo-Filho C., Braga R., Andrade C. (2014). 3D-QSAR Approaches in Drug Design: Perspectives to Generate Reliable CoMFA Models. Curr. Comput. Aided-Drug Des..

[16] Pinzi L., Rastelli G. (2019). Molecular Docking: Shifting Paradigms in Drug Discovery. Int. J. Mol. Sci..

[17] Ibrahim M.T., Uzairu A. (2023). 2D-QSAR, Molecular Docking, Drug-Likeness, and ADMET/Pharmacokinetic Predictions of Some Non-Small Cell Lung Cancer Therapeutic Agents. J. Taibah Univ. Med. Sci..

[18] Yu Y., Dong H., Peng Y., Welsh W.J. (2021). QSAR-Based Computational Approaches to Accelerate the Discovery of Sigma-2 Receptor (S2R) Ligands as Therapeutic Drugs. Molecules.

[19] Wang H., Qin Z., Yan A. (2021). Classification Models and SAR Analysis on CysLT1 Receptor Antagonists Using Machine Learning Algorithms. Mol. Divers..

[20] Shahlaei M. (2013). Descriptor Selection Methods in Quantitative Structure–Activity Relationship Studies: A Review Study. Chem. Rev..

[21] Grisoni F., Ballabio D., Todeschini R., Consonni V. (2018). Molecular Descriptors for Structure–Activity Applications: A Hands-On Approach. Computational Toxicology: Methods and Protocols.

[22] Sepehri B., Kohnehpoushi M., Ghavami R. (2022). High Predictive QSAR Models for Predicting the SARS Coronavirus Main Protease Inhibition Activity of Ketone-Based Covalent Inhibitors. J. Iran. Chem. Soc..

[23] Shameera Ahamed T.K., Rajan V.K., Muraleedharan K. (2019). QSAR Modeling of Benzoquinone Derivatives as 5-Lipoxygenase Inhibitors. Food Sci. Hum. Wellness.

[24] Niemi J.B., Niemi G.J., Basak S.C., Restrepo G., Villaveces J.L. (2015). Advances in Mathematical Chemistry and Applications.

[25] Denizhan O. (2024). Comparison of Different Supervised Learning Algorithms for Position Analysis of the Slider-Crank Mechanism. Alex. Eng. J..

[26] Yanis M., Budiman A.Y., Mohruni A.S., Sharif S., Suhaimi M.A., Dwipayana H. (2023). Levenberg-Marquardt, Bayesian-Regularization, and Scaled Conjugate Gradient Algorithms for Predicting Surface Roughness Accuracy on Side Milling AISI 1045. AIP Conf. Proc..

[27] Hadni H., Elhallaoui M. (2020). 2D and 3D-QSAR, Molecular Docking and ADMET Properties in Silico Studies of Azaaurones as Antimalarial Agents. New J. Chem..

[28] Wu X., Gong J., Ren S., Tan F., Wang Y., Zhao H. (2024). A Machine Learning-Based QSAR Model Reveals Important Molecular Features for Understanding the Potential Inhibition Mechanism of Ionic Liquids to Acetylcholinesterase. Sci. Total Environ..

[29] Wu F., Zhou Y., Li L., Shen X., Chen G., Wang X., Liang X., Tan M., Huang Z. (2020). Computational Approaches in Preclinical Studies on Drug Discovery and Development. Front. Chem..

[30] Tian S., Wang J., Li Y., Li D., Xu L., Hou T. (2015). The Application of in Silico Drug-Likeness Predictions in Pharmaceutical Research. Adv. Drug Deliv. Rev..

[31] Komura H., Watanabe R., Mizuguchi K. (2023). The Trends and Future Prospective of In Silico Models from the Viewpoint of ADME Evaluation in Drug Discovery. Pharmaceutics.

[32] Yusof I., Shah F., Hashimoto T., Segall M.D., Greene N. (2014). Finding the Rules for Successful Drug Optimisation. Drug Discov. Today.

[33] Kralj S., Jukič M., Bren U. (2023). Molecular Filters in Medicinal Chemistry. Encyclopedia.

[34] Vilar S., Costanzi S. (2012). Predicting the Biological Activities Through QSAR Analysis and Docking-Based Scoring. Membrane Protein Structure and Dynamics: Methods and Protocols.

[35] Ramírez D., Caballero J. (2016). Is It Reliable to Use Common Molecular Docking Methods for Comparing the Binding Affinities of Enantiomer Pairs for Their Protein Target?. Int. J. Mol. Sci..

[36] Guedes I.A., de Magalhães C.S., Dardenne L.E. (2014). Receptor–Ligand Molecular Docking. Biophys. Rev..

[37] Figueroa E.E., Kramer M., Strange K., Denton J.S. (2019). CysLT1 Receptor Antagonists Pranlukast and Zafirlukast Inhibit LRRC8-Mediated Volume Regulated Anion Channels Independently of the Receptor. Am. J. Physiol.-Cell Physiol..

[38] Rajasekhar D., Srinivasulu D., Sridhar C., Kumar G.V.N., Ramesh P. (2016). Synthesis, Spectral Characterization and Antioxidant Activity of Novel Zafirlukast Sulfonyl Derivatives. J. Chin. Chem. Soc..

[39] Wang J., Mochizuki H., Todokoro M., Arakawa H., Morikawa A. (2008). Does Leukotriene Affect Intracellular Glutathione Redox State in Cultured Human Airway Epithelial Cells?. Antioxid. Redox Signal..

[40] El-Boghdady N.A., Abdeltawab N.F., Nooh M.M. (2017). Resveratrol and Montelukast Alleviate Paraquat-Induced Hepatic Injury in Mice: Modulation of Oxidative Stress, Inflammation, and Apoptosis. Oxid. Med. Cell. Longev..

[41] Lee Y.A., Shin M.H. (2024). CysLT Receptor-Mediated NOX2 Activation Is Required for IL-8 Production in HMC-1 Cells Induced by *Trichomonas vaginalis*-Derived Secretory Products. Parasites Hosts Dis..

[42] Costa A.S., Martins J.P.A., de Melo E.B. (2022). SMILES-Based 2D-QSAR and Similarity Search for Identification of Potential New Scaffolds for Development of SARS-CoV-2 MPRO Inhibitors. Struct. Chem..

[43] Dong J., Yao Z.-J., Zhu M.-F., Wang N.-N., Lu B., Chen A.F., Lu A.-P., Miao H., Zeng W.-B., Cao D.-S. (2017). ChemSAR: An Online Pipelining Platform for Molecular SAR Modeling. J. Cheminform..

[44] Nguyen H.D., Kim M.-S. (2023). Identification of Promising Inhibitory Heterocyclic Compounds against Acetylcholinesterase Using QSAR, ADMET, Biological Activity, and Molecular Docking. Comput. Biol. Chem..

[45] Rosell-Hidalgo A., Moore A.L., Ghafourian T. (2023). Prediction of Drug-Induced Mitochondrial Dysfunction Using Succinate-Cytochrome c Reductase Activity, QSAR and Molecular Docking. Toxicology.

[46] Damarla R. (2022). Enhancement of Drug Discovery with Machine Learning Clustering Algorithms. J. High Sch. Sci..

[47] Andrada M.F., Vega-Hissi E.G., Estrada M.R., Garro Martinez J.C. (2015). Application of K-Means Clustering, Linear Discriminant Analysis and Multivariate Linear Regression for the Development of a Predictive QSAR Model on 5-Lipoxygenase Inhibitors. Chemom. Intell. Lab. Syst..

[48] Gramatica P., Cassani S., Chirico N. (2014). QSARINS-chem: Insubria Datasets and New QSAR/QSPR Models for Environmental Pollutants in QSARINS. J. Comput. Chem..

[49] Ventura C., Latino D.A.R.S., Martins F. (2013). Comparison of Multiple Linear Regressions and Neural Networks Based QSAR Models for the Design of New Antitubercular Compounds. Eur. J. Med. Chem..

[50] Daoui O., Elkhattabi S., Chtita S., Elkhalabi R., Zgou H., Benjelloun A.T. (2021). QSAR, Molecular Docking and ADMET Properties in Silico Studies of Novel 4,5,6,7-Tetrahydrobenzo[D]-Thiazol-2-Yl Derivatives Derived from Dimedone as Potent Anti-Tumor Agents through Inhibition of C-Met Receptor Tyrosine Kinase. Heliyon.

[51] Nguyen H.D. (2024). In Silico Identification of Novel Heterocyclic Compounds Combats Alzheimer’s Disease through Inhibition of Butyrylcholinesterase Enzymatic Activity. J. Biomol. Struct. Dyn..

[52] Shi Y. (2021). Support Vector Regression-Based QSAR Models for Prediction of Antioxidant Activity of Phenolic Compounds. Sci. Rep..

[53] Hunter J.D. (2007). Matplotlib: A 2D Graphics Environment. Comput. Sci. Eng..

[54] (2023). Issue Information. WIREs Comput. Mol. Sci..

[55] Probst D., Reymond J.-L. (2020). Visualization of Very Large High-Dimensional Data Sets as Minimum Spanning Trees. J. Cheminform..

[56] Kuwahara H., Gao X. (2021). Analysis of the Effects of Related Fingerprints on Molecular Similarity Using an Eigenvalue Entropy Approach. J. Cheminform..

[57] Mellor C.L., Marchese Robinson R.L., Benigni R., Ebbrell D., Enoch S.J., Firman J.W., Madden J.C., Pawar G., Yang C., Cronin M.T.D. (2019). Molecular Fingerprint-Derived Similarity Measures for Toxicological Read-across: Recommendations for Optimal Use. Regul. Toxicol. Pharmacol..

[58] Heng S.Y., Ridwan W.M., Kumar P., Ahmed A.N., Fai C.M., Birima A.H., El-Shafie A. (2022). Artificial Neural Network Model with Different Backpropagation Algorithms and Meteorological Data for Solar Radiation Prediction. Sci. Rep..

[59] Gedeck P., Rohde B., Bartels C. (2006). QSAR − How Good Is It in Practice? Comparison of Descriptor Sets on an Unbiased Cross Section of Corporate Data Sets. J. Chem. Inf. Model..

[60] Dixon S.L., Smondyrev A.M., Rao S.N. (2006). PHASE: A Novel Approach to Pharmacophore Modeling and 3D Database Searching. Chem. Biol. Drug Des..

[61] Schrödinger, LLC (2023). Schrödinger Release 2023-1.

[62] Pedregosa F., Varoquaux G., Gramfort A., Michel V., Thirion B., Grisel O., Blondel M., Prettenhofer P., Weiss R., Dubourg V. (2011). Scikit-Learn: Machine Learning in Python. J. Mach. Learn. Res..

[63] McKinney W. (2010). Data Structures for Statistical Computing in Python. Scipy.

[64] Schneider G. (2012). From Theory to Bench Experiment by Computer-Assisted Drug Design. Chimia.

[65] Daina A., Michielin O., Zoete V. (2019). SwissTargetPrediction: Updated Data and New Features for Efficient Prediction of Protein Targets of Small Molecules. Nucleic Acids Res..

[66] Xiong G., Wu Z., Yi J., Fu L., Yang Z., Hsieh C., Yin M., Zeng X., Wu C., Lu A. (2021). ADMETlab 2.0: An Integrated Online Platform for Accurate and Comprehensive Predictions of ADMET Properties. Nucleic Acids Res..

[67] Pires D.E., Blundell T.L., Ascher D.B. (2015). pkCSM: Predicting small-molecule pharmacokinetic and toxicity properties using graph-based signatures. J. Med. Chem..

[68] Kabier M., Gambacorta N., Trisciuzzi D., Kumar S., Nicolotti O., Mathew B. (2024). MzDOCK: A Free Ready-to-Use GUI-Based Pipeline for Molecular Docking Simulations Simulations. J. Comput. Chem..

[69] BIOVIA (2016). Dassault Systèmes, Discovery Studio Modeling Environment, Release 2016, San Diego: Dassault Systèmes. https://www.3ds.com/products/biovia/discovery-studio.

[70] Brańka A.C. (2000). Nosé-Hoover Chain Method for Nonequilibrium Molecular Dynamics Simulation. Phys. Rev. E.

[71] Syameera N.A., Kaewdaungdee S., Tajuddin S.N., Tanee T., Sudmoon R., Chaveerach A., Lee S.Y. (2024). Effects of Heat Treatment on the Chemical Composition, Antioxidant Activity, and Toxicity of Agarwood Oil. J. King Saud Univ. Sci..

[72] Ahmad F., Alam M.J., Alam M., Azaz S., Parveen M., Park S., Ahmad S. (2018). Synthesis, Spectroscopic, Computational (DFT/B3LYP), AChE Inhibition and Antioxidant Studies of Imidazole Derivative. J. Mol. Struct..

