# Supplementary material for: Integrated QSAR, Molecular Docking, ADMET Profiling, and Antioxidant Evaluation of Substituted Chromone and Aryloxyalkanoic Acid Derivatives as Potential CysLT1 Receptor Antagonists
Source: Pharmaceuticals (Basel). 2026 Apr 8;19(4):600. doi: 10.3390/ph19040600 (PMC13119130; doi:10.3390/ph19040600)
Supplement: Supplementary file 1 [file pharmaceuticals-19-00600-s001.zip › pharmaceuticals-4201731-supplementary.pdf]

Supplementary Information (SI)

Integrated QSAR, Molecular Docking, ADMET Profiling, and Antioxidant Evaluation of Substituted Chromone and Aryloxyalkanoic Acid Derivatives as Potential $CysLT_1$ Receptor Antagonists

Mahboob Alam

Table S1: Molecular descriptors and bioactivity data for QSAR analysis, including Canonical SMILES, Formula, Molecular Weight (MW), pIC50, Smax33, ndonr, Smax15, MRVSA6, Qindex, EstateVSA1, S17, and PEOEVSA1.

Molecule	Canonical SMILES	Formula	MW	pIC50	Smax33	ndonr	Smax15	MRVSA6	Qindex	EstateVSA1	S17	PEOEVSA1
Molecule 1	OC(=O)c1cc(=O)c2c(o1)cccc2OCCOc1ccccc1	$C_{18}H_{14}O_6$	326.3	3.513617074	8.939	1	-1.295	70.581	16	17.576	0.65	4.795
Molecule 2	OC(=O)c1cc(=O)c2c(o1)cccc2OCCCOc1ccccc1	$C_{19}H_{16}O_6$	340.33	3.987832189	8.955	1	-1.289	70.581	16	10.969	0.753	4.795
Molecule 3	OC(=O)c1cc(=O)c2c(o1)cccc2OCCCCCOc1ccccc1	$C_{21}H_{20}O_6$	368.38	4.323257995	8.981	1	-1.28	70.581	16	16.729	0.874	4.795
Molecule 4	OC(=O)c1cc(=O)c2c(o1)cccc2OCCCCCCCOc1ccccc1	$C_{23}H_{24}O_6$	396.43	5.695076525	9.002	1	-1.274	70.581	16	16.729	0.941	4.795
Molecule 5	OC(=O)c1cc(=O)c2c(o1)cccc2OCCCCCCCCCOc1ccccc1	$C_{25}H_{28}O_6$	424.49	4.849716213	9.02	1	-1.269	70.581	16	16.729	0.983	4.795
Molecule 6	OC(=O)c1cc(=O)c2c(o1)ccc(c2)OCCCOc1ccccc1	$C_{19}H_{16}O_6$	340.33	5.03358968	8.903	1	-1.28	70.581	16	16.729	0.937	4.795
Molecule 7	OC(=O)c1cc(=O)c2c(o1)ccc(c2)OCCCCCOc1ccccc1	$C_{21}H_{20}O_6$	368.38	4.975231436	8.927	1	-1.273	70.581	16	11.343	1.048	4.795
Molecule 8	OC(=O)c1cc(=O)c2c(o1)ccc(c2)OCOc1ccccc1	$C_{17}H_{12}O_6$	312.27	4.674986328	8.874	1	-1.291	70.581	16	17.762	0.686	4.795
Molecule 9	OC(=O)c1cc(=O)c2c(o1)cc(cc2)OCCOc1ccccc1	$C_{18}H_{14}O_6$	326.3	4.337525815	8.939	1	-1.295	70.581	16	5.583	0.804	4.795
Molecule 10	OC(=O)c1cc(=O)c2c(o1)cc(cc2)OCCCOc1ccccc1	$C_{19}H_{16}O_6$	340.33	5.385772198	8.955	1	-1.289	70.581	16	5.583	0.903	4.795
Molecule 11	OC(=O)c1cc(=O)c2c(o1)cc(cc2)OCCCCCOc1ccccc1	$C_{21}H_{20}O_6$	368.38	5.964236052	8.981	1	-1.28	70.581	16	11.343	1.018	4.795
Molecule 12	OC(=O)c1oc2cc(OCCCCCCCOc3ccccc3)ccc2c(=O)c1	$C_{23}H_{24}O_6$	396.43	5.342894007	9.002	1	-1.274	70.581	16	16.772	1.081	4.795
Molecule 13	OC(=O)c1cc(=O)c2c(o1)cc(cc2)OCCCCCCCCCOc1ccccc1	$C_{25}H_{28}O_6$	424.49	5.372594959	9.02	1	-1.269	70.581	16	16.772	1.122	4.795
Molecule 14	OC(=O)c1cc(=O)c2c(o1)cc(cc2)OCCCCCSc1ccccc1	$C_{21}H_{20}O_5S$	384.45	5.982779874	8.989	1	-1.27	70.581	16	16.772	1.472	4.795
Molecule 15	OC(=O)c1cc(=O)c2c(o1)cc(cc2)SCCCCCOc1ccccc1	$C_{21}H_{20}O_5S$	384.45	6.107718611	9.007	1	-1.246	70.581	16	11.189	1.507	4.795
Molecule 16	OC(=O)c1cc(=O)c2c(o1)cc(cc2)SCCCCCc1ccccc1	$C_{21}H_{20}O_4S$	368.45	5.788227311	9.006	1	-1.236	76.144	16	11.189	2.024	4.795
Molecule 17	OC(=O)c1cc(=O)c2c(o1)c(OCCCOc1ccccc1)ccc2	$C_{19}H_{16}O_6$	340.33	3.832930229	9.024	1	-1.301	70.581	16	10.969	0.697	4.795

Molecule 18	OC(=O)c1cc(=O)c2c(o1)c(OCCCCCOc1ccccc1)ccc2	$C_{21}H_{20}O_6$	368.38	4.612053534	9.053	1	-1.29	70.581	16	5.583	0.823	4.795
Molecule 19	OC(COc1ccccc1)COc1ccc2c(c1)oc(cc2=O)C(=O)O	$C_{19}H_{16}O_7$	356.33	4.706754348	9.945	2	-1.33	70.581	17	24.183	0.526	4.795
Molecule 20	OC(COc1ccc(c(c1)O)C(=O)C)COc1ccc2c(c1)oc(cc2=O)C(=O)O	$C_{21}H_{18}O_9$	414.36	4.170219793	10.04	3	-0.283	64.012	20	52.778	-0.012	9.589
Molecule 21	C=CCc1c(OCC(COc2ccc3c(c2)oc(cc3=O)C(=O)O)O)ccc(c1O)C(=O)C	$C_{24}H_{22}O_9$	454.43	6.657466995	10.345	3	-0.292	76.164	21	53.45	0.409	9.589
Molecule 22	C=CCc1c(OCC(COc2ccc(c(c2)O)C(=O)C)O)ccc2c1oc(cc2=O)C(=O)O	$C_{24}H_{22}O_9$	454.43	5.138953054	10.241	3	-0.287	76.164	21	59.199	0.407	9.589
Molecule 23	C=CCc1c(OCC(COc2ccc(c(c2CC=C)O)C(=O)C)O)ccc2c1oc(cc2=O)C(=O)O	$C_{27}H_{26}O_9$	494.49	6.995187511	10.442	3	-0.296	88.316	22	65.62	0.815	9.589
Molecule 24	CCCc1c(OCC(COc2ccc3c(c2)oc(cc3=O)C(=O)O)O)ccc(c1O)C(=O)C	$C_{24}H_{24}O_9$	456.44	6.756293709	10.392	3	-0.258	63.509	21	47.029	0.737	9.589
Molecule 25	CCCc1c(OCC(COc2ccc(c(c2CCC)O)C(=O)C)O)ccc2c1oc(cc2=O)C(=O)O	$C_{27}H_{30}O_9$	498.52	7.998712577	10.482	3	-0.255	63.006	22	41.279	1.42	9.589
Molecule 26	CCCc1ccccc1OCC(COc1ccc2c(c1)oc(cc2=O)C(=O)O)O	$C_{22}H_{22}O_7$	398.41	5.299300235	10.166	2	-1.328	70.078	18	24.183	1.719	4.795
Molecule 27	CCCc1c(OCC(COc2ccccc2CCC)O)ccc2c1oc(cc2=O)C(=O)O	$C_{25}H_{28}O_7$	440.49	7.000483378	10.387	2	-1.311	69.575	19	18.797	2.458	4.795
Molecule 28	C=CCc1ccccc1OCC(COc1ccc2c(c1)oc(cc2=O)C(=O)O)O	C22H20O7	396.39	5.342850185	10.146	2	-1.334	82.733	18	24.183	1.509	4.795
Molecule 29	CCCc1cc2c(cc1OCC(COc1ccccc1CCC)O)oc(cc2=O)C(=O)O	$C_{25}H_{28}O_7$	440.49	5.439816071	10.387	2	-1.319	69.575	19	18.797	2.629	4.795
Molecule 30	CCCc1c(OCC(COc2ccc(c(c2CCC)OC)C(=O)C)O)ccc2c1oc(cc2=O)C(=O)O	$C_{28}H_{32}O_9$	512.55	4.056523724	10.57	2	-0.112	63.006	22	29.966	2.716	9.589
Molecule 31	CCCc1c(OCC(COc2ccc(cc2CCC)C(=O)C)O)ccc2c1oc(cc2=O)C(=O)O	$C_{27}H_{30}O_8$	482.52	6.507424061	10.475	2	-0.021	69.072	21	29.966	2.657	9.589
Molecule 32	CCCc1c(OCC(COc2ccc3c(c2CCC)oc(cc3=O)C(=O)O)O)cccc1OCc1ccccc1	$C_{32}H_{34}O_8$	546.61	5.737677573	10.677	2	-1.322	99.404	24	24.183	3.974	4.795
Molecule 33	CCCc1c(OCC(COc2cccc(c2C(=O)C)O)O)ccc2c1oc(cc2=O)C(=O)O	$C_{24}H_{24}O_9$	456.44	6.182262442	10.317	3	-0.378	63.509	21	47.029	0.346	9.589
Molecule 34	OC(=O)c1cc(=O)c2c(o1)cc(cc2)OCCCCCOc1ccccc1	$C_{21}H_{20}O_6$	368.38	5.932827588	8.981	1	-1.28	70.581	16	11.343	1.018	4.795
Molecule 35	C=CCc1ccc(c(c1O)C(=O)C)OCCCCCOc1ccc2c(c1)oc(cc2=O)C(=O)O	$C_{26}H_{26}O_8$	466.48	5.714590519	10.357	2	-0.27	76.164	20	28.065	1.128	9.589

Molecule 36	Oc1cccc(c1)OCCCCCOc1ccc2c(c1)oc(cc2=O)C(=O)O	$C_{21}H_{20}O_7$	384.38	5.408669523	9.368	2	-1.293	64.515	17	11.333	0.915	4.795
Molecule 37	OC(=O)c1cc(=O)c2c(o1)cc(cc2)OCCCCCOc1cccc(c1)OCc1ccccc1	$C_{28}H_{26}O_7$	474.5	5.676236217	9.055	1	-1.288	100.41	21	5.583	2.769	4.795
Molecule 38	COc1cccc(c1)OCCCCCOc1ccc2c(c1)oc(cc2=O)C(=O)O	$C_{22}H_{22}O_7$	398.41	5.424238972	9.001	1	-1.286	64.515	17	5.583	1.67	4.795
Molecule 39	CC(=O)c1ccc(cc1)OCCCCCOc1ccc2c(c1)oc(cc2=O)C(=O)O	$C_{23}H_{22}O_7$	410.42	5.069160472	9.001	1	0.025	70.078	18	11.366	1.497	9.589
Molecule 40	C=CCc1c(OCCCCCOc2ccc(cc2)NC(=O)C)ccc2c1oc(cc2=O)C(=O)O	$C_{26}H_{27}NO_7$	465.5	5.076855078	9.21	2	-0.12	76.667	19	11.49	2.176	9.589
Molecule 41	C=CCc1c(OCCCCCOc2cccc(c2C(=O)C)O)ccc2c1oc(cc2=O)C(=O)O	$C_{26}H_{26}O_8$	466.48	6.969863026	9.85	2	-0.265	76.164	20	28.065	1.067	9.589
Molecule 42	C=CCc1c(OCCCCCOc2ccc(cc2)C(=O)C)ccc2c1oc(cc2=O)C(=O)O	$C_{26}H_{26}O_7$	450.48	5.273464273	9.202	1	0.022	82.23	19	11.366	2.104	9.589
Molecule 43	C=CCc1c(OCCCCCOc2ccc(c(c2CC=C)O)C(=O)C)ccc2c1oc(cc2=O)C(=O)O	$C_{29}H_{30}O_8$	506.54	5.614708635	10.412	2	-0.221	88.316	21	28.065	1.88	9.589
Molecule 44	OC(=O)c1oc2cc(OCCCCCOc3ccc(c(c3)O)C(=O)C)ccc2c(=O)c1	$C_{23}H_{22}O_8$	426.42	4.797328653	9.796	2	-0.208	64.012	19	28.065	0.725	9.589
Molecule 45	CCCc1c(OCCCOc2ccccc2CCC)ccc2c1oc(cc2=O)C(=O)O	$C_{25}H_{28}O_6$	424.49	6.372594958	9.229	1	-1.27	69.575	18	16.772	3.062	4.795
Molecule 46	CCCc1cc2c(cc1OCCCOc1ccccc1CCC)oc(cc2=O)C(=O)O	$C_{25}H_{28}O_6$	424.49	4.372594959	9.147	1	-1.278	69.575	18	16.772	3.233	4.795
Molecule 47	CCCc1c(OCCCOc2ccc(c(c2CCC)O)C(=O)C)ccc2c1oc(cc2=O)C(=O)O	$C_{27}H_{30}O_8$	482.52	8.68351531	10.47	2	-0.204	63.006	21	22.679	2.172	9.589
Molecule 48	CCCc1c(OCC(=O)O)ccc(c1OCCCOc1ccc(c(c1CCC)O)C(=O)C)C(=O)C	$C_{27}H_{34}O_8$	486.55	5.698970004	10.484	2	-0.163	46.519	17	29.486	3.219	9.589
Molecule 49	CCCc1c(OCCCC(=O)O)ccc(c1OCCCOc1ccc(c(c1CCC)O)C(=O)C)C(=O)C	$C_{29}H_{38}O_8$	514.61	6.698970004	10.518	2	-0.119	46.519	17	35.907	3.751	14.384
Molecule 50	CCCc1c(OCCCCCC(=O)O)ccc(c1OCCCOc1ccc(c(c1CCC)O)C(=O)C)C(=O)C	$C_{31}H_{42}O_8$	542.66	6	10.545	2	-0.093	46.519	17	29.3	4.009	14.384
Molecule 51	CCCc1c(OCC(=O)O)ccc(c1OCCCCCOc1ccc(c(c1CCC)O)C(=O)C)C(=O)C	$C_{29}H_{38}O_8$	514.61	7	10.475	2	-0.132	46.519	17	17.316	3.58	9.589
Molecule 52	CCCc1c(OCCCC(=O)O)ccc(c1OCCCCCOc1ccc(c(c1CCC)O)C(=O)C)C(=O)C	$C_{31}H_{42}O_8$	542.66	6	10.502	2	-0.088	46.519	17	30.344	4.097	14.384
Molecule 53	CCCc1c(OCC(=O)O)ccc(c1OCCOCCOc1ccc(c(c1CCC)O)C(=O)C)C(=O)C	$C_{28}H_{36}O_9$	516.58	6.698970004	10.444	2	-0.173	46.519	17	49.307	3.141	9.589

Molecule 54	CCCc1c(OCCCC(=O)O)ccc(c1OCCOCCOc1ccc(c(c1CCC)O)C(=O)C)C(=O)C	$C_{30}H_{40}O_9$	544.63	7	10.471	2	-0.129	46.519	17	62.335	3.657	14.384
Molecule 55	CCCc1c(OCCCCCC(=O)O)ccc(c1OCCOCCOc1ccc(c(c1CCC)O)C(=O)C)C(=O)C	$C_{32}H_{44}O_9$	572.69	6.698970004	10.493	2	-0.103	46.519	17	55.728	3.907	14.384
Molecule 56	CCCc1c(OCC(=O)O)ccc(c1OCCOCCOCCOc1ccc(c(c1CCC)O)C(=O)C)C(=O)C	$C_{30}H_{40}O_{10}$	560.63	6	10.426	2	-0.166	46.519	17	42.7	3.24	9.589
Molecule 57	CCCc1c(OCCCC(=O)O)ccc(c1OCCOCCOCCOc1ccc(c(c1CCC)O)C(=O)C)C(=O)C	$C_{32}H_{44}O_{10}$	588.69	6.301029996	10.446	2	-0.122	46.519	17	55.728	3.743	14.384
Molecule 58	CCCc1c(OCC(=O)O)ccc(c1OCCOCCOCCOCCOc1ccc(c(c1CCC)O)C(=O)C)C(=O)C	$C_{32}H_{44}O_{11}$	604.69	6.522878745	10.42	2	-0.163	46.519	17	30.53	3.293	9.589
Molecule 59	CCCc1c(OCC(=O)O)ccc(c1OCCCOCCCOc1ccc(c(c1CCC)O)C(=O)C)C(=O)C	$C_{30}H_{40}O_9$	544.63	6.397940009	10.448	2	-0.144	46.519	17	17.316	3.475	9.589
Molecule 60	CCCc1c(OCCCC(=O)O)ccc(c1OCCCOCCCOc1ccc(c(c1CCC)O)C(=O)C)C(=O)C	$C_{32}H_{44}O_9$	572.69	6.698970004	10.47	2	-0.1	46.519	17	30.344	3.982	14.384
Molecule 61	CCCc1c(OCC(=O)O)ccc(c1OCCCCOCCCCOc1ccc(c(c1CCC)O)C(=O)C)C(=O)C	$C_{32}H_{44}O_9$	572.69	6.301029996	10.455	2	-0.124	46.519	17	17.316	3.691	9.589
Molecule 62	CCCc1c(OCCCOc2cccc(c2)OCC(=O)O)ccc(c1O)C(=O)C	$C_{22}H_{26}O_7$	402.44	6.301029996	10.352	2	-0.186	47.525	14	11.533	2.498	4.795
Molecule 63	CCCc1c(OCC(COc2ccc(c(c2CCC)O)C(=O)C)O)ccc2c1oc(cc2=O)C(=O)O	$C_{27}H_{30}O_9$	498.52	7.455931956	10.482	3	-0.255	63.006	22	41.279	1.42	9.589
Molecule 64	CCCc1c(OCCCOc2ccc3c(c2)cc(cc3)OCC(=O)O)ccc(c1O)C(=O)C	$C_{26}H_{28}O_7$	452.5	6.522878745	10.426	2	-0.18	59.658	19	11.533	2.717	4.795
Molecule 65	CCCc1c(OCCCOc2ccc3c(c2C(=O)C)cc(cc3)OCC(=O)O)ccc(c1O)C(=O)C	$C_{28}H_{30}O_8$	494.53	6.698970004	10.476	2	-0.188	59.155	21	29.486	2.541	9.589
Molecule 66	CCCc1c(OCCCCCOc2ccc3c(c2C(=O)C)cc(cc3)OCC(=O)O)ccc(c1O)C(=O)C	$C_{30}H_{34}O_8$	522.59	6.698970004	10.472	2	-0.156	59.155	21	17.316	2.84	9.589
Molecule 67	CCCc1c(OCCCOc2ccc3c(c2)cc(cc3)OCCCC(=O)O)ccc(c1O)C(=O)C	$C_{28}H_{32}O_7$	480.55	5.301029996	10.453	2	-0.174	59.658	19	17.954	3.002	9.589
Molecule 68	CCCc1c(OCCCOc2ccc3c(c2C(=O)C)cc(cc3)OCCCC(=O)O)ccc(c1O)C(=O)C	$C_{30}H_{34}O_8$	522.59	7.096910013	10.504	2	-0.143	59.155	21	35.907	2.887	14.384

Descriptors	Significance of 2D-QSAR descriptors
Smax33	A 3D shape-based descriptor representing the maximum surface area at 3.3 Å; reflects molecular shape and potential for target binding.
ndonr	Hydrogen bonding capacity, which affects solubility, permeability, and binding affinity.
Smax15	Similar to Smax33 but at a different radius (1.5 Å); reflects molecular topology and spatial distribution of atoms.
MRVSA6	Molecular Refractivity-Weighted Van der Waals Surface Area: Combines steric and polarizability information; linked to molecular interaction potential.
Qindex	A quantum chemical descriptor related to charge distribution or electronegativity; influences molecular reactivity and target interaction.
EstateVSA1	Hybrid descriptor combining E-state (electrotopological state) and VSA (van der Waals surface area); relates to electronic and steric properties.
S17	A topological or 2D shape descriptor that reflects molecular symmetry or atom pair distances; influences shape-based interactions with biological targets.
PEOEVSA1	A descriptor combining polar electrostatic overlap energy with van der Waals surface area; it reflects both electronic interactions a nd steric compatibility of the molecule.

Table S2. Molecular descriptor correlation matrix for QSAR model development

	Smax33	ndonr	Smax15	S17	PEOEVSA1	pIC50
**Smax33**	1	0.827155	0.721753	0.551484	0.677856	0.594393
**ndonr**	0.827155	1	0.63854	0.106867	0.564614	0.492027
**Smax15**	0.721753	0.63854	1	0.449908	0.857617	0.495781
**S17**	0.551484	0.106867	0.449908	1	0.525227	0.418615
**PEOEVSA1**	0.677856	0.564614	0.857617	0.525227	1	0.476395
**pIC50**	0.594393	0.492027	0.495781	0.418615	0.476395	1

Table S3. The actual IC_{50} values of 68 compounds were compared with the predicted values from multiple QSAR models, including multiple linear regressio n (MLR), multiple nonlinear regression (MNLR), and artificial neural network models (ANN_LM, ANN_SCG, ANN_BR). Notably, the ANN_SCG and ANN _BR models showed higher agreement with the experimental IC_{50} values, especially for compounds with very low or high activity, indicating their superior p redictive performance.

Compd.	IC_{50}	MLR Pred IC_{50}	MNLR Pred IC_{50}	ANN Pred IC_{50} (LM)	ANN Pred IC_{50} (SCG)	ANN Pred IC_{50} (BR)
1	3.09E-04	2.57E-05	3.16E-05	2.14E-05	1.45E-05	9.55E-06
2	1.05E-04	2.09E-05	2.09E-05	1.62E-05	1.20E-05	9.55E-06
3	4.79E-05	2.19E-05	1.29E-05	1.00E-05	1.23E-05	8.71E-06
4	2.04E-06	2.09E-05	1.20E-05	8.13E-06	1.23E-05	8.51E-06
5	1.45E-05	2.00E-05	8.71E-06	7.59E-06	1.23E-05	8.32E-06
6	9.33E-06	2.24E-05	1.29E-05	3.80E-06	9.12E-06	8.91E-06
7	1.07E-05	1.86E-05	6.31E-06	3.16E-06	8.13E-06	8.91E-06
8	2.14E-05	2.69E-05	4.37E-05	1.05E-05	1.15E-05	9.55E-06
9	4.68E-05	1.86E-05	1.74E-05	8.91E-06	9.77E-06	1.00E-05
10	4.17E-06	1.74E-05	1.38E-05	6.17E-06	9.33E-06	9.77E-06
11	1.10E-06	1.82E-05	9.12E-06	5.37E-06	9.77E-06	8.71E-06
12	4.57E-06	1.91E-05	7.41E-06	3.89E-06	1.05E-05	8.13E-06
13	4.27E-06	1.86E-05	6.61E-06	3.63E-06	1.05E-05	7.94E-06
14	1.05E-06	1.66E-05	2.63E-06	4.68E-07	6.46E-06	7.24E-06
15	7.94E-07	1.38E-05	2.40E-06	5.13E-07	6.03E-06	7.41E-06
16	1.66E-06	9.77E-06	5.89E-07	2.04E-07	3.98E-06	6.61E-06
17	1.48E-04	2.04E-05	2.29E-05	2.88E-05	1.55E-05	9.55E-06
18	2.45E-05	3.16E-07	1.45E-05	1.12E-05	1.26E-05	9.55E-06
19	2.00E-05	4.37E-06	2.04E-05	2.09E-05	1.02E-05	3.80E-06
20	6.76E-05	1.32E-06	3.31E-06	3.31E-05	8.51E-06	1.10E-06
21	2.24E-07	6.46E-07	1.20E-06	2.63E-07	9.55E-07	8.51E-07

22	7.41E-06	8.13E-07	1.02E-06	2.63E-06	8.32E-07	8.51E-07
23	1.02E-07	5.13E-07	1.15E-07	1.45E-07	8.13E-08	6.46E-07
24	1.78E-07	5.89E-07	2.09E-07	1.41E-07	7.08E-07	7.41E-07
25	1.02E-08	3.55E-07	3.72E-08	4.90E-09	1.41E-07	5.89E-07
26	5.13E-06	2.09E-06	2.88E-06	8.13E-06	1.58E-06	2.29E-06
27	1.00E-07	1.07E-06	3.02E-06	1.48E-06	8.32E-07	1.66E-06
28	4.57E-06	1.82E-06	4.90E-06	9.33E-06	5.13E-06	2.63E-06
29	3.72E-06	1.00E-06	6.76E-07	8.32E-07	6.76E-07	1.58E-06
30	8.91E-05	3.55E-07	2.40E-07	1.00E-05	2.75E-07	5.62E-07
31	3.16E-07	3.31E-07	3.80E-07	5.62E-06	3.89E-07	6.17E-07
32	1.86E-06	2.69E-07	4.47E-06	3.63E-06	3.31E-07	8.51E-07
33	6.61E-07	8.13E-07	1.91E-06	1.58E-06	2.45E-06	9.12E-07
34	1.17E-06	1.82E-05	9.12E-06	5.37E-06	9.77E-06	8.71E-06
35	1.95E-06	7.24E-07	6.61E-07	2.29E-09	2.09E-06	1.26E-06
36	3.98E-06	5.37E-06	1.58E-05	3.80E-06	3.31E-06	5.01E-06
37	2.14E-06	3.98E-06	3.63E-06	4.07E-07	1.15E-06	5.37E-06
38	3.80E-06	1.32E-05	9.55E-07	6.31E-07	3.02E-06	7.24E-06
39	8.71E-06	4.07E-06	9.12E-06	4.68E-05	5.50E-06	3.55E-06
40	8.51E-06	4.06E-06	7.24E-07	5.01E-06	1.78E-06	1.95E-06
41	1.10E-07	1.23E-06	7.24E-06	2.51E-06	3.39E-06	1.66E-06
42	5.37E-06	2.04E-06	7.59E-06	2.29E-06	3.47E-06	2.75E-06
43	2.45E-06	3.80E-07	3.55E-07	1.86E-06	7.94E-07	9.77E-07
44	1.62E-05	1.82E-06	4.37E-06	8.13E-06	7.41E-06	1.82E-06
45	4.27E-07	6.61E-06	2.04E-05	5.13E-07	1.35E-06	3.80E-06
46	4.27E-05	6.61E-06	1.45E-06	4.68E-07	1.12E-06	3.80E-06
47	2.09E-09	4.47E-07	4.17E-07	2.51E-09	5.62E-07	8.13E-07
48	2.04E-06	4.17E-07	2.63E-07	6.03E-07	5.62E-07	5.62E-07
49	2.04E-07	2.40E-07	3.63E-07	1.86E-07	3.89E-07	3.55E-07
50	1.00E-06	1.78E-07	2.95E-07	3.31E-07	3.24E-07	3.47E-07
51	1.00E-07	2.69E-07	5.50E-07	5.13E-07	4.37E-07	5.62E-07

52	1.00E-06	1.82E-07	3.39E-07	3.63E-07	3.02E-07	3.39E-07
53	2.04E-07	7.08E-07	3.39E-07	5.13E-07	5.25E-07	4.79E-07
54	1.00E-07	4.68E-07	2.82E-07	2.45E-07	2.88E-07	2.95E-07
55	2.04E-07	3.47E-07	4.90E-07	4.37E-07	2.40E-07	2.82E-07
56	1.00E-06	5.89E-07	2.63E-07	6.17E-07	5.01E-07	5.01E-07
57	5.01E-07	3.98E-07	1.62E-07	2.63E-07	2.95E-07	3.09E-07
58	3.02E-07	4.47E-07	3.09E-07	7.76E-07	5.37E-07	5.62E-07
59	4.07E-07	2.95E-07	3.72E-07	5.75E-07	4.79E-07	5.89E-07
60	2.04E-07	3.47E-07	5.75E-07	2.45E-07	3.47E-07	3.55E-07
61	5.01E-07	3.98E-07	4.68E-07	5.25E-07	3.98E-07	5.50E-07
62	5.01E-07	6.61E-07	3.89E-07	1.17E-05	1.17E-06	1.17E-06
63r	3.55E-08	3.55E-07	1.26E-08	4.90E-09	1.41E-07	5.89E-07
64	3.02E-07	4.47E-07	6.46E-07	4.68E-07	2.00E-07	9.77E-07
65	2.04E-07	4.68E-07	3.55E-07	7.59E-08	3.89E-07	6.61E-07
66	2.04E-07	3.09E-07	3.24E-07	2.45E-07	3.63E-07	6.61E-07
67	5.01E-06	2.95E-07	2.45E-07	1.51E-07	5.01E-07	6.76E-07
68	8.13E-08	2.88E-07	1.05E-07	6.03E-08	4.68E-07	4.47E-07

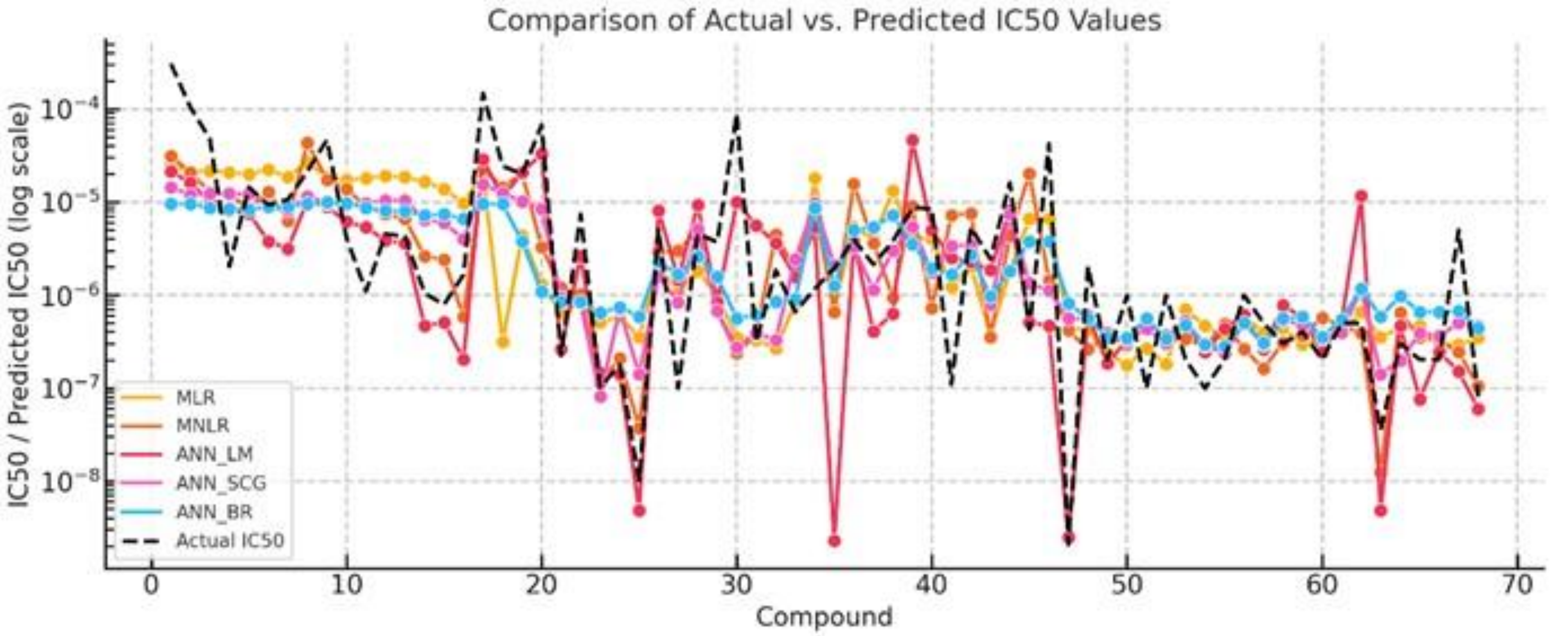

Figure S1. Comparison of actual IC_{50} values (black dashed line) with predicted IC_{50} values from multiple models—Multiple Linear Regression (MLR), Multipl e Non-Linear Regression (MNLR), and Artificial Neural Network models (ANN_LM, ANN_SCG, ANN_BR)—across 68 compounds. The plot is presented on a logarithmic scale to accommodate the wide dynamic range of IC_{50} values.

Table S4. Table: Top 10 prioritized compounds based on integrated evaluation of experimental pIC_{50}, QSAR-predicted pIC_{50} (ANN-LM model), and molecula r docking binding energy (BE).

Rank	Compound	Experimental pIC50	ANN-LM Predicted pIC50	Residual (Pred - Exp)	Binding Energy (kcal/mol)	Remarks
1	68	7.09	7.22	0.13	-8.8	Strong QSAR and docking; high confidence
2	23	6.99	6.84	-0.16	-9.5	Very good binder and QSAR match
3	27	7.0	5.83	-1.17	-9.4	Slight QSAR underprediction; strong docking
4	21	6.65	6.58	-0.08	-9.3	Consistent across models and docking
5	20	4.17	6.48	2.31	-9.0	Major predicted gain; validation needed
6	30	4.05	6.48	2.43	-9.2	High predicted gain; novel candidate
7	16	5.78	6.69	0.9	-6.4	QSAR favorable; docking moderate
8	22	5.13	5.58	0.44	-9.6	Very strong docking; moderate QSAR gain
9	37	5.67	6.39	0.71	-9.5	Solid overall performer; consistent
10	33	6.18	5.8	-0.38	-9.8	Excellent binding; slight QSAR underestimation
Zafirlukast, co-crystallized with the receptor, was used to define the binding site. Its binding score of -13.2 kcal/mol served as a reference for comparison with compounds in the QSAR study						Used for validation, not part of da taset
